# Using Quinolin-4-Ones as Convenient Common Precursors for a Metal-Free Total Synthesis of Both Dubamine and Graveoline Alkaloids and Diverse Structural Analogues

**DOI:** 10.3390/molecules29091959

**Published:** 2024-04-25

**Authors:** Rodrigo Abonia, Lorena Cabrera, Diana Arteaga, Daniel Insuasty, Jairo Quiroga, Paola Cuervo, Henry Insuasty

**Affiliations:** 1 Research Group of Heterocyclic Compounds, Department of Chemistry, Universidad del Valle, Cali A.A. 25360, Colombia; lorena.cabrera@correounivalle.edu.co (L.C.); diana.arteaga@correounivalle.edu.co (D.A.); jairo.quiroga@correounivalle.edu.co (J.Q.); pacuervop@unal.edu.co (P.C.); 2 Grupo de Investigación en Química y Biología, Departamento de Química y Biología, Universidad del Norte, Barranquilla A.A. 081007, Colombia; 3 Grupo de Estudios en Síntesis y Aplicaciones de Compuestos Heterocíclicos, Facultad de Ciencias, Departamento de Química, Universidad Nacional de Colombia, Bogotá A.A. 14490, Colombia; 4 Departamento de Química, Universidad de Nariño, Calle 18 No. 50-02 Torobajo, Pasto 520001, Colombia; hein@udenar.edu.co

**Keywords:** 2-aminochalcones, *Rutaceae* family, quinolines, metal-free conditions, Graveoline and Dubamine alkaloids, anticancer agents

## Abstract

The *Rutaceae* family is one of the most studied plant families due to the large number of alkaloids isolated from them with outstanding biological properties, among them the quinoline-based alkaloids Graveoline **1** and Dubamine **2**. The most common methods for the synthesis of alkaloids **1** and **2** and their derivatives involves cycloaddition reactions or metal-catalyzed coupling processes but with some limitations in scope and functionalization of the quinoline moiety. As a continuation of our current studies on the synthesis and chemical transformation of 2-aminochalcones, we are reporting here an efficient metal-free approach for the total synthesis of alkaloids **1** and **2** along with their analogues with structural diversity, through a two-step sequence involving intramolecular cyclization, oxidation/aromatization, *N*-methylation and oxidative C-C bond processes, starting from dihydroquinolin-4-ones as common precursors for the construction of the structures of both classes of alkaloids.

## 1. Introduction

Heterocyclic compounds containing quinoline nuclei have a special place in medicinal chemistry. They are substructures of more complex systems usually related to biologically active synthetic or naturally occurring products (mainly alkaloids) [1,2,3,4,5]. These nuclei have been considered as good starting materials for the synthesis of new compounds with a wide spectrum of biological activities such as antimycobacterial, antiparasitic, antibacterial, cytotoxic, antineoplastic, antimalarial, antiviral, antitumor, immunomodulatory, antiangiogenic, antileishmanial, antiarrhythmic, local anesthetic and anti-inflammatory activities [6,7,8,9,10].

**Figure 1:**
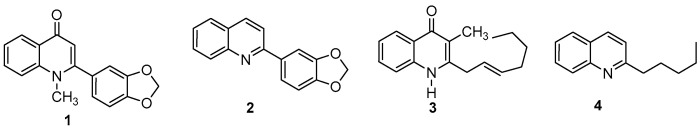
Structures of alkaloids Graveoline **1** and Dubamine **2** and the antibacterial compounds **3** and **4**.

Plants of the family *Rutaceae* are among the most studied, due to the large number of alkaloids that they provide and their pharmacological importance [11]. In particular, various studies on the *Haplopylum dubium* species have reported the existence of a series of quinoline-type alkaloids, among them Graveoline **1** and Dubamine **2**, as seen in Figure 1 [12].

Both compounds have displayed remarkable antimicrobial activity associated with their structural similarity to the quinolinic antifungal and molluscicidal agents **3** and **4**, respectively [13,14]. Additionally, Graveoline **1** has been identified as a stimulant of the CNS [15,16] and as phytotoxic [17], while Dubamine **2** has displayed antitumor activity, as shown in some previous studies [18].

**Scheme 1:**
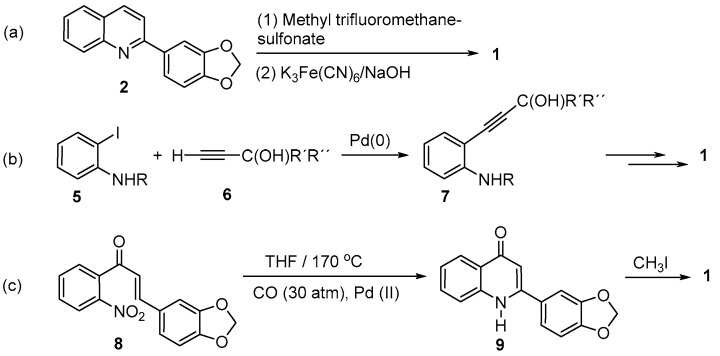
Some representative synthetic approaches for obtaining Graveoline **1**.

Some synthetic approaches have previously been reported to construct the structures of alkaloids **1** [18,19,20,21,22,23,24,25,26] and **2** [11,27,28,29,30,31,32,33,34,35,36]. Thus, Graveoline **1** and its derivatives were obtained through different strategies like the treatment of Dubamine **2** with a methylating agent and subsequent oxidation (Scheme 1a) [18]. Treatment of *o*-iodoanilines **5** with terminal acetylenic carbinols **6**, catalyzed by palladium, afforded acetylenic derivative **7**, which after several steps was converted into Graveoline **1** and its derivatives in moderate yields (Scheme 1b) [11]. The Pd-catalyzed reductive carbonylation of 2-nitrochalcones **8** under a CO atmosphere and toluene as a solvent afforded the alkaloid Norgraveoline **9** in a 78% yield; its subsequent treatment with CH_3_I led to the obtainment of the expected Graveoline **1** (Scheme 1c) [20].

**Scheme 2:**
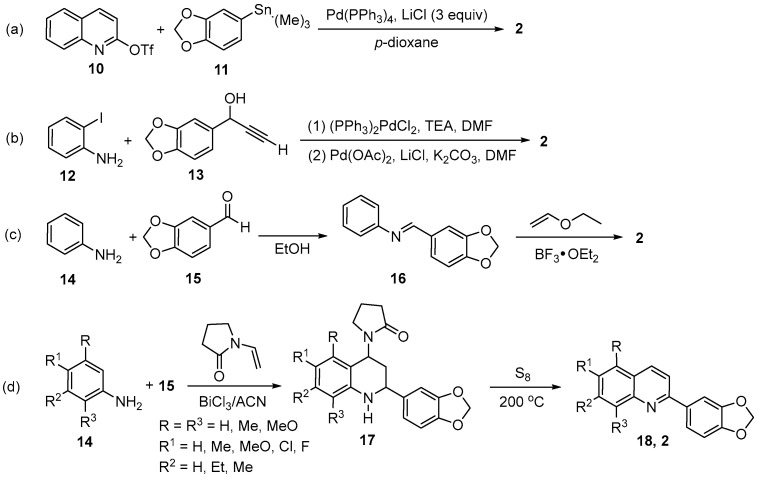
Some representative synthetic approaches for obtaining Dubamine **2** and its derivatives.

On the other hand, the most common methods for the synthesis of Dubamine **2** involve the coupling reaction of organic electrophiles with diverse organometallic complexes. Thus, the reaction of the triflate **10** with the tin derivative **11** led to the obtainment of alkaloid **2** in a 79% yield (Scheme 2a) [27]. Similarly, Dubamine **2** was obtained in a 44% yield from a Pd-catalyzed reaction of the *o*-iodoaniline **12** (Scheme 2b) [11]. A BF_3_-catalyzed reaction was also proposed for the synthesis of **2** starting from aniline **14**. Although this process proceeded via an imino Diels–Alder reaction between the imine **16** and vinyl ether, the authors could recover the expected alkaloid **2** in only a 1% yield (Scheme 2c) [28]. As an alternative to the previous inefficient process, Kouznetsov et al. proposed the synthesis of a series of quinolines **18** (including Dubamine **2**, when R = R^1^ = R^2^ = R^3^ = H) using the tetrahydroquinoline intermediates **17**, obtained from a Bi-catalyzed tri-component reaction, and their subsequent aromatization with sulfur at a high temperature, obtaining the target products in 40–62% overall yields (Scheme 2d) [29].

Although the usefulness of the metal-catalyzed coupling reactions described above for the synthesis of Graveoline **1** and Dubamine **2** is evident, it is also known that these strategies do not allow a diverse functionalization of the benzene ring of the quinoline moiety, which is a fundamental aspect for programs routed toward discovering and developing lead bioactive molecules inspired by quinoline-based drugs. Therefore, proposals of more efficient procedures of broader scope for the synthesis of these kinds of alkaloids and their derivatives are highly desired.

Thus, we are reporting here a metal-free alternative method for the total synthesis of Graveoline **1** and Dubamine **2** alkaloids and a series of their analogues starting from substituted dihydroquinolin-4-ones as common precursors for both kinds of alkaloidal frameworks.

## 2. Results and Discussion

As a continuation of our current studies directed toward the synthesis and chemical transformations of 2-aminochalcones [37,38,39,40], we planned to obtain a series of 2-aminochalcones **21**, along with their intramolecular cyclization products (i.e., the corresponding dihydroquinolin-4-ones **22**), as target intermediates to be evaluated as the key starting materials for developing of an alternative and short-step approach for the synthesis of Graveoline **1** and Dubamine **2** alkaloids, as well as a series of their structural analogues **23** and **24**, respectively.

**Scheme 3:**
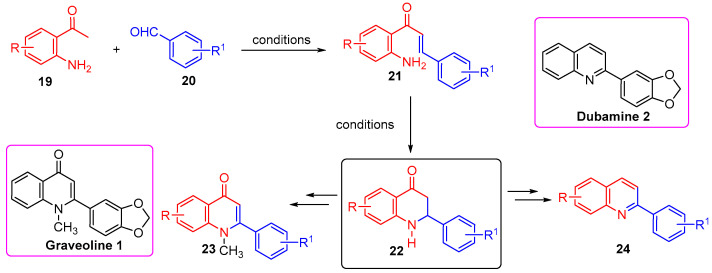
Proposed synthetic sketch of the synthesis of alkaloids Graveoline **1** and Dubamine **2** and their structural analogues **23** and **24**, respectively.

Before beginning the experiments, the synthesis of the target compounds **1**, **2**, **23** and **24** was visualized according to the following synthetic sketch shown in Scheme 3.

The present study was initiated with the synthesis of the starting 2-aminochalcones **21a**–**g** which were readily obtained in 60–97% yields by heating alcoholic solutions of equimolar amounts of *o*-aminoacetophenones **19a** (R = H) and the corresponding aryl aldehydes **20a**–**g** (see R^1^ in Table 1) in the presence of 20% aq NaOH (see Section 3 Materials and Methods) [37,38]. Subsequently, the intramolecular cyclization of chalcones **21**, catalyzed by Amberlyst^®^-15 [38], afforded the corresponding dihydroquinolin-4-ones **22a**–**h** in 65–94% yields (see Table 1 and Section 3 Materials and Methods).

All the starting chalcones **21** were yellow-to-orange solids, whereas their corresponding dihydroquinolin-4-ones **22** were pale yellow-colored compounds exhibiting strong fluorescence under exposure to long-wavelength UV irradiation in both solid-state and solution forms, in agreement with previous studies on these kinds of systems [41,42,43]. This characteristic easily permitted us to follow the reaction progress by TLC, as well as to check the purity of the key compounds **22**. The main spectroscopic features for compounds **22** corresponded to the presence of N-H and C=O absorption bands in the ranges of 3302–3336 cm^−1^ and 1606–1660 cm^−1^, respectively, in the IR spectra, in addition to two double-doublets for C-3(Ha)/C-3(Hb) protons [carbon] (in the ranges of 2.54–2.72/2.72–2.91 ppm and [45.2–46.0] ppm) and a double-doublet for the H-2 [C-2] proton [carbon] (in the ranges of 4.60–4.82 ppm and [56.0–57.3] ppm) in the ^1^H and ^13^C NMR spectra, respectively.

Once our key dihydroquinolin-4-ones **22** were synthesized, we turned our attention toward the Graveoline **1** and its analogues **23** as described in Scheme 3. For this purpose, we planned a sequential strategy consisting in an *N*-methylation of **22** followed by an oxidative C-C process to afford **23**. Thus, as a model reaction, a mixture of the dihydroquinolin-4-one **22a** (R = H, R^1^ = 4-Br) (0.5 g, 1.0 equiv), anhydrous Na_2_CO_3_ (1.5 equiv), CH_3_I (5.0 equiv) and *p*-dioxane (3 mL) was subjected to heating at 100 °C. After 72 h of heating, the total consumption of the starting material **22a** was not achieved and a complex mixture of products was detected by TLC. In a new experiment, the same reaction was repeated but *p*-dioxane was switched with DMF. After heating at 190 °C for 2 h, the starting compound **22a** was consumed (TLC control), the solvent was removed under reduced pressure, water (3 mL) was added to the residue and the product formed was extracted with ethyl acetate, affording the corresponding *N*-methyl derivative **25a** as a greenish fluorescent solid in a 68% yield. (Complete characterization data for compound **25a** are supplied in the Section 3 Materials and Methods). Subsequently, several attempts to oxidize compound **25a** were performed in order to obtain our target product **23a**. The results are summarized in Table 2. Initially, compound **25a** (0.3 g, 1.0 equiv) was treated with NBS (1.0 equiv) in MeOH in the presence of silica gel for 1 h to try and induce the α-bromination reaction [44,45]. After consumption of the starting compound **25a** (TLC control), the silica gel was filtered and the resulting solution was subjected to heating at 50 °C for two additional hours, in the presence of KOH, with the purpose of inducing a dehydrohalogenation process. However, a complex mixture of products was obtained after the signaled heating time (entry 1, Table 2). In a second experiment, a mixture of compound **25a** (1.0 equiv) and *p*-chloranil [46,47] (1.2 equiv) was subjected to reflux in DCM (3 mL) for 24 h (entry 2). Afterwards, the desired product **23a** was isolated, but at 8% only. In an attempt to improve the yield of product **23a**, the above reaction was repeated, switching DCM with DMF and heating for 2 h (entry 3). After removing the solvent under reduced pressure and purifying the resulting residue, the expected oxidized product **23a** was obtained as a yellow solid in a 61% yield.

Once this two-step sequence (i.e., *N*-methylation followed by the C-C oxidative process) was optimized for the synthesis of compound **23a**, this approach was extended to the remaining dihydroquinolin-4-ones **22b**–**g**. Reactions proceeded in a similar way and the products **23a**–**g** were obtained in 61–94% yields; see Table 3 and the Section 3 Materials and Methods.

The main spectroscopic features for compounds **23** corresponded to the absence of the N-H bands and the presence of C=O absorption bands in the range of 1606–1660 cm^−1^ in the IR spectra, as well as two singlets for N-CH_3_ and 3- =CH protons [carbons] (in the ranges of 3.61–3.98 [37.2–39.5] ppm and 6.26–6.91 [110.2–112.8] ppm) in the ^1^H and ^13^C NMR spectra, respectively.

Continuing with our purposes depicted in Scheme 3, the synthesis of Dubamine **2** and analogues **24** was planned in a three-step reduction/dehydration/oxidation sequence from the same key dihydroquinolin-4-ones **22**, as shown in Scheme 3 and Table 4. Thus, as a model reaction, a solution of dihydroquinolin-4-one **22a** (R = H, R^1^ = 4-Br) (0.5 g, 1.0 equiv) in MeOH (3 mL) was treated with NaBH_4_ (2.0 equiv) at room temperature in order to reduce the carbonyl group. Upon consumption of compound **22a** (TLC control), the solvent was removed under reduced pressure and the product was extracted with DCM to afford the expected 4-hydroxytetrahydroquinoline **26a** in a 90% yield and good purity. (Complete characterization data for compound **26a** are supplied in the Section 3 Materials and Methods). Subsequently, a sample of the derivative **26a** (0.3 g, 1.0 equiv) was subjected to a dehydration reaction with the aim of obtaining the dehydrated intermediate **27a**. Several attempts were performed, as shown in Table 4.

Initially, an open-vessel alkaline methanolic solution containing the previously obtained 4-hydroxyl-derivative **26a** was subjected to reflux (entry 1, Table 4). After 3 h of heating (TLC control), we noticed that the starting compound **26a** was not consumed; hence, the dehydration reaction did not proceed. In a second attempt, compound **26a** was similarly subjected to reflux in MeOH (3 mL) in the presence of *p*-toluenesulfonic acid (PTSA) (2.0 equiv) as a catalyst (entry 2, Table 4). After 2 h of heating (TLC control), the formation of a complex and inseparable mixture of products was observed. Then, the same reaction was repeated but MeOH was switched with dry toluene at reflux (entry 3, Table 4). After 3 h of heating, several products were formed and the main component of the mixture was isolated and purified by column chromatography. To our surprise and satisfaction, this product corresponded to our target aromatized compound **24a**, although in a 20% yield only. This finding indicated that the dehydration and oxidation processes proceeded sequentially in only one step. In this approach, the oxidant agent [O] should be the oxygen in the air (open-vessel conditions), potentialized by the stability gained by the molecule through the aromatization process of the dihydropyridine moiety of the intermediate **27a**. Pursuing an improvement in the reaction yield of compound **24a**, the above experiment was repeated using *p*-dioxane at room temperature instead of toluene (entry 4, Table 4). Interestingly, this variation afforded product **24a** in an 80% yield upon 2 h of stirring. It is worth mentioning that in neither of the cases of entries 3 and 4 could the dehydrated intermediate **27a** be detected (by TLC) or isolated, suggesting a very fast conversion of **27a** into the thermodynamic product **24a**.

With the optimized reaction conditions in hand, this two-step (i.e., reduction followed by a sequential dehydration/oxidation) procedure was extended to the remaining dihydroquinolin-4-ones **22b**–**g**. The reactions proceeded in a similar way and quinolines **24a**–**g** were obtained in 70–87% yields (Table 5 and Section 3 Materials and Methods).

The main spectroscopic features for compounds **24** corresponded to the absence of N-H, O-H and C=O absorption bands (signals) in their corresponding IR and NMR spectra. Complete (NMR, mass and elemental analysis) characterization data for compounds **24** are supplied in the Section 3 Materials and Methods.

**Scheme 4:**
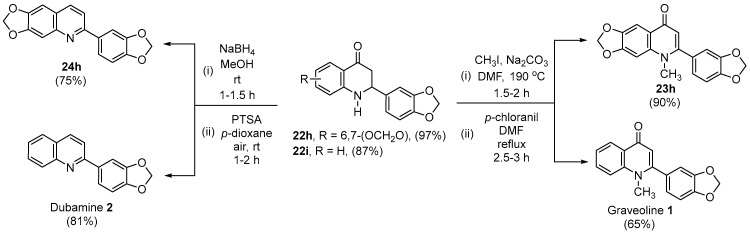
Total synthesis of Graveoline **1**, Dubamine **2** and their corresponding quinolinic-analogues (**23**,**24**)**h** from dihydroquinolin-4-ones **22h**,**i** through the two-step synthetic approaches developed in this research work.

In order to evaluate the practical synthetic usefulness of the above two developed approaches, we planned the synthesis of the dihydroquinolin-4-ones **22h**,**i** (R = OCH_2_O and H, respectively) and their subsequent transformation into Graveoline **1**, Dubamine **2** and their *bis*-dioxolo-derivatives **23h** and **24h**, respectively, as shown in Scheme 4.

The reactions proceeded in a similar way to that described in Table 3 and Table 5. Initially, dihydroquinolin-4-ones **22h,i** were obtained in 97% and 87% yields, respectively, from the intramolecular cyclization of their corresponding 2-aminochalcones **21h,i** (see Section 3 Materials and Methods). Subsequently, treatment of **22h,i** with CH_3_I followed by the oxidation process with *p*-chloranil afforded the expected Graveoline alkaloid **1** along with its *bis*-dioxolo-derivative **23h** in 65% and 90% yields, respectively. Alternatively, treatment of **22h, i** with NaBH_4_ followed by the PTSA-catalyzed dehydration/oxidation process in the presence of air afforded the expected Dubamine alkaloid **2** along with its *bis*-dioxolo-derivative **24h** in 81% and 75% yields, respectively. These findings demonstrate the synthetic usefulness of our established protocols.

It is very interesting that both dehydration/oxidation processes performed on compounds **22** occurred in only one step, simplifying the synthesis of Dubamine **1** and its analogues **24**. It is also remarkable that all products, Graveoline **1**, Dubamine **2** and their analogues **23** and **24**, respectively, were obtained in just a two-step sequence starting with dihydroquinolin-4-ones **22**. This fact became the main advantage of our metal-free approach in comparison to previous synthetic routes, which require more than two-step sequences and/or mediation of transition metal complexes.

## 3. Materials and Methods

Melting points were measured on a Büchi melting point apparatus (Flawil, Switzerland) and are uncorrected. All reactions were monitored by TLC with silica gel aluminum plates (Merck 60 F254, Hong Kong, China). Column chromatography was performed with Merck 230–400 mesh silica gel. IR spectra (KBr disks) were recorded on a Shimadzu FTIR 8400 spectrophotometer (Carlsbad, CA, USA). ^1^H and ^13^C NMR spectra were run on a Bruker Avance 400 spectrophotometer (Mannheim, Germany) operating at 400 and 100 MHz, respectively, using CDCl_3_ and (CD_3_)_2_SO as solvents and TMS as an internal standard. Mass spectra were obtained on a Shimadzu GCMS 2010-DI-2010 spectrometer (equipped with a direct inlet probe) operating at 70 eV (Carlsbad, CA, USA). Microanalyses were performed on an Agilent CHNS elemental analyzer (Santa Clara, CA, USA), and the values are within ±0.4% of the theoretical values. The starting reagents and solvents were purchased from Aldrich (St. Louis, MO, USA), Sigma (Kanagawa, Japan), Fluka (Buchs, Switzerland) and Merck (analytical reagent grades) and were used without further purification. Regarding the synthesis of the 2-aminochalcones **21**, these compounds were obtained from *o*-aminoacetophenones **19** and benzaldehydes **20** via a Claisen–Schmidt condensation reaction by following the procedure described in references [37,38].

*(E)-1-(2-Aminophenyl)-3-(4-bromophenyl)prop-2-en-1-one* **21a**: Yellow solid, 89% yield. M.p. 84–86 °C. FTIR (KBr): *ν* = [3398, 3300] (NH_2_), 3037, 2902, 1649 (C=O) cm^−1^. ^1^H NMR (400 MHz, DMSO-*d*_6_): *δ* = 6.54 (td, *J* = 7.9, *J* = 0.8 Hz, 1H), 6.82 (d, *J* = 8.3 Hz, 1H), 7.30 (td, *J* = 8.2, *J* = 1.2 Hz, 1H), 7.42 (bs, 2H, NH_2_), 7.59–7.69 (m, 3H, Ar-H × 2 and =CH), 7.83 (d, *J* = 8.4 Hz, 2H), 8.00 (d, *J* = 15.5 Hz, 1H, =CH), 8.09 (d, *J* = 7.5 Hz, 1H) ppm. ^13^C NMR (100 MHz, DMSO-*d*_6_): *δ* = 115.0, 117.4, 117.9 (Cq), 123.9 (Cq), 124.7, 130.7 (Cq), 131.0, 132.0, 132.3, 135.0, 141.0, 152.6 (Cq), 190.8 (C=O) ppm. MS (EI, 70 eV): *m*/*z* (%) = 303/301 (10.0/10.3) [M^+^], 302/300 (15.9/15.22), 146 (100).

*(E)-1-(2-Aminophenyl)-3-(4-chlorophenyl)prop-2-en-1-one* **21b**: Yellow solid, 97% yield. M.p. 94–96 °C. FTIR (KBr): *ν* = [3324, 3328] (NH_2_), 2990, 2882, 1646 (C=O) cm^−1^. ^1^H NMR (400 MHz, DMSO-*d*_6_): *δ* = 6.61 (td, *J* = 8.4, *J* = 1.0 Hz, 1H), 6.82 (dd, *J* = 8.4, *J* = 1.2 Hz, 1H), 7.30 (td, *J* = 8.4, *J* = 1.4 Hz, 1H), 7.42 (bs, 2H, NH_2_), 7.54 (d, *J* = 8.4, 2H), 7.63 (d, *J* = 15.5 Hz, 1H, =CH), 7.91 (d, *J* = 8.5 Hz, 2H), 7.99 (d, *J* = 15.5 Hz, 1H, =CH), 8.10 (dd, *J* = 8.4, *J* = 1.2 Hz, 1H) ppm. ^13^C NMR (100 MHz, DMSO-*d*_6_): *δ* = 114.9, 117.4, 117.9 (Cq), 124.7, 129.4, 130.8, 132.0, 134.5 (Cq), 134.9, 135.0 (Cq), 140.9, 152.6 (Cq), 190.8 (C=O) ppm. MS (EI, 70 eV): *m*/*z* (%) = 259/257 (6.8/20.4) [M^+^], 258/256 (12.9/31.4), 146 (100).

*(E)-1-(2-Aminophenyl)-3-(4-methoxyphenyl)prop-2-en-1-one* **21c**: Yellow solid, 60% yield. M.p. 70–72 °C. FTIR (KBr): *ν* = [3327, 3340] (NH_2_), 2989, 2982, 1649 (C=O) cm^−1^. ^1^H NMR (400 MHz, DMSO-*d*_6_): *δ* = 3.83 (s, 3H, OCH_3_), 6.60 (t, *J* = 7.7 Hz, 1H), 6.81 (d, *J* = 8.2 Hz, 1H), 7.01 (d, *J* = 8.2 Hz, 2H), 7.29 (t, *J* = 8.0 Hz, 1H), 7.34 (bs, 2H, NH_2_), 7.58 (d, *J* = 15.5 Hz, 1H, =CH), 7.82–7.85 (m, 3H, Ar-H × 2 and =CH), 8.08 (d, *J* = 8.2 Hz, 1H) ppm. ^13^C NMR (100 MHz, DMSO-*d*_6_): *δ* = 55.8 (OCH_3_), 114.5 114.9, 117.4, 118.2, 121.3 (Cq), 128.2, 130.9 (Cq), 131.7, 134.5, 142.4, 152.4 (Cq), 161.4 (Cq), 191.0 (C=O) ppm. MS (EI, 70 eV): *m*/*z* (%) = 253 (32) [M^+^], 252 (69), 146 (100).

*(E)-1-(2-Aminophenyl)-3-(p-tolyl)prop-2-en-1-one* **21d**: Yellow solid, 82% yield. M.p. 96–97 °C. FTIR (KBr): *ν* = [3327, 3330] (NH_2_), 2989, 2982, 1649 (C=O) cm^−1^. ^1^H NMR (400 MHz, DMSO-*d*_6_): *δ* = 2.35 (s, 3H, CH_3_), 6.60 (td, *J* = 8.1, *J* = 1.0 Hz, 1H), 6.81 (dd, *J* = 8.4, *J* = 0.8 Hz, 1H), 7.24–7.32 (m, 3H), 7.39 (bs, 2H, NH_2_), 7.63 (d, *J* = 15.5 Hz, 1H, =CH), 7.77 (d, *J* = 8.0 Hz, 2H), 7.91 (d, *J* = 15.5 Hz, 1H, =CH), 8.08 (dd, *J* = 8.2, *J* = 1.2 Hz, 1H) ppm. ^13^C NMR (100 MHz, DMSO-*d*_6_): *δ* = 21.6 (CH_3_), 114.9, 117.4, 118.1 (Cq), 122.8, 129.1, 130.0 (Cq), 131.8, 132.8, 134.7, 140.5 (Cq), 142.5, 152.5 (Cq), 190.1 (C=O) ppm. MS (EI, 70 eV): *m*/*z* (%) = 237 (28) [M^+^], 237 (47),146 (100).

*(E)-1-(2-Aminophenyl)-3-phenylprop-2-en-1-one* **21e**: Yellow solid, 64% yield. M.p. 174–177 °C. FTIR (KBr): *ν* = [3290, 3220] (NH_2_), 2989, 2982, 1649 (C=O) cm^−1^. ^1^H NMR (400 MHz, DMSO-*d*_6_): *δ* = 6.63 (t, *J* = 7.4 Hz, 1H), 6.83 (d, *J* = 8.3 Hz, 1H), 7.30 (t, *J* = 7.4 Hz, 1H), 7.38–7.50 (m, 5H, Ar-H × 3 and NH_2_), 7.66 (d, *J* = 15.5 Hz, 1H, =CH), 7.86 (d, *J* = 6.8 Hz, 2H), 7.96 (d, *J* = 15.5 Hz, 1H, =CH), 8.10 (d, *J* = 8.0 Hz, 1H) ppm. ^13^C NMR (100 MHz, DMSO-*d*_6_): *δ* = 115.0, 117.4, 118.0 (Cq), 123.9, 129.1, 129.4, 130.6, 131.9, 134.8, 135.5 (Cq), 142.4, 152.5 (Cq), 191.1 (C=O) ppm. MS (EI, 70 eV): *m*/*z* (%) = 223 (24) [M^+^], 222 (35), 146 (100).

*(E)-1-(2-Aminophenyl)-3-(3,4,5-trimethoxyphenyl)prop-2-en-1-one* **21f**: Yellow solid, 86% yield. M.p. 120–122 °C. FTIR (KBr): *ν* = [3327, 3340] (NH_2_), 2989, 2982, 1649 (C=O) cm^−1^. ^1^H NMR (400 MHz, DMSO-*d*_6_): *δ* = 3.73 (s, 3H, OCH_3_), 3.88 (s, 6H, OCH_3_), 6.62 (t, *J* = 7.8 Hz, 1H), 6.82 (d, *J* = 8.4 Hz, 1H), 7.20 (s, 2H), 7.30 (t, *J* = 8.0 Hz, 1H), 7.40 (bs, 2H, NH_2_), 7.61 (d, *J* = 15.5 Hz, 1H, =CH), 7.92 (d, *J* = 15.5 Hz, 1H, =CH), 8.13 (d, *J* = 8.1 Hz, 1H) ppm. ^13^C NMR (100 MHz, DMSO-*d*_6_): *δ* = 56.6 (OCH_3_), 60.6 (OCH_3_), 106.7, 114.9, 117.4, 118.1 (Cq), 123.1 (Cq), 131.1 (Cq), 132.0, 134.7, 139.8 (Cq), 142.9, 152.5 (Cq), 153.6 (Cq), 191.0 (C=O) ppm. MS (EI, 70 eV): *m*/*z* (%) = 313 (84) [M^+^], 312 (100), 146 (88).

*(E)-1-(2-Aminophenyl)-3-(3,4-dichlorophenyl)prop-2-en-1-one* **21g**: Yellow solid, 98% yield. M.p. 126–128 °C. FTIR (KBr): *ν* = [3327, 3345] (NH_2_), 2990, 2995, 1657 (C=O) cm^−1^. ^1^H NMR (400 MHz, DMSO-*d*_6_): *δ* = 6.63 (t, *J* = 7.9 Hz, 1H), 6.82 (d, *J* = 8.3 Hz, 1H), 7.31 (t, *J* = 8.2 Hz, 1H), 7.44 (bs, 2H, NH_2_), 7.60 (d, *J* = 15.4 Hz, 1H, =CH), 7.71 (d, *J* = 8.4 Hz, 1H), 7.86 (dd, *J* = 8.4, *J* = 1.9 Hz, 1H), 8.08 (d, *J* = 15.5 Hz, 1H, =CH), 8.14 (d, *J* = 8.1 Hz, 1H), 8.27 (d, *J* = 1.8 Hz, 1H) ppm. ^13^C NMR (100 MHz, DMSO-*d*_6_): *δ* = 114.9, 117.4, 117.8 (Cq), 126.1, 129.4, 130.4, 131.4, 132.1, 132.6 (Cq), 135.0, 136.4 (Cq), 136.5 (Cq), 139.6, 152.6 (Cq), 190.1 (C=O) ppm. MS (EI, 70 eV): *m*/*z* (%) = 295/293/291 (1.4/7.9/12) [M^+^], 294/292/290 (2.8/11/15), 146 (100).

*(E)-1-(6-Aminobenzo[d][1,3]dioxol-5-yl)-3-(benzo[d][1,3]dioxol-5-yl)prop-2-en-1-one* **21h**: Yellow solid, 70% yield. M.p. 148–151 °C. FTIR (KBr): *ν* = [3327, 3330] (NH_2_), 2989, 2982, 1649 (C=O), 1604 (C=C) cm^−1^. ^1^H NMR (400 MHz, DMSO-*d*_6_): *δ* = 5.95 (s, 2H, OCH_2_O), 5.99 (s, 2H, OCH_2_O), 6.32 (s, 1H), 6.96 (d, *J* = 8.0 Hz, 1H), 7.19 (s, 1H), 7.25 (dd, *J* = 8.1, *J* = 1.3 Hz, 1H), 7.36 (bs, 2H, NH_2_), 7.52 (d, *J* = 15.5 Hz, 1H, =CH), 7.69 (s, 1H), 7.74 (d, *J* = 15.5 Hz, 1H, =CH) ppm. ^13^C NMR (100 MHz, DMSO-*d*_6_): *δ* = 101.6 (OCH_2_O), 102.0 (OCH_2_O), 107.3, 108.6, 108.9, 109.3, 110.5 (Cq), 122.2, 125.6, 130.4 (Cq), 138.3 (Cq), 141.9, 148.5 (Cq), 149.3 (Cq), 152.1 (Cq), 153.3 (Cq), 188.2 (C=O) ppm. MS (EI, 70 eV): *m*/*z* (%) = 311 (4.5) [M^+^], 310 (4.7), 164 (100).

*(E)-1-(2-Aminophenyl)-3-(benzo[d][1,3]dioxol-5-yl)prop-2-en-1-one* **21i**: Yellow solid, 80% yield. M.p. 117–118 °C. FTIR (KBr): *ν* = [3425, 3309] (NH_2_), 3070, 2904, 1633 (C=O) cm^−1^. ^1^H NMR (400 MHz, DMSO-*d*_6_): *δ* = 6.11 (s, OCH2O, 2H), 6.60 (td, *J* = 8.0, *J* = 1.0 Hz, 1H), 6.81 (dd, *J* = 8.4, *J* = 1.0 Hz, 1H), 6.98 (d, *J* = 8.0 Hz, 1H), 7.26–7.30 (m, 2H), 7.38 (bs, 2H, NH_2_), 7.59 (d, *J* = 15.4 Hz, 1H, =CH), 7.63 (d, *J* = 1.6 Hz, 1H), 7.84 (d, *J* = 15.0 Hz, 1H, =CH), 8.11 (dd, *J* = 8.2, *J* = 1.1 Hz, 1H) ppm. ^13^C NMR (100 MHz, DMSO-*d*_6_): *δ* = 102.0 (OCH_2_O), 107.4, 108.9, 114.9, 117.3, 118.2 (Cq), 121.8, 125.8, 130.1 (Cq), 131.9, 134.6, 142.5, 148.6 (Cq), 149.6 (Cq), 152.5 (Cq), 191.0 (C=O) ppm. MS (EI, 70 eV): *m*/*z* (%) = 267 (31) [M^+^], 266 (51), 146 (100).

Synthesis of the dihydroquinolin-4-ones **22**: These compounds were obtained by the intramolecular cyclization of the 2-aminochalcones **21** in the presence of Amberlyst^®^-15 by following the procedure described in ref. [38].

*2-(4-Bromophenyl)-2,3-dihydroquinolin-4(1H)-one* **22a**: Yellow solid, 94% yield. M.p. 165–167 °C. FTIR (KBr): *ν* = 3306 (NH), 1647 (C=O), 1600 (C=C) cm^−1^. ^1^H NMR (400 MHz, DMSO-*d*_6_): *δ* = 2.70 (dd, *J* = 16.1, *J* = 4.0 Hz, 1H, Ha-3), 2.81 (dd, *J* = 16.1, *J* = 11.7 Hz, 1H, Hb-3), 4.78 (dd, *J* = 11.6, *J* = 4.4 Hz, 1H, H-2), 6.66 (t, *J* = 7.5 Hz, 1H), 6.90 (d, *J* = 8.3 Hz, 1H), 7.14 (bs, 1H, NH), 7.34 (td, *J* = 7.7, *J* = 4.0 Hz, 1H), 7.46 (d, *J* = 8.4 Hz, 2H), 7.59–7.62 (m, 3H) ppm. ^13^C NMR (100 MHz, DMSO-*d*_6_): *δ* = 45.5 (C-3), 56.0 (C-2), 116.8, 117.2, 118.2 (Cq), 121.2 (Cq), 126.8, 129.6, 131.9, 135.7, 141.6 (Cq), 152.7 (Cq), 192.7 (C=O) ppm. MS (70 eV): *m*/*z* (%) = 303/301 (41.4/41.6) [M^+^], 302/300 (22.5/16.2), 146 (100), 119 (37).

*2-(4-Chlorophenyl)-2,3-dihydroquinolin-4(1H)-one* **22b**: Yellow solid, 92% yield. M.p. 179–181 °C. FTIR (KBr): *ν* = 3302 (NH), 1647 (C=O), 1600 (C=C) cm^−1^. ^1^H NMR (400 MHz, DMSO-*d*_6_): *δ* = 2.69 (dd, *J* = 16.1, *J* = 4.3 Hz, 1H, Ha-3), 2.83 (dd, *J* = 16.1, *J* = 11.8 Hz, 1H, Hb-3), 4.79 (dd, *J* = 11.8, *J* = 4.4 Hz, 1H, H-2), 6.66 (td, *J* = 7.5, *J* = 4.0 Hz, 1H), 6.90 (d, *J* = 8.4 Hz, 1H), 7.15 (bs, 1H, NH), 7.34 (td, *J* = 7.7, *J* = 4.0 Hz, 1H), 7.46 (d, *J* = 8.6 Hz, 2H), 7.53 (d, *J* = 8.6 Hz, 2H), 7.61 (d, *J* = 7.9 Hz, 1H) ppm. ^13^C NMR (100 MHz, DMSO-*d*_6_): *δ* = 45.6 (C-3), 56.0 (C-2), 116.8, 117.1, 118.2 (Cq), 126.8, 129.0, 129.2, 132.7 (Cq), 135.6, 141.2 (Cq), 152.8 (Cq), 192.7 (C=O) ppm. MS (70 eV): *m*/*z* (%) = 259/257 (28.2/83) [M^+^], 258/256 (26/39), 146 (100), 119 (41).

*2-(4-Methoxyphenyl)-2,3-dihydroquinolin-4(1H)-one* **22c**: Yellow solid, 67% yield. M.p. 131–132 °C. FTIR (KBr): *ν* = 3329 (NH), 1606 (C=O, C=C), 1242 (C-O-C) cm^−1^. ^1^H NMR (400 MHz, DMSO-*d*_6_): *δ* = 2.63 (dd, *J* = 16.0, *J* = 4.0 Hz, 1H, Ha-3), 2.82 (dd, *J* = 16.0, *J* = 12.3 Hz, 1H, Hb-3), 3.77 (s, 3H, OCH_3_), 4.75 (dd, *J* = 12.2, *J* = 3.80 Hz, 1H, H-2), 6.65 (t, *J* = 7.4 Hz, 1H), 6.90 (d, *J* = 8.3 Hz, 1H), 6.96 (d, *J* = 8.6 Hz, 2H), 7.06 (bs, 1H, NH), 7.32 (t, *J* = 7.6 Hz, 1H), 7.42 (d, *J* = 8.6 Hz, 2H), 7.62 (d, *J* = 7.8 Hz, 1H) ppm. ^13^C NMR (100 MHz, DMSO-*d*_6_): *δ* = 45.9 (C-3), 55.6 (OCH_3_), 56.2 (C-2), 114.4, 116.8, 116.9, 118.2 (Cq), 126.8, 128.5, 134.1 (Cq), 135.5, 153.0 (Cq), 159.3 (Cq), 193.1 (C=O) ppm. MS (70 eV): *m*/*z* (%) = 253 (95) [M^+^], 252 (78), 146 (100), 119 (31).

*2-(p-Tolyl)-2,3-dihydroquinolin-4(1H)-one* **22d**: Yellow solid, 65% yield. M.p. 153–155 °C. FTIR (KBr): *ν* = 3309 (NH), 1649 (C=O), 1600 (C=C) cm^−1^. ^1^H NMR (400 MHz, DMSO-*d*_6_): *δ* = 2.63 (dd, *J* = 16.0, *J* = 3.5 Hz, 1H, Ha-3), 2.82 (dd, *J* = 16.0, *J* = 12.3 Hz, 1H, Hb-3), 3.39 (s, 3H, CH_3_), 4.72 (dd, *J* = 12.1, *J* = 3.7 Hz, 1H, H-2), 6.64 (t, *J* = 7.4 Hz, 1H), 6.90 (d, *J* = 8.3 Hz, 1H), 7.13 (bs, 1H, NH), 7.20 (d, *J* = 7.8 Hz, 2H), 7.33 (t, *J* = 7.6 Hz, 1H), 7.38 (d, *J* = 7.9 Hz, 2H), 7.61 (d, *J* = 7.6 Hz, 1H) ppm. ^13^C NMR (100 MHz, DMSO-*d*_6_): *δ* = 21.2 (CH_3_), 45.9 (C-3), 56.5 (C-2), 116.8, 116.9, 118.1 (Cq), 126.8, 127.2, 129.6, 135.6, 137.4 (Cq), 139.1 (Cq), 153.0 (Cq), 193.0 (C=O) ppm. MS (70 eV): *m*/*z* (%) = 237 (100) [M^+^], 236 (55), 146 (83), 119 (33).

*2-Phenyl-2,3-dihydroquinolin-4(1H)-one* **22e**: Yellow solid, 72% yield. M.p. 156–158 °C. FTIR (KBr): *ν* = 3334 (NH), 1654 (C=O), 1600 (C=C) cm^−1^. ^1^H NMR (400 MHz, DMSO-*d*_6_): *δ* = 2.67 (d, *J* = 15.9 Hz, 1H, Ha-3), 2.85 (t, *J* = 14.1 Hz, 1H, Hb-3), 4.77 (d, *J* = 12.0 Hz, 1H, H-2), 6.65 (t, *J* = 8.0 Hz, 1H), 6.91 (d, *J* = 8.2 Hz, 1H), 7.18 (bs, 1H, NH), 7.28–7.44 (m, 4H), 7.51 (d, *J* = 8.0 Hz, 2H), 7.62 (d, *J* = 7.7 Hz, 1H) ppm. ^13^C NMR (100 MHz, DMSO-*d*_6_): *δ* = 45.8 (C-3), 56.8 (C-2), 116.8, 117.0, 118.2 (Cq), 126.8, 127.4, 128.2, 129.0, 135.6, 142.2 (Cq), 152.9 (Cq), 192.9 (C=O) ppm. MS (70 eV): *m*/*z* (%) = 223 (100) [M^+^], 222 (43), 146 (95), 119 (29).

*2-(3,4,5-Trimethoxyphenyl)-2,3-dihydroquinolin-4(1H)-one* **22f**: Yellow solid, 90% yield. M.p. 142–144 °C. FTIR (KBr): *ν* = 3336 (NH), 1660 (C=O), 1595 (C=C), 1236 (C-O) cm^−1^. ^1^H NMR (400 MHz, DMSO-*d*_6_): *δ* = 2.63 (d, *J* = 16.0 Hz, 1H, Ha-3), 2.91 (bt, *J* = 14.7 Hz, 1H, Hb-3), 3.67 (s, 3H, OCH_3_), 3.80 (s, 6H, OCH_3_), 4.68 (dd, *J* = 13.1, *J* = 3.2 Hz, 1H, H-2), 6.66 (t, *J* = 7.4 Hz, 1H), 6.85 (s, 2H), 6.91 (d, *J* = 8.2 Hz, 1H), 7.06 (bs, 1H, NH), 7.33 (td, *J* = 7.7, *J* = 4.0 Hz, 1H), 7.63 (d, *J* = 7.9 Hz, 1H) ppm. ^13^C NMR (100 MHz, DMSO-*d*_6_): *δ* = 46.0 (C-3), 56.4 (OCH_3_), 57.3 (C-2), 60.5 (OCH_3_), 104.8, 116.8, 117.1, 118.2 (Cq), 126.8, 135.5, 137.4 (Cq), 137.7 (Cq), 153.0 (Cq), 153.3 (Cq), 193.1 (C=O) ppm. MS (70 eV): *m*/*z* (%) = 313 (31) [M^+^], 312 (14), 146 (29), 83 (100), 119 (6).

*2-(3,4-Dichlorophenyl)-2,3-dihydroquinolin-4(1H)-one* **22g**: Yellow solid, 92% yield. M.p. 122–123 °C. FTIR (KBr): *ν* = 3334 (NH), 1654 (C=O), 1600 (C=C) cm^−1^. ^1^H NMR (400 MHz, DMSO-*d*_6_): *δ* = 2.72 (dd, *J* = 16.1, *J* = 3.9 Hz, 1H, Ha-3), 2.86 (dd, *J* = 16.1, *J* = 11.8 Hz, 1H, Hb-3), 4.82 (dd, *J* = 11.7, *J* = 4.3 Hz, 1H, H-2), 6.68 (td, *J* = 7.5, *J* = 4.0 Hz, 1H), 6.90 (d, *J* = 8.2 Hz, 1H), 7.17 (bs, 1H, NH), 7.35 (td, *J* = 7.7, *J* = 4.0 Hz, 1H), 7.49 (dd, *J* = 8.4, *J* = 1.9 Hz, 1H), 7.61 (dd, *J* = 7.9, *J* = 1.3 Hz, 1H), 7.67 (d, *J* = 8.3 Hz, 1H), 7.78 (d, *J* = 1.9 Hz, 1H) ppm. ^13^C NMR (100 MHz, DMSO-*d*_6_): *δ* = 45.2 (C-3), 55.6 (C-2), 116.8, 117.3, 118.3 (Cq), 126.8, 127.8, 129.5, 130.6 (Cq), 131.2, 131.6 (Cq), 135.7, 143.4 (Cq), 152.6 (Cq), 192.5 (C=O) ppm. MS (70 eV): *m*/*z* (%) = 295/293/291 (14.2/84.1/100) [M^+^], 294/292/290 (19/51/45), 146 (98), 119 (36).

*6-(Benzo[d][1,3]dioxol-5-yl)-6,7-dihydro-[1,3]dioxolo [4,5-g]quinolin-8(5H)-one* **22h**: Yellow solid, 97% yield. M.p. >300 °C. FTIR (KBr): *ν* = 3327 (NH), 1649 (C=O), 1604 (C=C), 1236 (C-O-C) cm^−1^. ^1^H NMR (400 MHz, DMSO-*d*_6_): *δ* = 2.54 (dd, *J* = 16.0, *J* = 3.2 Hz, 1H, Ha-3), 2.72 (dd, *J* = 16.1, *J* = 12.7 Hz, 1H, Hb-3), 4.60 (dd, *J* = 12.7, *J* = 4.1 Hz, 1H, H-2), 5.97 (d, *J* = 6.0 Hz, 2H, OCH_2_O), 6.01 (bd, *J* = 1.0 Hz, 2H, OCH_2_O), 6.44 (s, 1H), 6.87–6.96 (m, 3H), 7.0 (s, 1H), 7.08 (bs, 1H, NH) ppm. ^13^C NMR (100 MHz, DMSO-*d*_6_): *δ* = 45.5 (C-3), 57.1 (C-2), 96.1, 101.5 (OCH_2_O), 101.7 (OCH_2_O), 103.7, 107.8, 108.6, 111.4 (Cq), 120.6, 136.0 (Cq), 140.6 (Cq), 147.2 (Cq), 147.8 (Cq), 151.4 (Cq), 154.1 (Cq), 190.7 (C=O) ppm. MS (70 eV): *m*/*z* (%) = 311 (99) [M^+^], 310 (70), 190 (100), 163 (33).

*2-(Benzo[d][1,3]dioxol-5-yl)-2,3-dihydroquinolin-4(1H)-one* **22i**: Yellow solid, 87% yield. M.p. 125–127 °C. FTIR (KBr): *ν* = 3327 (NH), 1649 (C=O), 1604 (C=C), 1236 (C-O-C) cm^−1^. ^1^H NMR (400 MHz, DMSO-*d*_6_): *δ* = 2.62 (ddd, *J* = 16.0, *J* = 4.1, *J* = 1.1 Hz, 1H, Ha-3), 2.83 (dd, *J* = 16.0, *J* = 12.3 Hz, 1H, Hb-3), 4.68 (dd, *J* = 12.3, *J* = 4.1 Hz, 1H, H-2), 6.01 (bd, *J* = 1.6 Hz, 2H, OCH_2_O), 6.65 (td, *J* = 7.9, *J* = 0.9 Hz, 1H), 6.58–6.97 (m, 3H), 7.05 (bs, 1H, NH), 7.09 (d, *J* = 1.5 Hz, 1H), 7.32 (td, *J* = 8.5, *J* = 1.6 Hz, 1H), 7.60 (dd, *J* = 7.9, *J* = 1.50 Hz, 1H) ppm. ^13^C NMR (100 MHz, DMSO-*d*_6_): *δ* = 45.9 (C-3), 56.5 (C-2), 101.5 (OCH_2_O), 107.8, 108.6, 116.7, 117.0, 118.2 (Cq), 120.6, 126.8, 135.5, 136.0 (Cq), 147.2 (Cq), 147.8 (Cq), 152.9 (Cq), 193.0 (C=O). MS (70 eV): *m*/*z* (%) = 267 (80) [M^+^], 266 (54), 146 (77), 83 (100), 119 (18).

General procedure for the synthesis Graveoline **1** and analogues **23**: (a) Methylation reaction: A mixture of dihydroquinolin-4-one **22** (1.0 equiv), anhydrous Na_2_CO_3_ (1.5 equiv), CH_3_I (5.0 equiv) and DMF (3 mL) was heated at 190 °C for 1–2 h. After the reaction was complete (TLC control), the solvent was removed under reduced pressure, water (3 mL) was added to the residue and product **25** was extracted with ethyl acetate. (b) Oxidation reaction: A mixture of N-methyl dihydroquinolin-4-one **25** (1.0 equiv), *p*-chloranil (1.2 equiv) and DMF was subjected to reflux for 2–3 h until complete consumption of the starting material **25** (TLC control). The solvent was removed under reduced pressure and the residue was purified by column chromatography on silica gel using a mixture of ethyl acetate/hexane (10:2) as an eluent to afford products **23**.

*2-(4-Bromophenyl)-1-methyl-2,3-dihydroquinolin-4(1H)-one* **25a**: Greenish solid, 68% yield. M.p. 98 °C. FTIR (KBr): *ν* = 1655 (C=O), 1604 (C=C) cm^−1^. ^1^H NMR (400 MHz, DMSO-*d*_6_): *δ* = 2.90 (dd, *J* = 16.1, *J* = 6.0 Hz, 1H, Ha-3), 2.97 (s, 3H, N-CH_3_), 3.19 (dd, *J* = 16.1, *J* = 6.2 Hz, 1H, Hb-3), 4.67 (bt, *J* = 6.0 Hz, 1H, H-2), 6.78–6.81 (m, 2H), 7.07 (d, *J* = 8.4 Hz, 2H), 7.43–7.57 (m, 3H), 7.89 (dd, *J* = 8.0, *J* = 1.4 Hz, 1H) ppm. ^13^C NMR (100 MHz, DMSO-*d*_6_): *δ* = 38.0 (N-CH_3_), 45.3 (C-3), 64.2 (C-2), 113.0, 117.0, 119.8 (Cq), 121.8 (Cq), 127.7, 128.3, 132.2, 136.2, 139.0 (Cq), 151.5 (Cq), 192.0 (C=O) ppm. MS (70 eV): *m*/*z* (%) = 317/315 (86.8/88.5) [M^+^], 160 (100).

*2-(4-Bromophenyl)-1-methylquinolin-4(1H)-one* **23a**: Yellow solid, 61% yield. M.p. 97–98 °C. FTIR (KBr): *ν* = 3120, 2918, 2849, 1618 (C=O) cm^−1^. ^1^H NMR (400 MHz, CDCl_3_): *δ* = 3.70 (s, 3H, N-CH_3_), 6.46 (s, 1H, H-3), 7.35 (d, *J* = 8.2, 2H), 7.51 (t, *J* = 7.6, 1H), 7.63 (d, *J* = 8.6, 1H), 7.70 (d, *J* = 8.2, 2H), 7.80 (t, *J* = 7.8, 1H), 8.52 (d, *J* = 7.9, 1H) ppm. ^13^C NMR (100 MHz, CDCl_3_): *δ* = 37.6 (N-CH_3_), 112.3 (C-3), 116.1, 124.4, 124.5 (Cq), 126.8, 130.2, 132.3, 132.9, 134.4 (Cq), 136.5 (Cq), 141.8 (Cq), 154.0 (Cq), 176.9 (C=O) ppm. MS (EI, 70 eV): *m*/*z* (%) = 315/313 (2.4/2.6) [M^+^], 86 (58), 84 (100). Anal. Calcd for C_16_H_12_BrNO: C, 61.17; H, 3.85; N, 4.46. Found: C, 60.98; H, 3.90; N, 4.62.

*2-(4-Chlorophenyl)-1-methylquinolin-4(1H)-one* **23b**: Yellow solid, 62% yield. M.p. 83–84 °C. FTIR (KBr): *ν* = 3072, 2938, 2837, 1599 (C=O) cm^−1^. ^1^H NMR (400 MHz, CDCl_3_): *δ* = 3.65 (s, 3H, N-CH_3_), 6.32 (s, 1H, H-3), 7.39 (d, *J* = 8.4, 2H), 7.47 (t, *J* = 7.5, 1H), 7.53 (d, *J* = 8.4, 2H), 7.63 (d, *J* = 8.6, 1H), 7.77 (t, *J* = 7.1, 1H), 8.54 (d, *J* = 8.0, 1H) ppm. ^13^C NMR (100 MHz, CDCl_3_): *δ* = 37.4 (N-CH_3_), 112.6 (C-3), 116.0, 124.1, 126.7 (Cq), 126.8, 129.2, 130.0, 132.7, 134.1 (Cq), 136.1 (Cq), 141.9 (Cq), 153.8 (Cq), 177.6 (C=O) ppm. MS (EI, 70 eV): *m*/*z* (%): = 271/269 (37/100) [M^+^], 241 (61). Anal. Calcd for C_16_H_12_ClNO: C, 71.25; H, 4.48; N, 5.19. Found: C, 71.12; H, 4.54; N, 5.30.

*2-(4-Methoxyphenyl)-1-methylquinolin-4(1H)-one* **23c**: Yellow solid, 75% yield. M.p. 193–194 °C. FTIR (KBr): *ν* = 3080, 2974, 2943, 1598 (C=O), [1249, 1078] C-O cm^−1^. ^1^H NMR (400 MHz, CDCl_3_): *δ* = 3.67 (s, 3H, N-CH_3_), 3.91 (s, 3H, OCH_3_), 6.34 (s, 1H, H-3), 7.04 (d, *J* = 8.0 Hz, 2H), 7.33 (d, *J* = 8.0 Hz, 2H), 7.45 (td, *J* = 8.0, *J* = 1.0 Hz, 1H), 7.59 (d, *J* = 8.6, 1H), 7.75 (td, *J* = 8.6, *J* = 3.2 Hz, 1H), 8.51 (dd, *J* = 8.1, *J* = 1.4 Hz, 1H) ppm. ^13^C NMR (100 MHz, CDCl_3_): *δ* = 37.5 (N-CH_3_), 55.5 (OCH_3_), 112.6 (C-3), 114.3, 116.1, 123.8, 126.7, 128.0 (Cq), 130.0 (x 2, Cq and CH), 132.4, 142.0 (Cq), 155.0 (Cq), 160.7 (Cq), 177.6 (C=O) ppm. MS (EI, 70 eV): *m*/*z* (%) = 265 (23) [M^+^], 237 (17), 222 (14), 85 (69), 83 (100). Anal. Calcd for C_17_H_15_NO_2_: C, 76.96; H, 5.70; N, 5.28. Found: C, 77.09; H, 5.92; N, 5.17.

*1-Methyl-2-(p-tolyl)quinolin-4(1H)-one* **23d**: Yellow solid, 94% yield. M.p. 86–87 °C. FTIR (KBr): *ν* = 3070, 2924, 2857, 1627 (C=O) cm^−1^. ^1^H NMR (400 MHz, CDCl_3_): *δ* = 2.48 (s, 3H, CH_3_), 3.77 (s, 3H, N-CH_3_), 6.52 (s, 1H, H-3), 7.28–7.36 (m, 4H), 7.48 (t, *J* = 8.2 Hz, 1H), 7.72 (d, *J* = 8.0 Hz, 1H), 7.85 (t, *J* = 8.0 Hz, 1H), 8.54 (d, *J* = 8.2 Hz, 1H) ppm. ^13^C NMR (100 MHz, CDCl_3_): *δ* = 21.4 (CH_3_), 38.1 (N-CH_3_), 111.4 (C-3), 116.4, 124.7, 126.6, 128.4, 129.6, 130.2 (Cq), 133.5, 136.4 (Cq), 137.5 (Cq), 139.3 (Cq), 140.6 (Cq), 187.4 (C=O) ppm. MS (EI, 70 eV): *m*/*z* (%) = 249 (45) [M^+^], 221 (44), 85 (71), 83 (100). Anal. Calcd for C_17_H_15_NO: C, 81.90; H, 6.06; N, 5.62. Found: C, 82.03; H, 6.23; N, 5.79.

*1-Methyl-2-phenylquinolin-4(1H)-one* **23e**: Yellow solid, 93% yield. M.p. 82–83 °C. FTIR (KBr): *ν* = 3110, 2925, 2859, 1650 (C=O) cm^−1^. ^1^H NMR (400 MHz, CDCl_3_): *δ* = 3.98 (s, 3H, N-CH_3_), 6.91 (s, 1H, H-3), 7.55 (d, *J* = 8.9, 2H), 7.62–7.64 (bd, 3H), 7.70 (t, *J* = 8.6, 1H), 7.93 (d, *J* = 7.8, 1H), 8.01 (t, *J* = 7.8, 1H), 8.62 (d, *J* = 7.9, 1H) ppm. NMR ^13^C (100 MHz, CDCl_3_): *δ* = 39.5 (N-CH_3_), 110.2 (C-3), 117.6, 126.3, 126.4 (×2, Cq and CH), 128.8, 129.4, 130.9, 133.7 (Cq), 134.8, 140.6 (Cq), 150.3 (Cq), 177.3 (C=O) ppm. MS (EI, 70 eV): *m*/*z* (%) = 235 (2.4) [M^+^], 149 (28), 57 (100). Anal. Calcd for C_16_H_13_NO: C, 81.68; H, 5.57; N, 5.95. Found: C, 81.53; H, 5.66; N, 6.04.

*1-Methyl-2-(3,4,5-trimethoxyphenyl)quinolin-4(1H)-one* **23f**: Yellow solid, 64% yield. M.p. 146–145 °C. FTIR (KBr): *ν* = 3070, 2934, 2836, 1676 (C=O), [1242, 1126] C-O cm^−1^. ^1^H NMR (400 MHz, CDCl_3_): *δ* = 3.86 (s, 3H, N-CH_3_), 3.92 (s, 6H, OCH_3_), 3.97 (s, 3H, OCH_3_), 6.71 (s, 1H, H-3), 7.57 (t, *J* = 7.4, 1H), 7.77 (d, *J* = 8.7, 1H), 7.89 (t, *J* = 8.7, 1H), 8.05 (s, 2H), 8.57 (d, *J* = 7.9, 1H) ppm. ^13^C NMR (100 MHz, CDCl_3_): *δ* = 38.7 (N-CH_3_), 56.8 (OCH_3_), 61.1 (OCH_3_), 106.2, 111.0 (C-3), 116.9, 125.3, 126.4, 130.2 (Cq), 133.7, 136.4 (Cq), 139.2 (Cq), 141.5 (Cq), 153.6 (Cq), 156.5 (Cq), 176.7 (C=O) ppm. MS (EI, 70 eV): *m*/*z* (%) = 325 (2.5) [M^+^], 167 (20), 149 (100). Anal. Calcd for C_19_H_19_NO_4_: C, 70.14; H, 5.89; N, 4.31. Found: C, 70.02; H, 5.96; N, 4.15.

*2-(3,4-Dichlorophenyl)-1-methylquinolin-4(1H)-one* **23g**: Yellow solid, 93% yield. M.p. 82–83 °C. FTIR (KBr): *ν* = 3102, 2940, 1626 (C=O) cm^−1^. ^1^H NMR (400 MHz, CDCl_3_): *δ* = 3.64 (s, 3H, N-CH_3_), 6.26 (s, 1H, H-3), 7.29 (dd, *J* = 7.2, *J* = 1.2 Hz, 1H), 7.47 (t, *J* = 7.5, 1H), 7.54–7.60 (m, 2H), 7.63 (d, *J* = 8.2, 1H), 7.76 (td, *J* = 7.3, *J* = 1.1 Hz, 1H), 8.50 (dd, *J* = 8.0, *J* = 1.3 Hz, 1H) ppm. ^13^C NMR (100 MHz, CDCl_3_): *δ* = 37.3 (N-CH_3_), 112.8 (C-3), 116.0, 124.0, 126.8, 126.9 (Cq), 127.9, 130.6, 131.0, 132.7, 133.4 (Cq), 134.4 (Cq), 135.6 (Cq), 141.9 (Cq), 152.1 (Cq), 177.5 (C=O) ppm. MS (EI, 70 eV): *m*/*z* (%) = 307/305/303 (0.5/2.1/3.3) [M^+^], 149 (21), 85 (77), 83 (100). Anal. Calcd for C_16_H_11_Cl_2_NO: C, 63.18; H, 3.65; N, 4.60. Found: C, 63.32; H, 3.74; N, 4.48.

*6-(Benzo[d][1,3]dioxol-5-yl)-5-methyl-[1,3]dioxolo[4,5-g]quinolin-8(5H)-one* **23h**: Yellow solid, 90% yield. M.p. 185–186 °C. FTIR (KBr): *ν* = 3040, 2989, 1658 (C=O), [1238, 1120] C-O cm^−1^. ^1^H NMR (400 MHz, CDCl_3_): *δ* = 3.61 (s, 3H, N-CH_3_), 6.09 (s, 2H, OCH_2_O), 6.13 (s, 2H, OCH_2_O), 6.26 (s, 1H, H-3), 6.88 (d, *J* = 1.3 Hz, 1H), 6.90 (dd, *J* = 7.8, *J* = 1.6 Hz, 1H), 6.94 (d, *J* = 7.9 Hz, 1H), 6.98 (s, 1H), 7.85 (s, 1H) ppm. ^13^C NMR (100 MHz, CDCl_3_): *δ* = 38.0 (N-CH_3_), 95.5, 101.7 (OCH_2_O), 102.1 (OCH_2_O), 103.9, 108.6, 109.1, 112.0 (C-3), 122.6 (Cq), 122.8, 129.4 (Cq), 139.2 (Cq), 145.5 (Cq), 148.0 (Cq), 148.7 (Cq), 152.4 (Cq), 153.2(Cq), 176.3 (C=O) ppm. MS (EI, 70 eV): *m*/*z* (%) = 323 (0.8) [M^+^], 279 (31), 167 (99), 149 (100). Anal. Calcd for C_18_H_13_NO_5_: C, 66.87; H, 4.05; N, 4.33. Found: C, 66.91; H, 3.96; N, 4.52.

*2-(Benzo[d][1,3]dioxol-5-yl)-1-methylquinolin-4(1H)-one* (Graveoline **1**): Yellow solid, 65% yield. M.p. 193–194 °C. FTIR (KBr): *ν* = 3160, 2919, 2854, 1623 (C=O), [1264, 1164] C-O cm^−1^. ^1^H NMR (400 MHz, CDCl_3_): *δ* = 3.68 (s, 3H, N-CH_3_), 6.11 (s, 2H, OCH_2_O), 6.35 (s, 1H, H-3), 6.91 (s, 1H), 6.92–6.98 (m, 2H), 7.47 (t, *J* = 7.5 Hz, 1H), 7.59 (d, *J* = 8.0 Hz, 1H), 7.76 (td, *J* = 11.4, *J* = 4.2 Hz, 1H), 8.53 (d, *J* = 7.1 Hz, 1H) ppm. ^13^C NMR (100 MHz, CDCl_3_): *δ* = 37.2 (N-CH_3_), 101.6 (OCH_2_O), 108.7, 109.3, 112.6 (C-3), 115.9, 122.7, 123.9, 126.6 (Cq), 126.8, 129.5 (Cq), 132.5, 142.0 (Cq), 148.0 (Cq), 148.8 (Cq), 154.4 (Cq), 177.1 (C=O) ppm. MS (EI, 70 eV): *m*/*z* (%) = 279 (80) [M^+^], 149 (100). Anal. Calcd for C_17_H_13_NO_3_: C, 73.11; H, 4.69; N, 5.02. Found: C, 72.98; H, 4.55; N, 5.00.

General procedure for the synthesis of Dubamine **2** and analogues **24**: Dihydroquinolin-4-one **22** (1.0 equiv) dissolved in methanol (3 mL) was subjected to reduction by treatment with NaBH_4_ (2.0 equiv), added portion-wise, for 1–2 h at room temperature. Then, the methanol was removed under reduced pressure and the crude was extracted with DCM (3 mL). After the DCM was removed under reduced pressure, the corresponding 4-hydroxyquinoline **26** was obtained in a quantitative yield. Subsequently, a mixture of 4-hydroxyquinoline **26** (1.0 equiv), *p*-dioxane (3 mL) and PTSA (2.0 equiv) was stirred for 2–3 h at room temperature. After the reaction was complete (TLC control), the solvent was removed under reduced pressure and the solid formed was purified by column chromatography on silica gel using a mixture of DCM/hexane (10:2) as an eluent to afford the desired compound **24**.

*2-(4-Bromophenyl)-1,2,3,4-tetrahydroquinolin-4-ol* **26a**: Pale yellow solid, 90% yield. M.p. 114–115 °C. FTIR (KBr): *ν* = 3420br (OH), 1602 (C=C), 1070 (C-O) cm^−1^. ^1^H NMR (400 MHz, DMSO-*d*_6_): *δ* = 1.74 (d, *J* = 8.3 Hz, 1H, OH), 2.02–2.08 (m, 1H, Ha-3), 2.36–2.41 (m, 1H, Hb-3), 3.97 (bs, 1H, NH), 4.55 (dd, *J* = 11.2, *J* = 2.6 Hz, 1H, H-2), 5.02–5.08 (m, 1H, H-4), 6.56 (d, *J* = 8.0 Hz, 1H), 6.79 (t, *J* = 7.4 Hz, 1H), 7.10 (t, *J* = 7.4 Hz, 1H), 7.32 (d, *J* = 8.4, 2H), 7.43 (d, *J* = 7.6 Hz, 1H), 7.50 (d, *J* = 8.4 Hz, 2H) ppm. ^13^C NMR (100 MHz, DMSO-*d*_6_): *δ* = 41.4 (C-3), 55.2 (C-2), 67.2 (C-4), 114.3, 118.3, 121.5 (Cq), 124.4 (Cq), 127.0, 128.3, 128.7, 131.9, 142.4 (Cq), 144.0 (Cq) ppm. MS (70 eV): *m*/*z* (%) = 305/303 (73.9/75.4) [M^+^], 287/285 (98.0/100.0) [M-H_2_O], 148 (87).

*2-(4-Bromophenyl)quinoline* **24a**: Yellow solid, 80% yield. M.p. 120–121 °C. FTIR (KBr): *ν* = 1539 (C=C), 1475 (C=N) cm^−1^. ^1^H NMR (400 MHz, DMSO-*d*_6_): *δ* = 7.54 (t, *J* = 7.4 Hz, 1H), 7.65 (d, *J* = 8.5 Hz, 2H), 7.74 (td, *J* = 7.7, *J* = 1.2 Hz, 1H), 7.80–7.85 (bd, 2H), 8.06 (d, *J* = 8.5 Hz, 2H), 8.18–8.25 (bt, 2H) ppm. ^13^C NMR (100 MHz, DMSO-*d*_6_): *δ* = 118.7, 124.2 (Cq), 126.7, 127.4 (Cq), 127.6, 129.3, 129.7, 130.1, 132.1, 137.3, 138.4 (Cq), 148.2 (Cq), 156.1 (Cq) ppm. MS (70 eV): *m*/*z* (%) = 285/283 (86/90) [M^+^], 204 (100). Anal. Calcd for C_15_H_10_BrN: C, 63.40; H, 3.55; N, 4.93. Found: C, 63.23; H, 3.41; N, 5.05.

*2-(4-Chlorophenyl)quinoline* **24b**: Beige solid, 75% yield. M.p. 115–116 °C. FTIR (KBr): *ν* = 1591 (C=C), 1485 (C=N) cm^−1^. ^1^H NMR (400 MHz, DMSO-*d*_6_): *δ* = 7.50 (d, *J* = 8.6 Hz, 2H), 7.54 (td, *J* = 7.5, *J* = 1.0 Hz, 1H), 7.75 (td, *J* = 7.7, *J* = 1.0 Hz, 1H), 7.80–7.85 (bd, 2H), 8.12 (d, *J* = 8.6 Hz, 2H), 8.17 (d, *J* = 8.5 Hz, 1H), 8.21 (d, *J* = 8.6 Hz, 1H) ppm. ^13^C NMR (100 MHz, DMSO-*d*_6_): *δ* = 118.6, 126.5, 127.3 (Cq), 127.5, 128.9, 129.0, 129.7, 129.9, 135.6 (Cq), 137.0, 138.1 (Cq), 148.3 (Cq), 156.0 (Cq) ppm. MS (70 eV): *m*/*z* (%) = 241/239 (32/100) [M^+^], 204 (67). Anal. Calcd for C_15_H_10_ClN: C, 75.16; H, 4.21; N, 5.84. Found: C, 75.23; H, 4.29; N, 5.75.

*2-(4-Methoxyphenyl)quinoline* **24c**: Beige solid, 73% yield. M.p. 122–123 °C. FTIR (KBr): *ν* = 1597 (C=C), 1492 (C=N),1246 (C-O) cm^−1^. ^1^H NMR (400 MHz, DMSO-*d*_6_): *δ* = 3.89 (s, 3H, OCH_3_), 7.06 (d, *J* = 8.8 Hz, 2H), 7.51 (t, *J* = 7.5 Hz, 1H), 7.72 (td, *J* = 7.7, *J* = 1.0 Hz, 1H), 7.78–7.86 (bt, 2H), 8.12–8.21 (m, 4H) ppm. ^13^C NMR (100 MHz, DMSO-*d*_6_): *δ* = 55.4 (OCH_3_), 114.3, 118,6, 126.0, 127.0 (Cq), 127.5, 129.0, 129.5, 129.7, 132.2 (Cq), 136.8, 148.2 (Cq), 156.9 (Cq), 160.9 (Cq) ppm. MS (70 eV): *m*/*z* (%) = 235 (100) [M^+^], 220 (31), 192 (34), 191 (35). Anal. Calcd for C_16_H_13_NO: C, 81.68; H, 5.57; N, 5.95. Found: C, 81.57; H, 5.36; N, 6.03.

*2-(p-Tolyl)quinoline* **24d**: Yellow solid, 85% yield. M.p. 83–84 °C. FTIR (KBr): *ν* = 1595 (C=C), 1494 (C=N) cm^−1^. ^1^H NMR (400 MHz, DMSO-*d*_6_): *δ* = 2.45 (s, 3H, CH_3_), 7.35 (d, *J* = 8.1 Hz, 2H), 7.53 (t, *J* = 7.5 Hz, 1H), 7.74 (t, *J* = 7.7 Hz, 1H), 7.83 (d, *J* = 8.0 Hz, 1H), 7.87 (d, *J* = 8.6 Hz, 1H), 8.10 (d, *J* = 8.1 Hz, 2H), 8.20–8.27 (bt, 2H) ppm. ^13^C NMR (100 MHz, DMSO-*d*_6_): *δ* = 21.4 (CH_3_), 119.0, 126.3, 127.1 (Cq), 127.5, 127.6, 129.4, 129.6, 129.8, 136.5 (Cq), 137.0, 139.7 (Cq), 148.0 (Cq), 157.3 (Cq) ppm. MS (70 eV): *m*/*z* (%) = 219 (100) [M^+^], 204 (39). Anal. Calcd for C_16_H_13_N: C, 87.64; H, 5.98; N, 6.39. Found: C, 87.57; H, 6.05; N, 6.44.

*2-Phenylquinoline* **24e**: Beige solid, 70% yield. M.p. 82–83 °C. FTIR (KBr): *ν* = 1595 (C=C), 1489 (C=N) cm^−1^. ^1^H NMR (400 MHz, DMSO-*d*_6_): *δ* = 7.48 (t, *J* = 7.1 Hz, 1H), 7.52–7.58 (m, 3H), 7.75 (t, *J* = 7.6 Hz, 1H), 7.84 (d, *J* = 8.1 Hz, 1H), 7.89 (d, *J* = 8.6 Hz, 1H), 8.16–8.25 (m, 4H) ppm. ^13^C NMR (100 MHz, DMSO-*d*_6_): *δ* = 119.1, 126.3, 127.2 (Cq), 127.5, 127.6, 128.9, 129.4, 129.7, 129.8, 136.8, 139.7 (Cq), 148.3 (Cq), 157.4 (Cq) ppm. MS (70 eV): *m*/*z* (%) = 205 (100) [M^+^], 204 (94). Anal. Calcd for C_15_H_11_N: C, 87.77; H, 5.40; N, 6.82. Found: C, 87.50; H, 5.28; N, 6.86.

*(3,4,5-Trimethoxyphenyl)quinoline* **24f**: Yellow solid, 80% yield. M.p. 90–93 °C. FTIR (KBr): *ν* = 1593 (C=C), 1496 (C=N), 1244 (C-O) cm^−1^. ^1^H NMR (400 MHz, DMSO-*d*_6_): *δ* = 3.93 (s, 3H, OCH_3_), 4.01 (s, 6H, OCH_3_), 7.42 (s, 2H), 7.53 (t, *J* = 7.5 Hz, 1H), 7.74 (t, *J* = 7.5 Hz, 1H), 7.81–7.85 (m, 2H), 8.20–8.24 (bd, 2H) ppm. ^13^C NMR (100 MHz, DMSO-*d*_6_): *δ* = 56.4 (OCH_3_), 61.0 (OCH_3_), 105.0, 118.9, 126.4, 127.2 (Cq), 127.5, 129.5, 129.9, 135.0 (Cq), 137.0, 139.6 (Cq), 147.9 (Cq), 153.6 (Cq), 156.9 (Cq) ppm. MS (70 eV): *m*/*z* (%) = 295 (100) [M^+^], 280 (49), 222 (38). Anal. Calcd for C_18_H_17_NO_3_: C, 73.20; H, 5.80; N, 4.74. Found: C, 73.48; H, 5.65; N, 4.82.

*2-(3,4-Dichlorophenyl)quinoline* **24g**: Yellow solid, 87% yield. M.p. 107–108 °C. FTIR (KBr): *ν* = 1593 (C=C), 1543 (C=N) cm^−1^. ^1^H NMR (400 MHz, DMSO-*d*_6_): *δ* = 7.53–7.60 (m, 2H), 7.76 (t, *J* = 7.7 Hz, 1H), 7.79–7.86 (bt, 2H), 8.00 (dd, *J* = 8.4, *J* = 1.8 Hz, 1H), 8.16 (d, *J* = 8.5 Hz, 1H), 8.23 (d, *J* = 8.6 Hz, 1H), 8.32 (d, *J* = 1.7 Hz, 1H) ppm. ^13^C NMR (100 MHz, DMSO-*d*_6_): *δ* = 118.3, 126.6, 126.8, 127.4 (Cq), 127.5, 129.4, 129.8, 130.0, 130.7, 133.2 (Cq), 133.6 (Cq), 137.2, 139.5 (Cq), 148.2 (Cq), 154.6 (Cq) ppm. MS (70 eV): *m*/*z* (%) = 277/275/273 (12/66/100) [M^+^], 238 (67), 203 (29). Anal. Calcd for C_15_H_9_Cl_2_N: C, 65.72; H, 3.31; N, 5.11. Found: C, 65.82; H, 3.13; N, 4.98.

*6-(Benzo[d][1,3]dioxol-5-yl)-[1,3]dioxolo[4,5-g]quinoline* **24h**: Pink solid, 75% yield. M.p. 195–196 °C. FTIR (KBr): *ν* = 1581 (C=C), 1481 (C=N), 1253 (C-O-C) cm^−1^. ^1^H NMR (400 MHz, DMSO-*d*_6_): *δ* = 6.03 (s, 2H, OCH_2_O), 6.10 (s, 2H, OCH_2_O), 6.93 (d, *J* = 8.1 Hz, 1H), 7.04 (s, 1H), 7.41 (s, 1H), 7.58–7.64 (m, 2H), 7.67 (d, *J* = 1.6 Hz, 1H), 7.96 (d, *J* = 8.5 Hz, 1H) ppm. ^13^C NMR (100 MHz, DMSO-*d*_6_): *δ* = 101.3 (OCH_2_O), 101.7 (OCH_2_O), 102.6, 106.1, 107.7, 108.5, 116.8, 121.3, 123.9 (Cq), 134.3 (Cq), 135.5, 146.5 (Cq), 147.6 (Cq), 148.3 (Cq), 148.5 (Cq), 150.8 (Cq), 154.7 (Cq) ppm. MS (70 eV): *m*/*z* (%) = 293 (100) [M^+^], 177 (19). Anal. Calcd for C_17_H_11_NO_4_: C, 69.62; H, 3.78; N, 4.78. Found: C, 69.78; H, 3.86; N, 4.81.

*2-(Benzo[d][1,3]dioxol-5-yl)quinoline* (Dubamine **2**): Pink solid, 81% yield. M.p. 93–94 °C. FTIR (KBr): *ν* = 1593 (C=C), 1485 (C=N), 1250 (C-O-C) cm^−1^. ^1^H NMR (400 MHz, DMSO-*d*_6_): *δ* = 6.05 (s, 2H, OCH_2_O), 6.96 (d, *J* = 8.1 Hz, 1H), 7.51 (t, *J* = 7.4 Hz, 1H), 7.67 (dd, *J* = 8.1, *J* = 1.7 Hz, 1H), 7.72 (td, *J* = 7.7, *J* = 1.7 Hz, 1H), 7.76 (d, *J* = 1.4 Hz, 1H), 7.77–7.82 (m, 2H), 8.10–8.20 (bt, 2H) ppm. ^13^C NMR (100 MHz, DMSO-*d*_6_): *δ* = 101.4 (OCH_2_O), 108.0, 108.5, 118.6, 121.8, 126.1, 127.0 (Cq), 127.4, 129,6, 129.7, 134.2 (Cq), 136.7, 148.2 (Cq), 148.4 (Cq), 148.9 (Cq), 156.7 (Cq) ppm. MS (70 eV): *m*/*z* (%) = 249 (100) [M^+^], 191 (52). Anal. Calcd for C_16_H_11_NO_2_: C, 77.10; H, 4.45; N, 5.62. Found: C, 77.22; H, 4.36; N, 5.80.

## 4. Conclusions

We have successfully developed a useful and metal-free alternative method for the total synthesis of Graveoline **1** and Dubamine **2** alkaloids along with their analogue products **23** and **24**, respectively. In both cases, the synthesis was efficiently achieved in just a two-step sequence starting from the dihydroquinolin-4-ones **22** as common precursors for both classes of alkaloidal structures.

## Figures and Tables

**Table 1:** Synthesis of 2-aminochalcones **21** and the key dihydroquinolin-4-ones **22**.

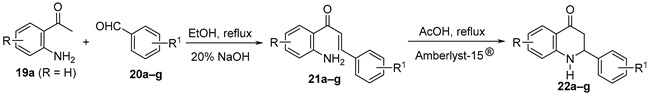
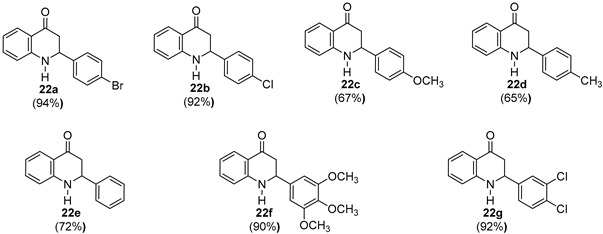

**Table 2:** Optimization of the C-C oxidative process performed on compound **25a** for the synthesis of product **23a**.

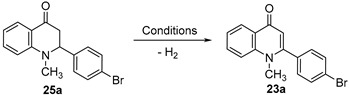			
Entry	Conditions	Reaction Time (h)	Yield (%)
1	(1) NBS/MeOH/silica gel (2) KOH/MeOH/50 °C	3	Complex mixture
2	*p*-chloranil/DCM/reflux	24	8
3	*p*-chloranil/DMF/reflux	2	61

**Table 3:** Synthesis of Graveoline-analogues **23** via a two-step *N*-methylation/C-C oxidation sequence from dihydroquinolin-4-ones **22**.

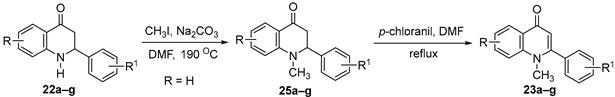
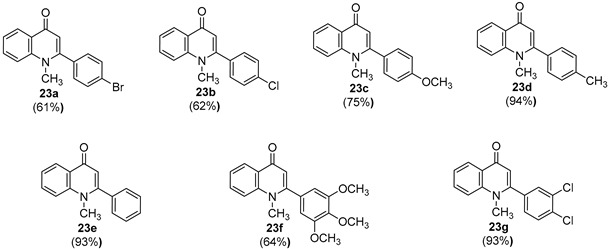

**Table 4:** Optimization of the dehydration/oxidation process for the synthesis of quinoline-derivative **24a** from the 4-hydroxytetrahydroquinoline **26a**.

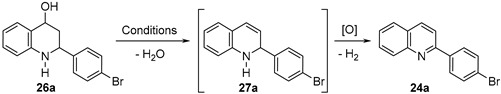			
Entry	Conditions	Reaction Time (h)	Yield (%) ^a^ of 27a/24a
1	MeOH/B(OH)_3_/reflux	3	NR
2	MeOH/PTSA/reflux	2	Complex mixture
3	Toluene/PTSA/reflux/air	3	ND/20
4	*p*-dioxane/PTSA/air/rt	2	ND/80

^a^ NR = no reaction; ND = not detected.

**Table 5:** Synthesis of Dubamine-analogues **24** from dihydroquinolin-4-ones **22** via a two-step reduction followed by a dehydration/oxidation sequence.

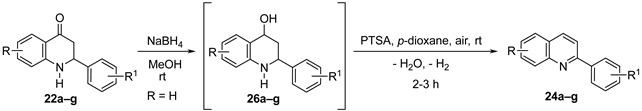
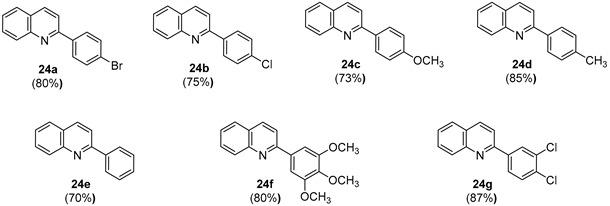

## Data Availability

The data presented in this study are available in article and Supplementary Materials.

## Supplementary Materials

The following supporting information can be downloaded at https://www.mdpi.com/article/10.3390/molecules29091959/s1. Copies of ^1^H, ^13^C and DEPT-135 NMR spectra for compounds **22a**–**i**, **23a**–**h**, **24a**–**h**, **26a**, Graveoline (**1**) and Dubamine (**2**).
