# Supplementary material for: Using Quinolin-4-Ones as Convenient Common Precursors for a Metal-Free Total Synthesis of Both Dubamine and Graveoline Alkaloids and Diverse Structural Analogues
Source: Molecules. 2024 Apr 25;29(9):1959. doi: 10.3390/molecules29091959 (PMC11085558; doi:10.3390/molecules29091959)

Supporting Information

Using Quinolin-4-ones as Convenient Common Precursors for a Metal-free Total Synthesis of Both Dubamine and Graveoline Alkaloids and Diverse Structural Analogues

Rodrigo Abonia [1,*] Lorena Cabrera [1], Diana Arteaga [1], Daniel Insuasty [1,2,*], Jairo Quiroga [1], Paola Cuervo [1,3], and Henry Insuasty [4]

[1] Research Group of Heterocyclic Compounds, Department of Chemistry, Universidad del Valle, A. A. 25360, Cali, Colombia; rodrigo.abonia@correounivalle.edu.co; lorena.cabrera@correounivalle.edu.co; diana.arteaga@correounivalle.edu.co; jairo.quiroga@correounivalle.edu.co

[2] Grupo de Investigación en Química y Biología, Departamento de Química y Biología, Universidad del Norte, Barranquilla Atlántico 081007, Colombia; insuastyd@uninorte.edu.co.

[3] Grupo de Estudios en Síntesis y Aplicaciones de Compuestos Heterocíclicos, Universidad Nacional de Colombia, Facultad de Ciencias, Departamento de Química, AA 14490, Bogotá, Colombia; pacuervop@unal.edu.co

[4] Departamento de Química, Universidad de Nariño, Calle 18 No. 50-02 Torobajo, Pasto, Colombia; hein@udenar.edu.co

* Correspondence: rodrigo.abonia@correounivalle.edu.co

Table of Contents

^{1}H and ^{13}C NMR spectra for compounds **22a-i**, **23a-h**, **24a-h**, **26a**, Graveoline (**1**) and Dubamine (**2**) S2

22a

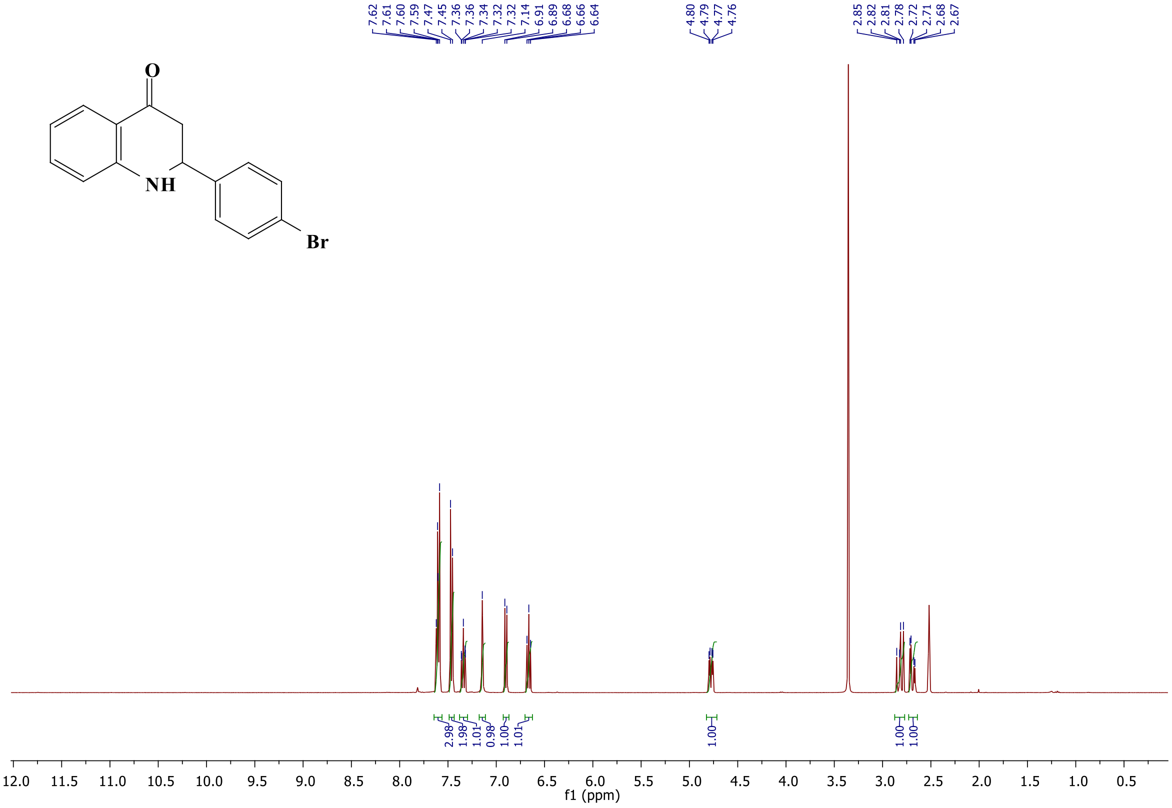
7.62
7.61
7.60
7.59
7.47
7.45
7.36
7.36
7.34
7.32
7.32
7.14
6.91
6.89
6.68
6.66
6.64
4.80
4.79
4.77
4.76
2.85
2.82
2.81
2.78
2.72
2.71
2.68
2.67
O
NH
Br
2.98
1.98
1.01
0.98
1.00
1.01
1.00
1.00
1.00
12.0 11.5 11.0 10.5 10.0 9.5 9.0 8.5 8.0 7.5 7.0 6.5 6.0 5.5 5.0 4.5 4.0 3.5 3.0 2.5 2.0 1.5 1.0 0.5
f1 (ppm)

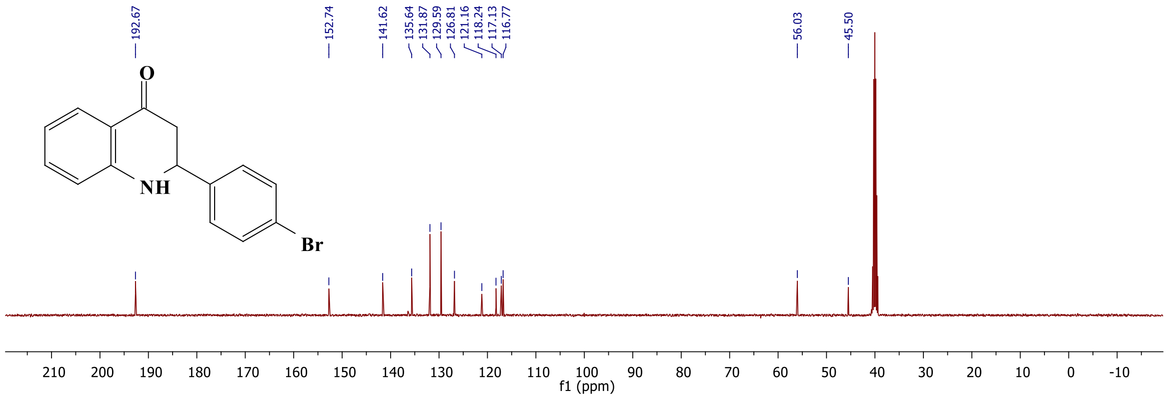
192.67
152.74
141.62
135.64
131.87
129.59
126.81
121.16
118.24
117.13
116.77
56.03
45.50
O
NH
Br
210 200 190 180 170 160 150 140 130 120 110 100 90 80 70 60 50 40 30 20 10 0 -10
f1 (ppm)

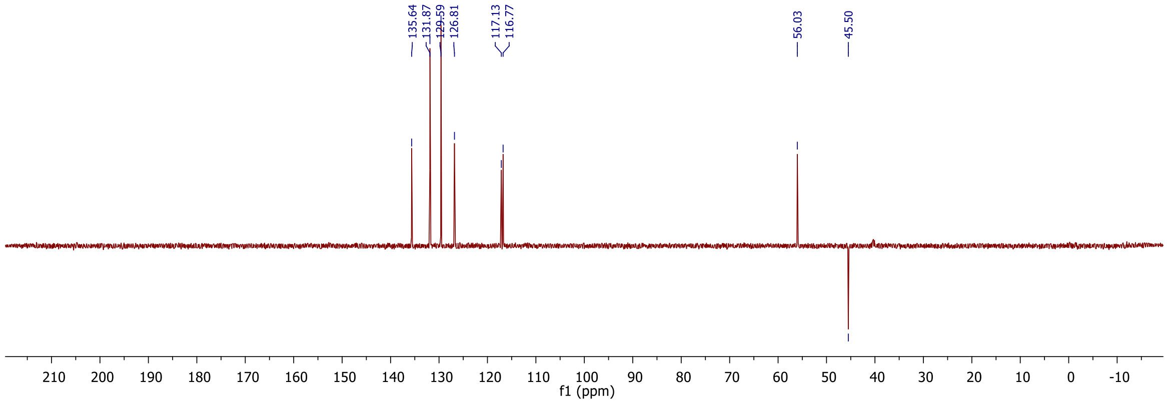
135.64
131.87
129.59
126.81
117.13
116.77
56.03
45.50
210 200 190 180 170 160 150 140 130 120 110 100 90 80 70 60 50 40 30 20 10 0 -10
f1 (ppm)

22b

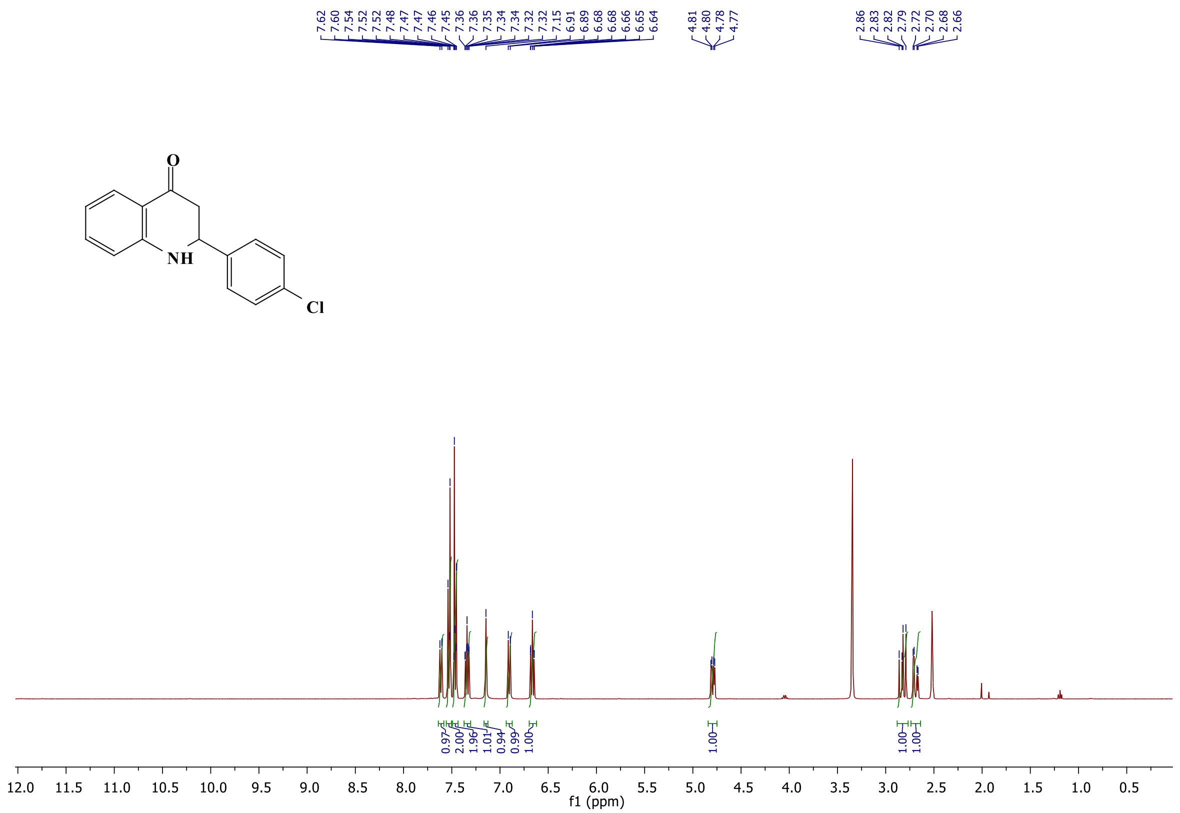
7.62 7.60 7.54 7.52 7.52 7.48 7.47 7.47 7.46 7.45 7.36 7.36 7.35 7.34 7.34 7.32 7.32 7.15 6.91 6.89 6.68 6.68 6.66 6.65 6.64
4.81 4.80 4.78 4.77
2.86 2.83 2.82 2.79 2.72 2.70 2.68 2.66
O
NH
Cl
0.97 2.00 1.96 1.01 0.94 0.99 1.00
1.00
1.00 1.00
f1 (ppm)

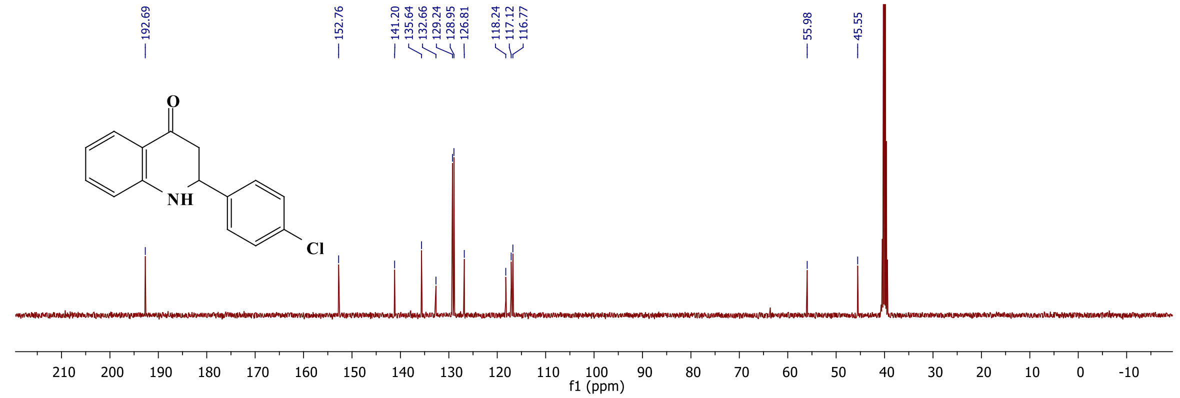
192.69
152.76
141.20 135.64 132.66 129.24 128.95 126.81
118.24 117.12 116.77
55.98
45.55
O
NH
Cl
f1 (ppm)

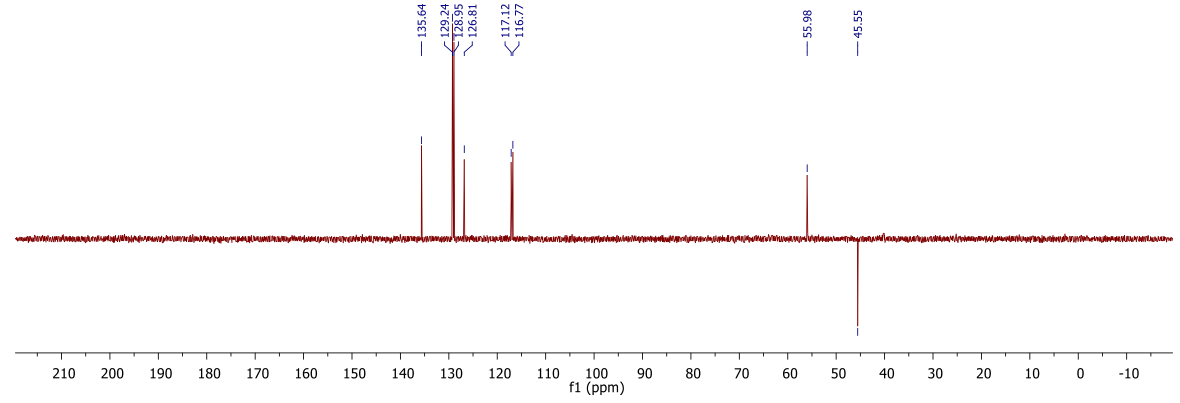
135.64
129.24 128.95 126.81
117.12 116.77
55.98
45.55
f1 (ppm)

22c

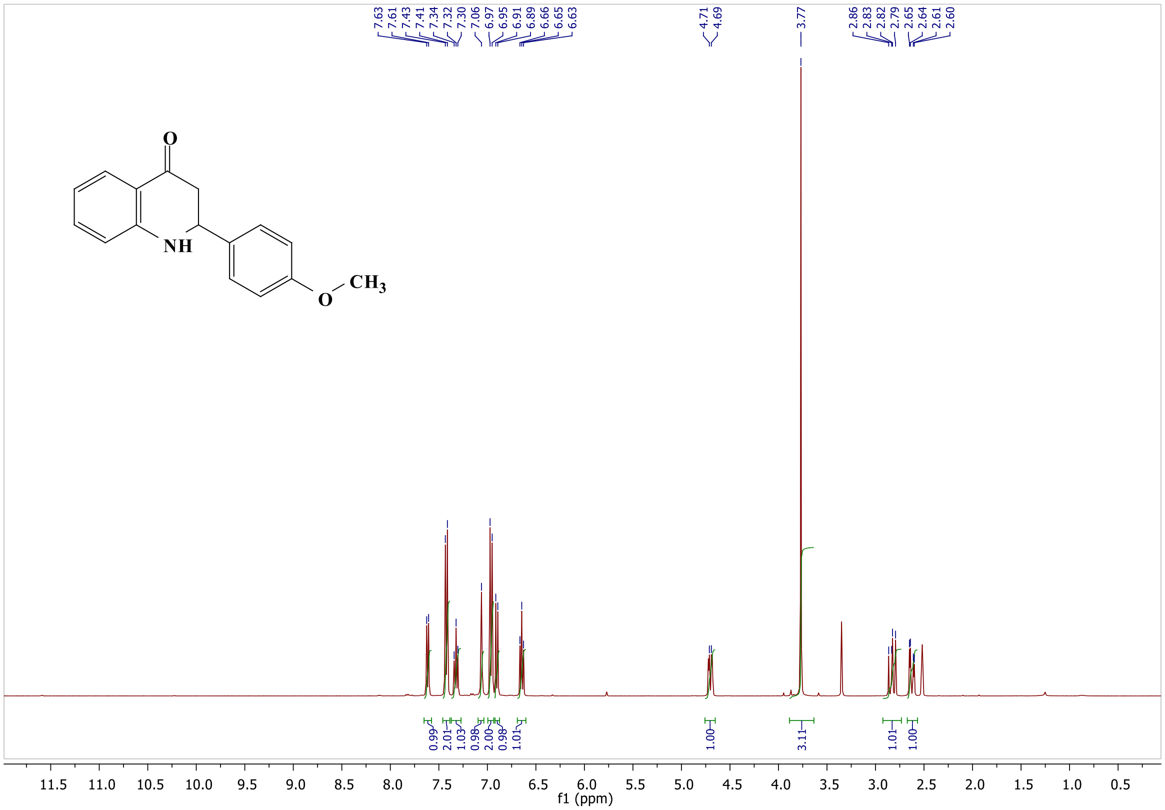
7.63
7.61
7.43
7.41
7.34
7.32
7.30
7.06
6.97
6.95
6.91
6.89
6.66
6.65
6.63
4.71
4.69
3.77
2.86
2.83
2.82
2.79
2.65
2.64
2.61
2.60
O
NH
O
CH3
0.99
2.01
1.03
0.98
2.00
0.98
1.01
1.00
3.11
1.01
1.00
11.5 11.0 10.5 10.0 9.5 9.0 8.5 8.0 7.5 7.0 6.5 6.0 5.5 5.0 4.5 4.0 3.5 3.0 2.5 2.0 1.5 1.0 0.5
f1 (ppm)

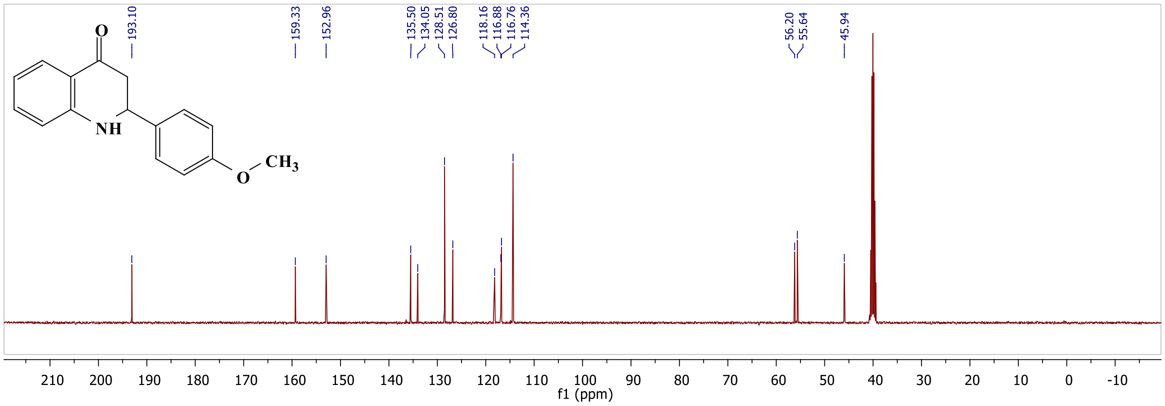
193.10
159.33
152.96
135.50
134.05
128.51
126.80
118.16
116.88
116.76
114.36
56.20
55.64
45.94
O
NH
O
CH3
210 200 190 180 170 160 150 140 130 120 110 100 90 80 70 60 50 40 30 20 10 0 -10
f1 (ppm)

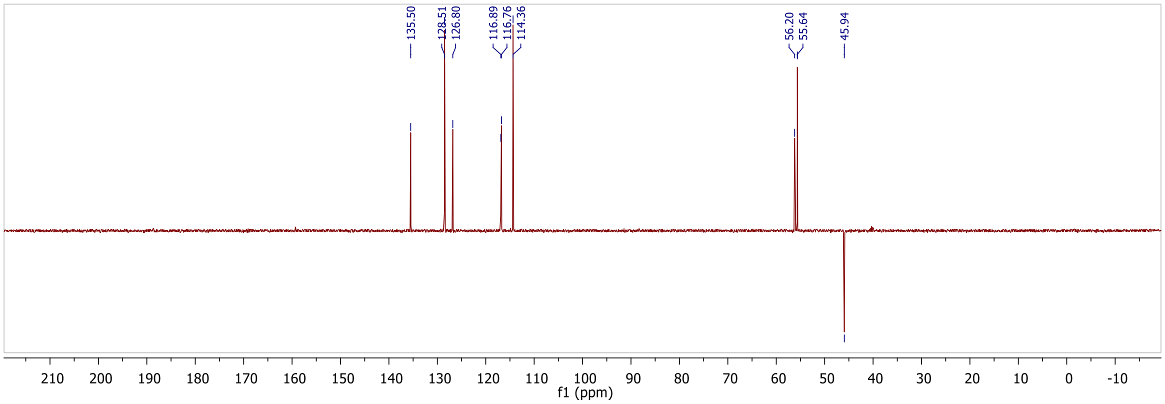
135.50
128.51
126.80
116.89
116.76
114.36
56.20
55.64
45.94
210 200 190 180 170 160 150 140 130 120 110 100 90 80 70 60 50 40 30 20 10 0 -10
f1 (ppm)

22d

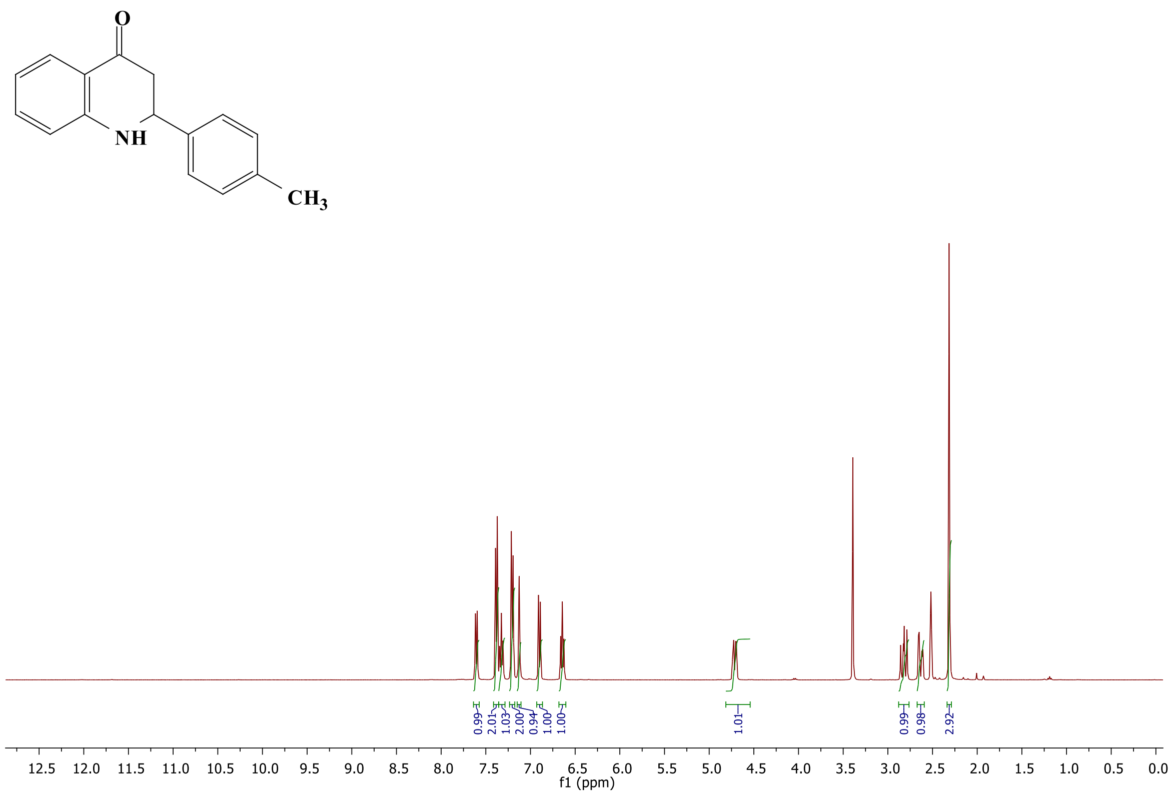
O
NH
CH3
0.99
2.01
1.03
2.00
0.94
1.00
1.00
1.01
0.99
0.98
2.92
12.5 12.0 11.5 11.0 10.5 10.0 9.5 9.0 8.5 8.0 7.5 7.0 6.5 6.0 5.5 5.0 4.5 4.0 3.5 3.0 2.5 2.0 1.5 1.0 0.5 0.0
f1 (ppm)

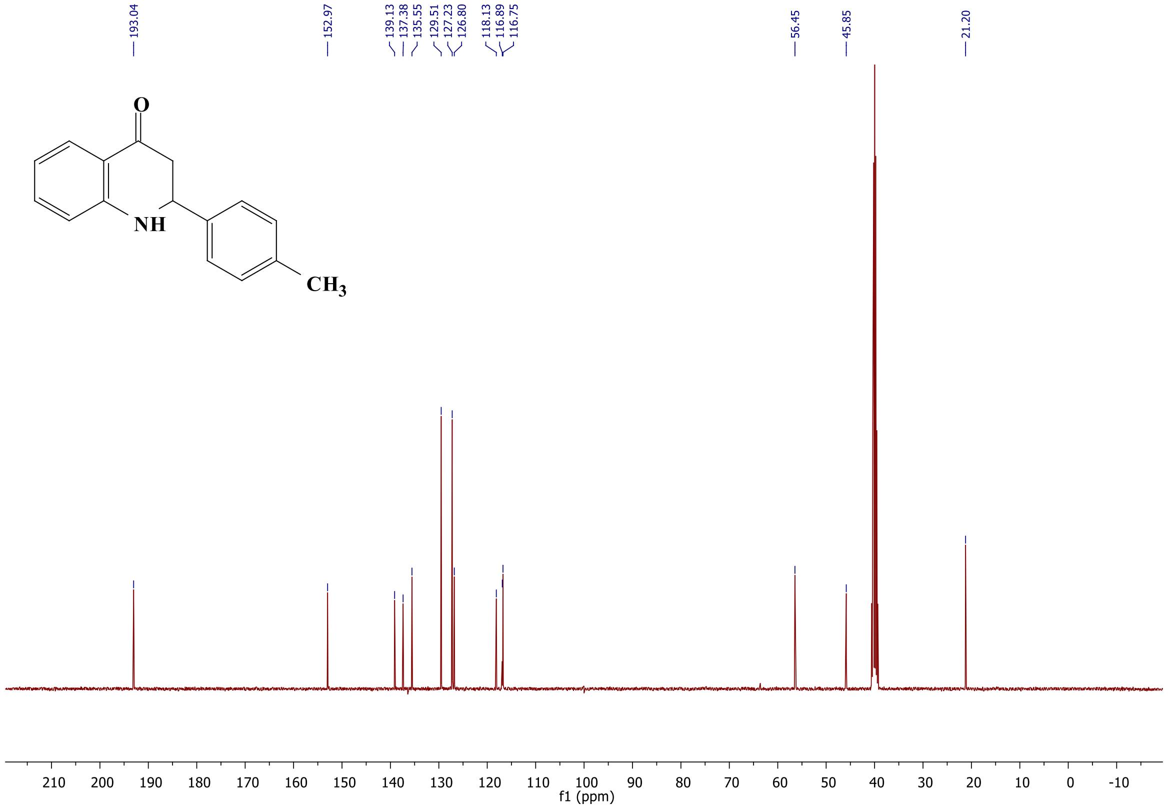
193.04
152.97
139.13
137.38
135.55
129.51
127.23
126.80
118.13
116.89
116.75
56.45
45.85
21.20
O
NH
CH3
210 200 190 180 170 160 150 140 130 120 110 100 90 80 70 60 50 40 30 20 10 0 -10
f1 (ppm)

22e

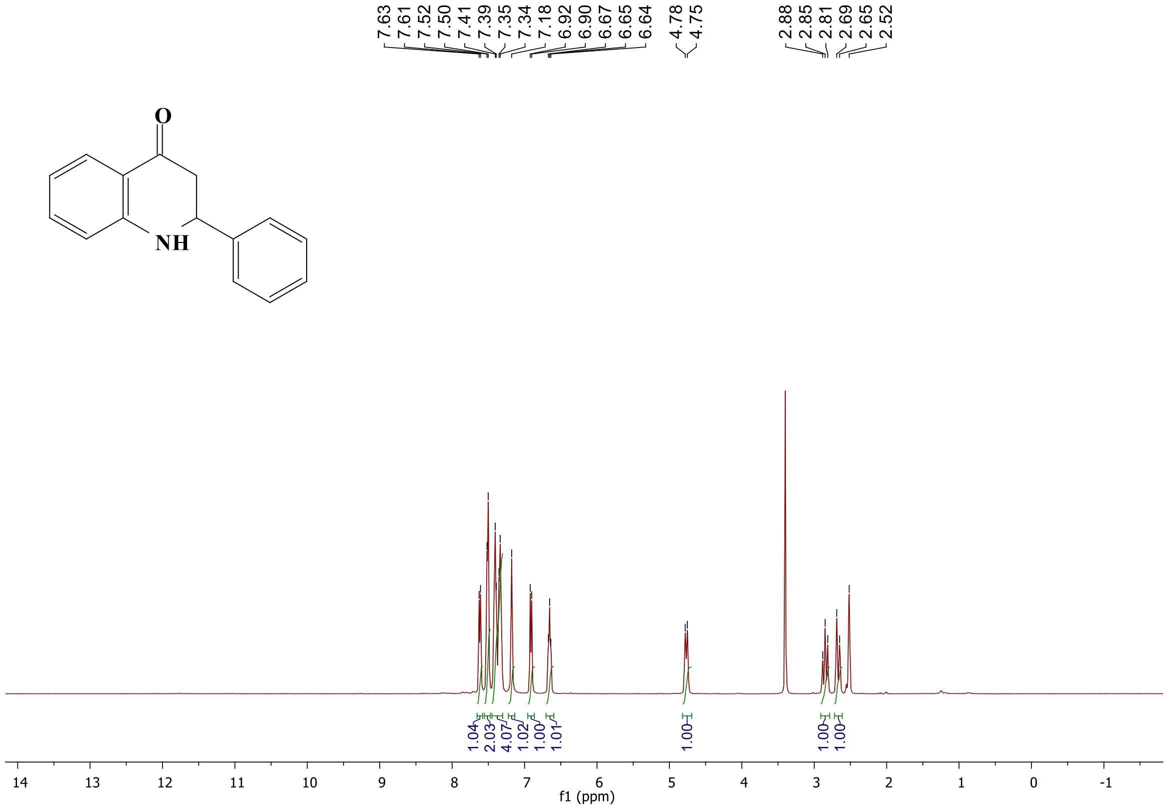
7.63
7.61
7.52
7.50
7.41
7.39
7.35
7.34
7.18
6.92
6.90
6.67
6.65
6.64
4.78
4.75
2.88
2.85
2.81
2.69
2.65
2.52
O
NH
1.04
2.03
4.07
1.02
1.00
1.01
1.00
1.00
1.00
14 13 12 11 10 9 8 7 6 5 4 3 2 1 0 -1
f1 (ppm)

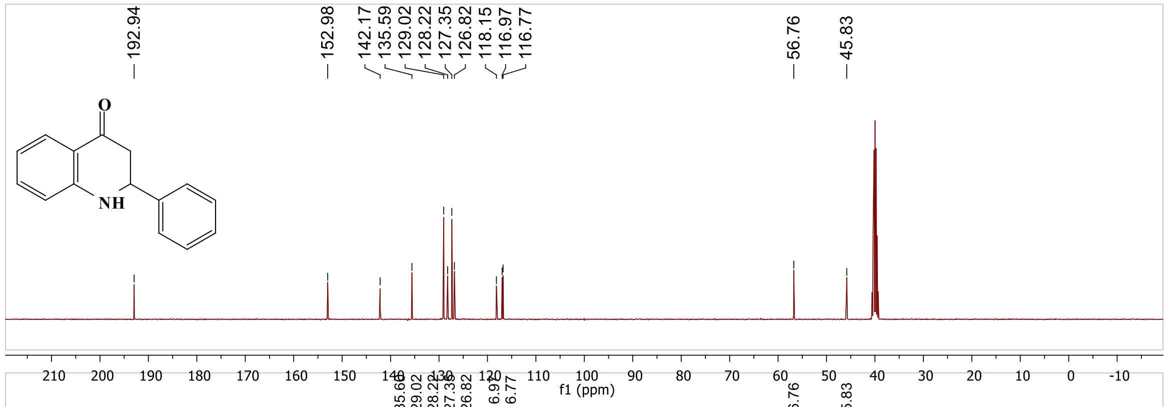
192.94
152.98
142.17
135.59
129.02
128.22
127.35
126.82
118.15
116.97
116.77
56.76
45.83
O
NH
210 200 190 180 170 160 150 140 130 120 110 100 90 80 70 60 50 40 30 20 10 0 -10
f1 (ppm)

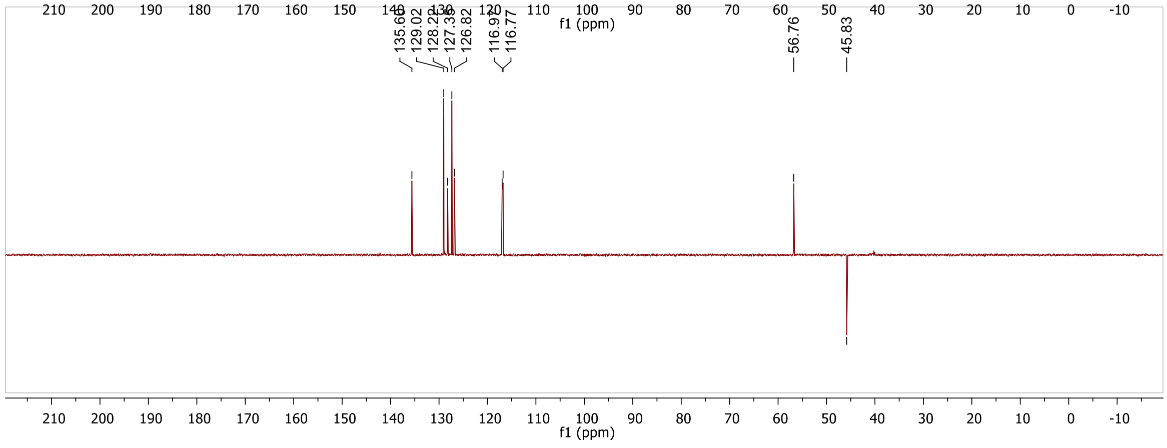
135.60
129.02
128.20
127.35
126.82
116.97
116.77
56.76
45.83
210 200 190 180 170 160 150 140 130 120 110 100 90 80 70 60 50 40 30 20 10 0 -10
f1 (ppm)

22f

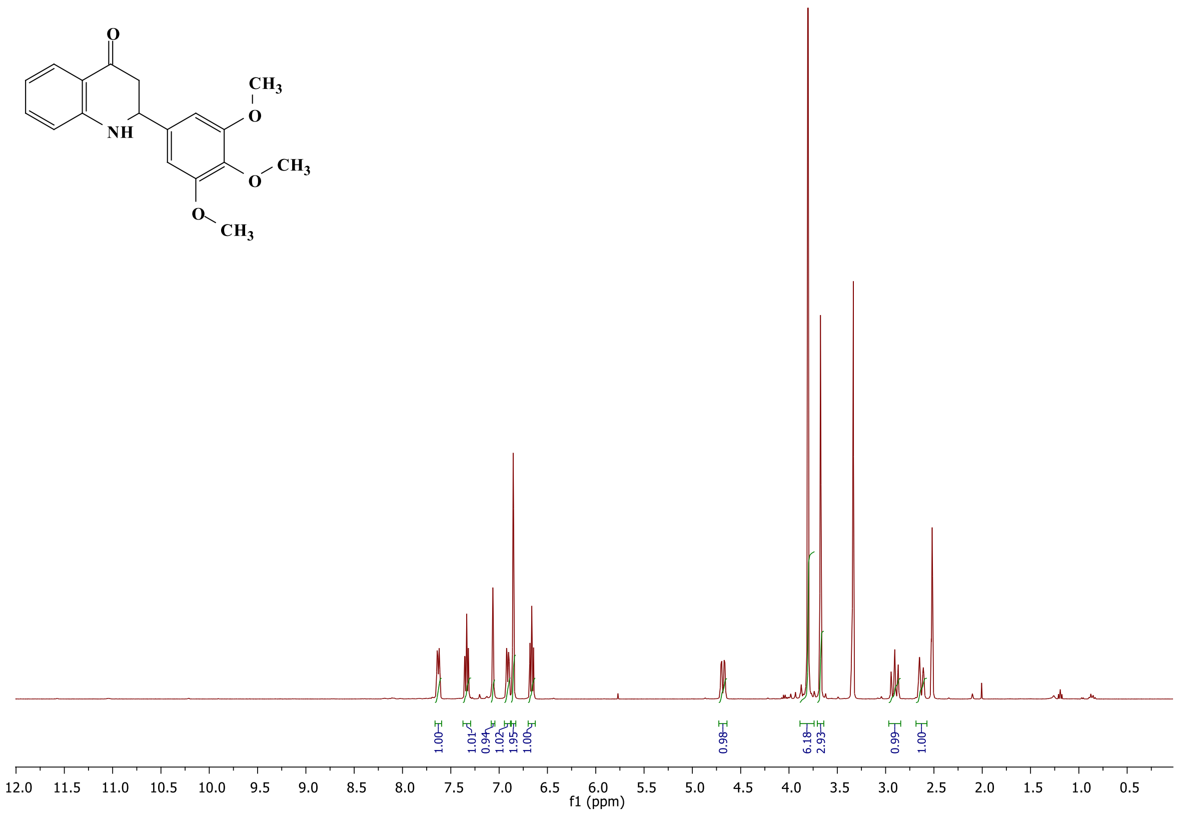
O
CH_3
NH
O
O
CH_3
O
CH_3
1.00
1.01
0.94
1.02
1.95
1.00
0.98
6.18
2.93
0.99
1.00
12.0 11.5 11.0 10.5 10.0 9.5 9.0 8.5 8.0 7.5 7.0 6.5 6.0 5.5 5.0 4.5 4.0 3.5 3.0 2.5 2.0 1.5 1.0 0.5
f1 (ppm)

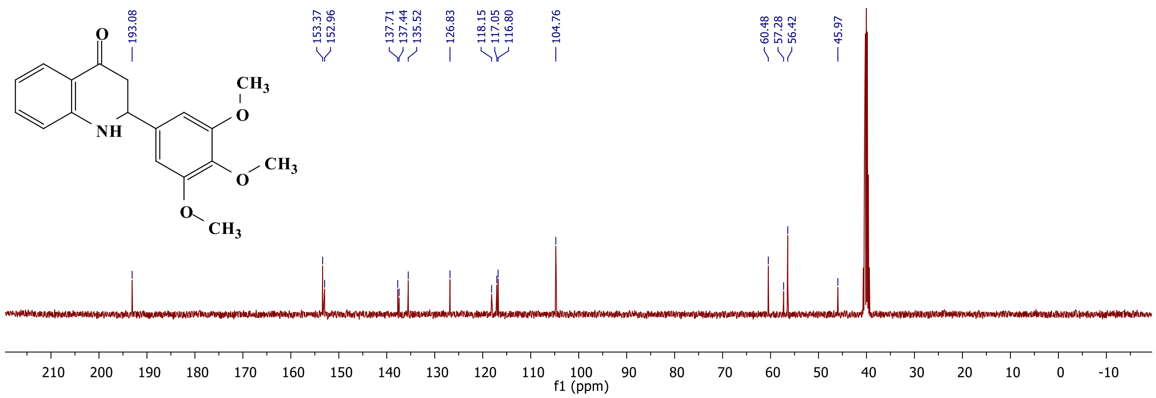
193.08
153.37
152.96
137.71
137.44
135.52
126.83
118.15
117.05
116.80
104.76
60.48
57.28
56.42
45.97
O
CH_3
NH
O
O
CH_3
O
CH_3
210 200 190 180 170 160 150 140 130 120 110 100 90 80 70 60 50 40 30 20 10 0 -10
f1 (ppm)

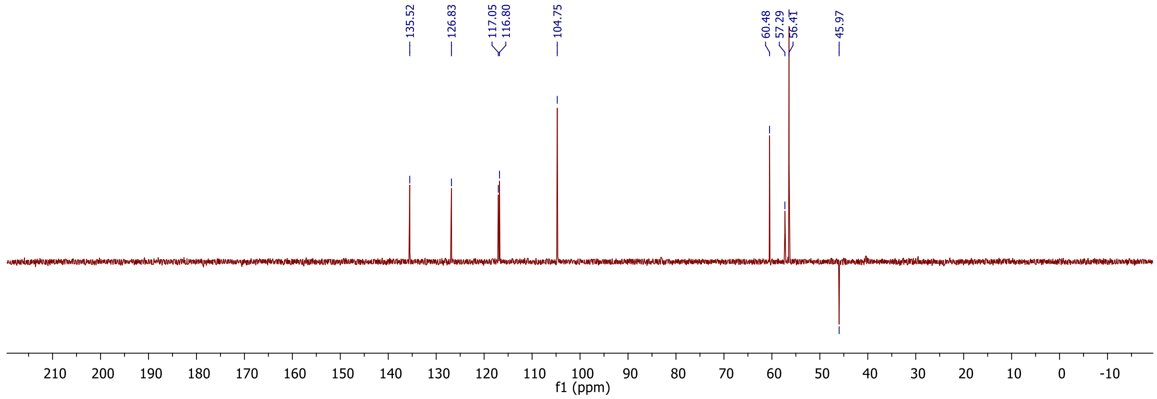
135.52
126.83
117.05
116.80
104.75
60.48
57.29
56.41
45.97
210 200 190 180 170 160 150 140 130 120 110 100 90 80 70 60 50 40 30 20 10 0 -10
f1 (ppm)

22g

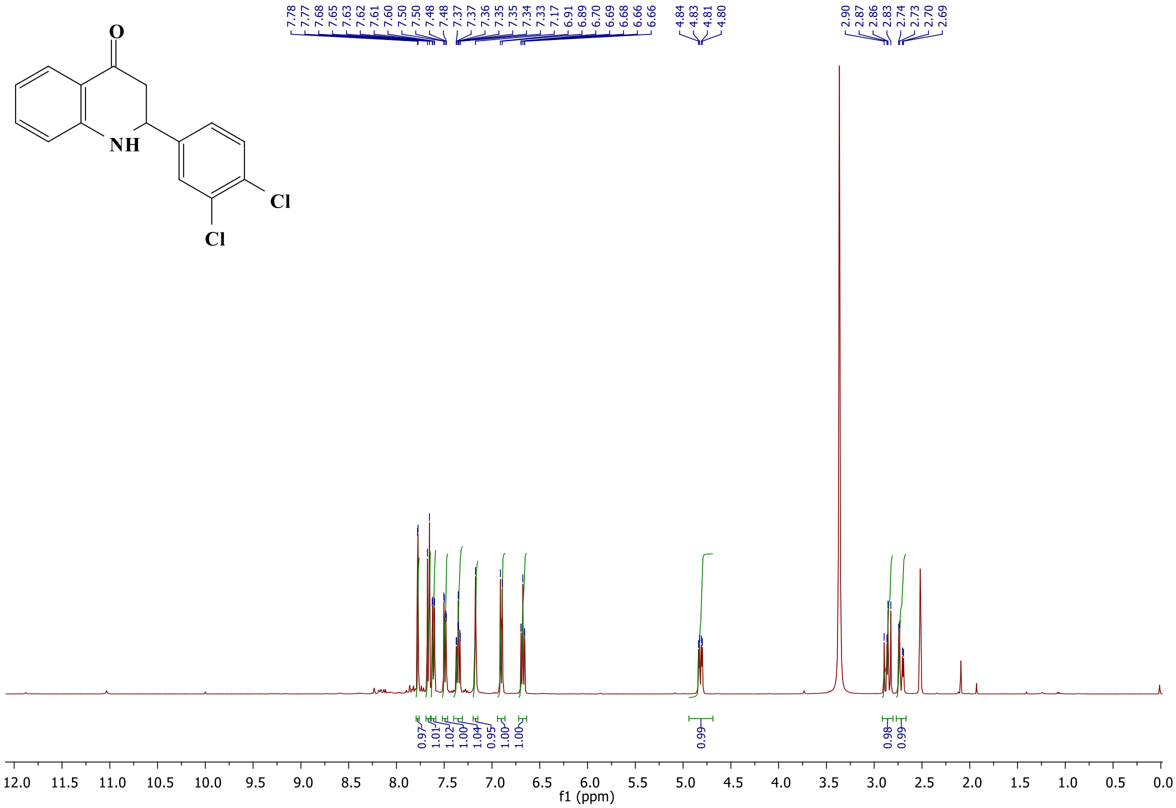

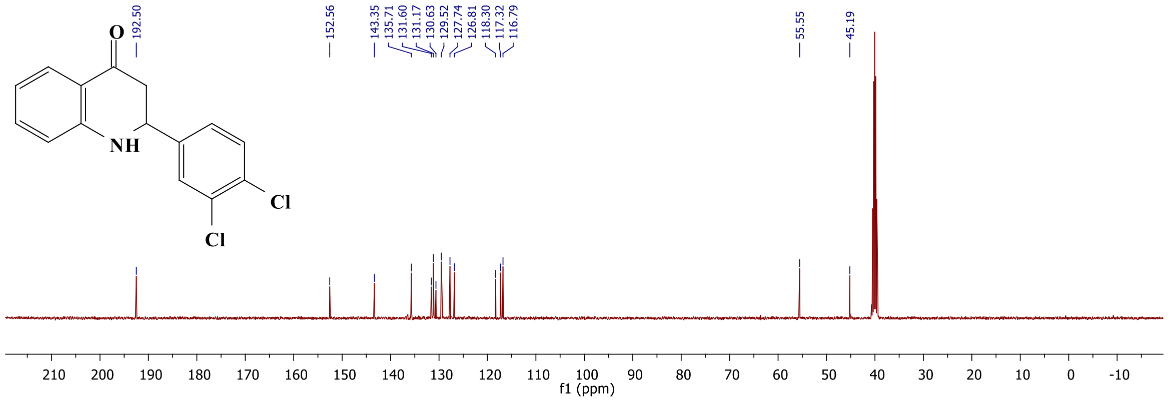

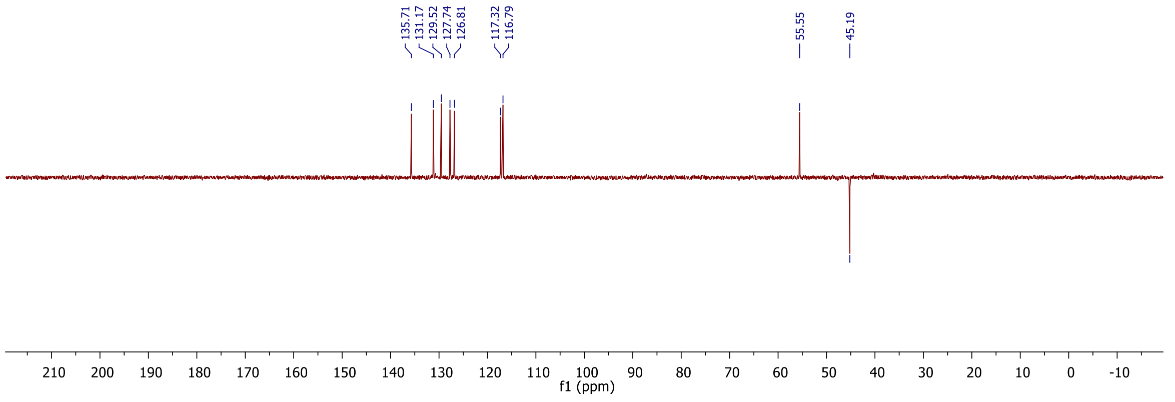

22h

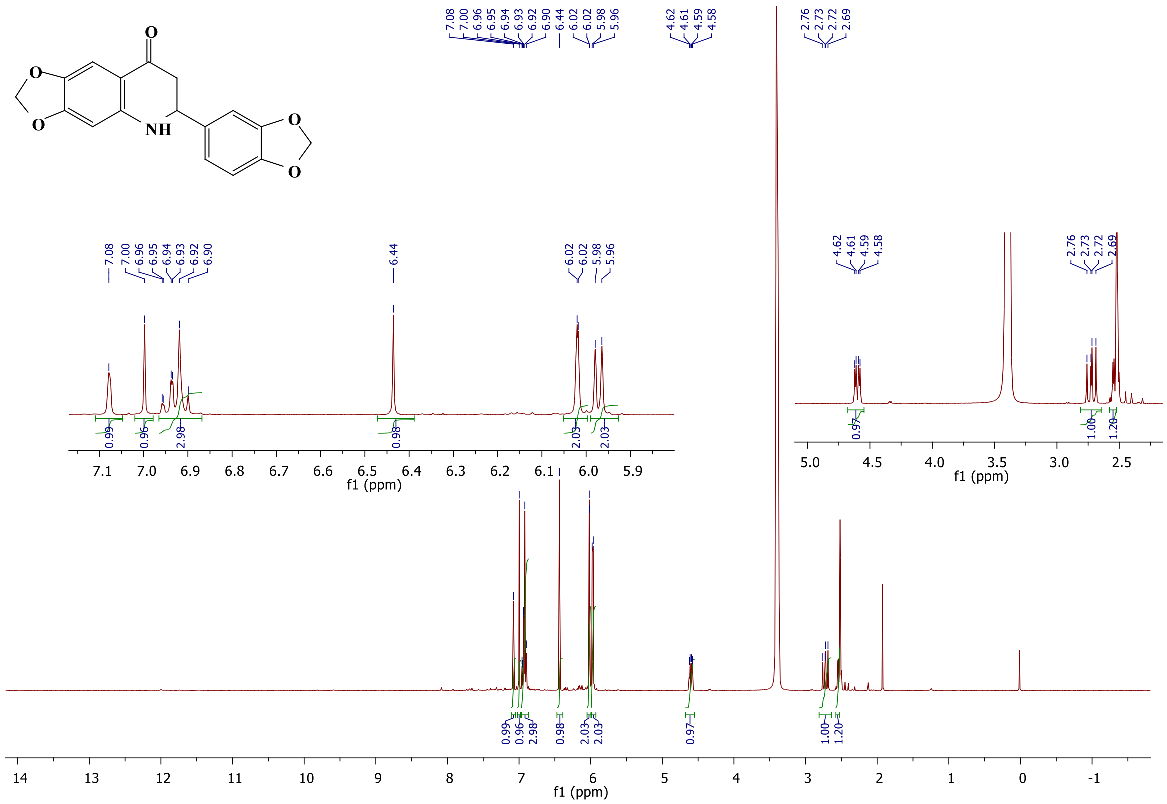

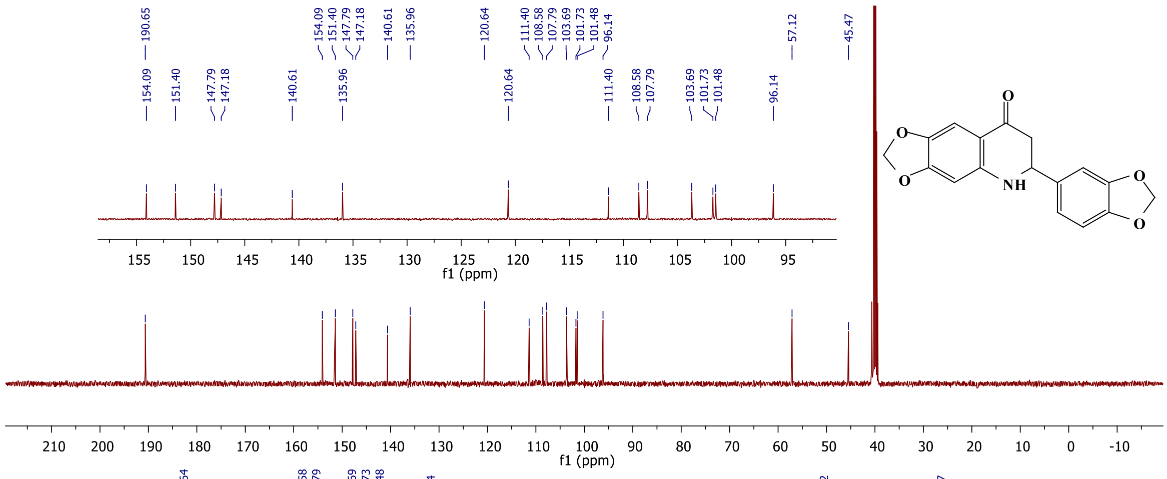

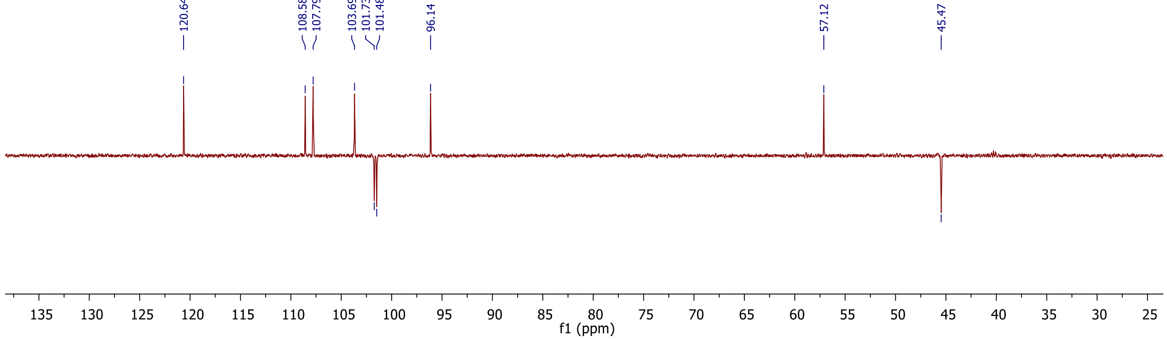

22i

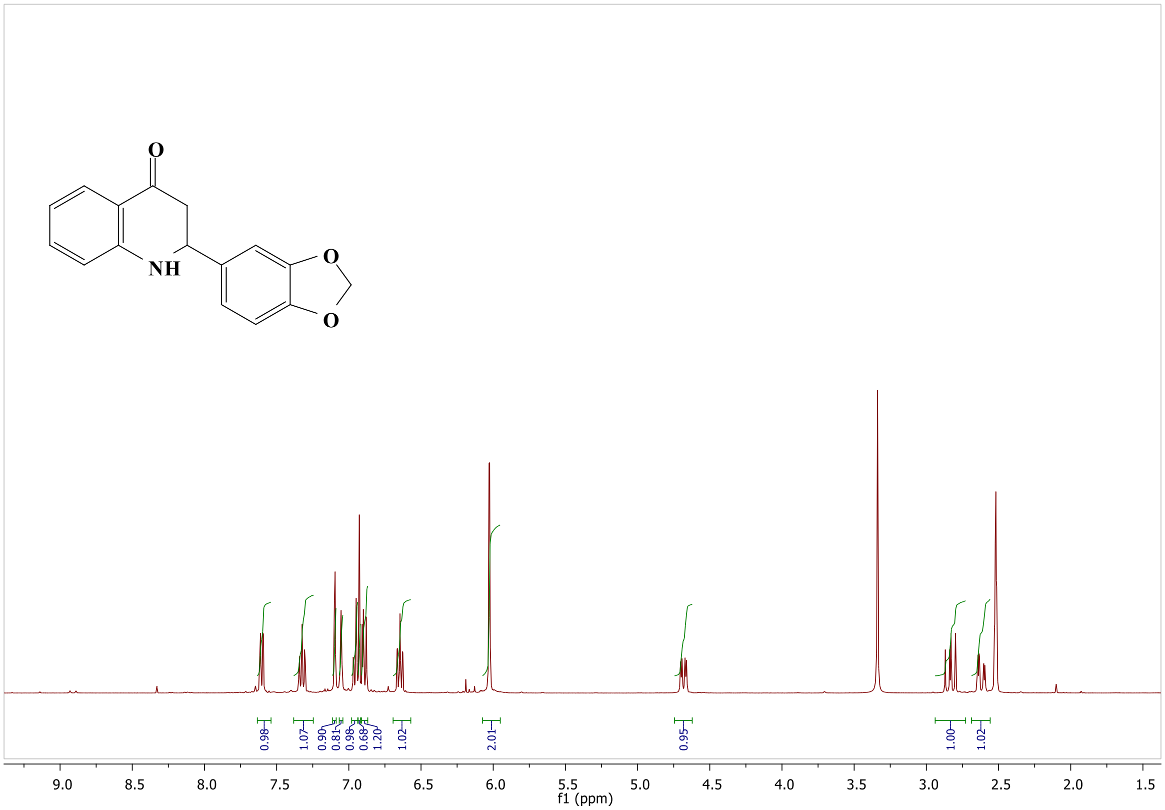
O
NH
O
O
0.98
1.07
0.90
0.81
0.98
0.68
1.20
1.02
2.01
0.95
1.00
1.02
9.0 8.5 8.0 7.5 7.0 6.5 6.0 5.5 5.0 4.5 4.0 3.5 3.0 2.5 2.0 1.5
f1 (ppm)

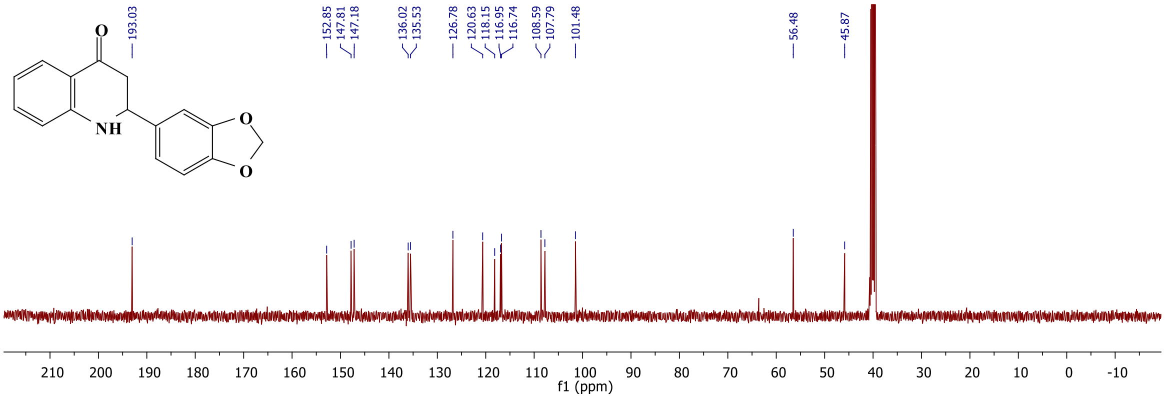
193.03
152.85
147.81
147.18
136.02
135.53
126.78
120.63
118.15
116.95
116.74
108.59
107.79
101.48
56.48
45.87
O
NH
O
O
210 200 190 180 170 160 150 140 130 120 110 100 90 80 70 60 50 40 30 20 10 0 -10
f1 (ppm)

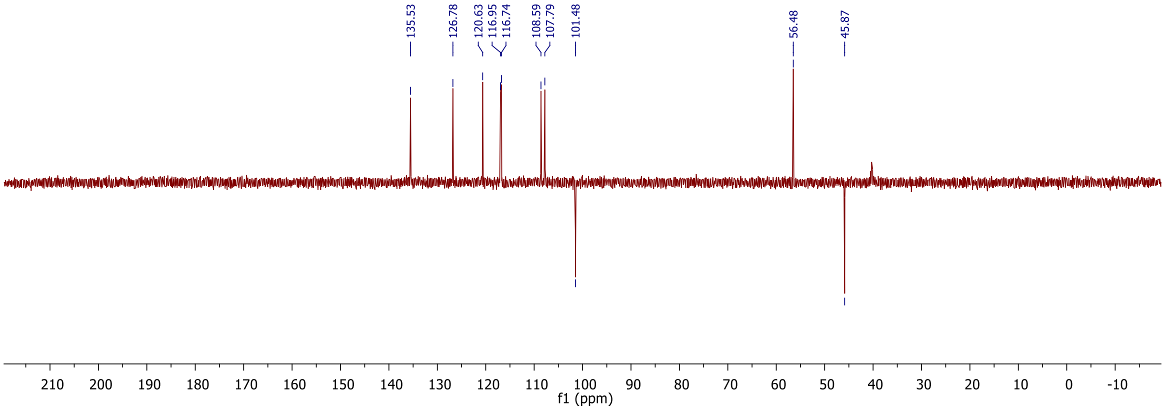
135.53
126.78
120.63
116.95
116.74
108.59
107.79
101.48
56.48
45.87
210 200 190 180 170 160 150 140 130 120 110 100 90 80 70 60 50 40 30 20 10 0 -10
f1 (ppm)

23a

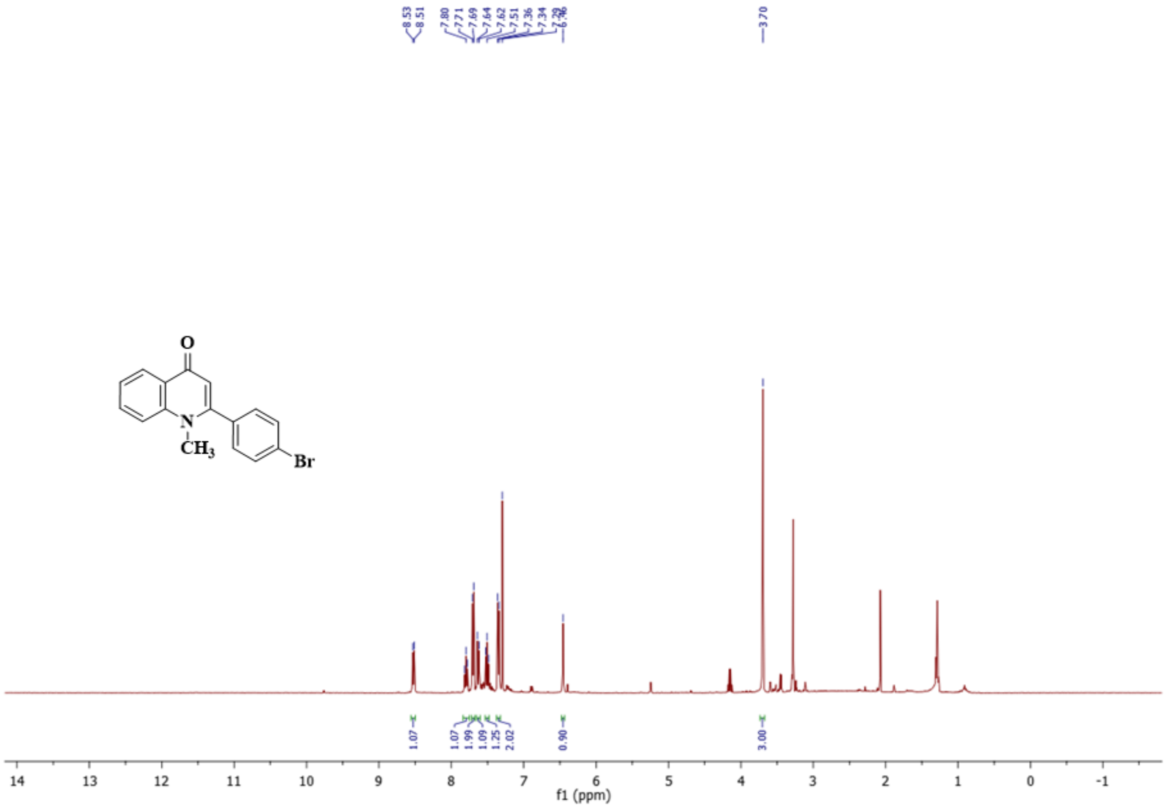

O
N
CH3
Br
8.53
8.51
7.80
7.71
7.69
7.64
7.62
7.51
7.36
7.34
3.70
1.07
1.07
1.99
1.09
1.25
2.02
0.90
3.00
f1 (ppm)

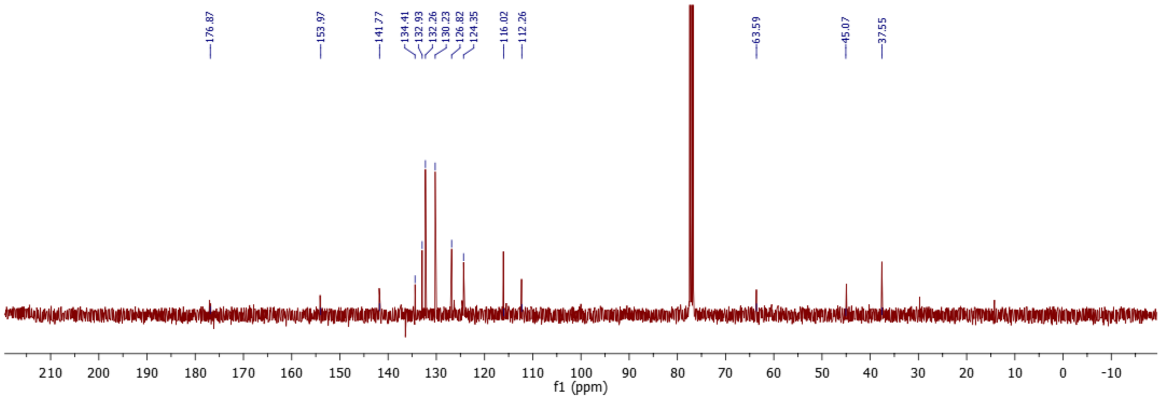

176.87
153.97
141.77
134.41
132.93
132.26
130.23
126.82
124.35
116.02
112.26
63.59
45.07
37.55
f1 (ppm)

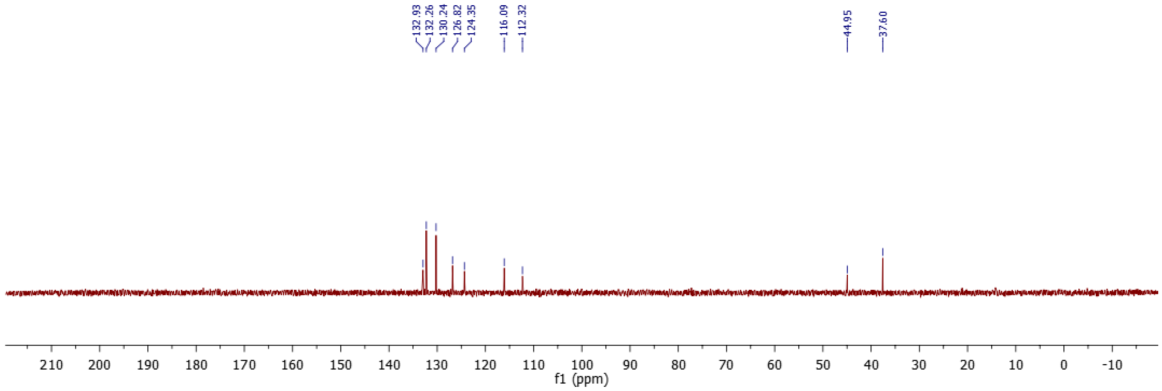

132.93
132.26
130.24
126.82
124.35
116.09
112.32
44.95
37.60
f1 (ppm)

23b

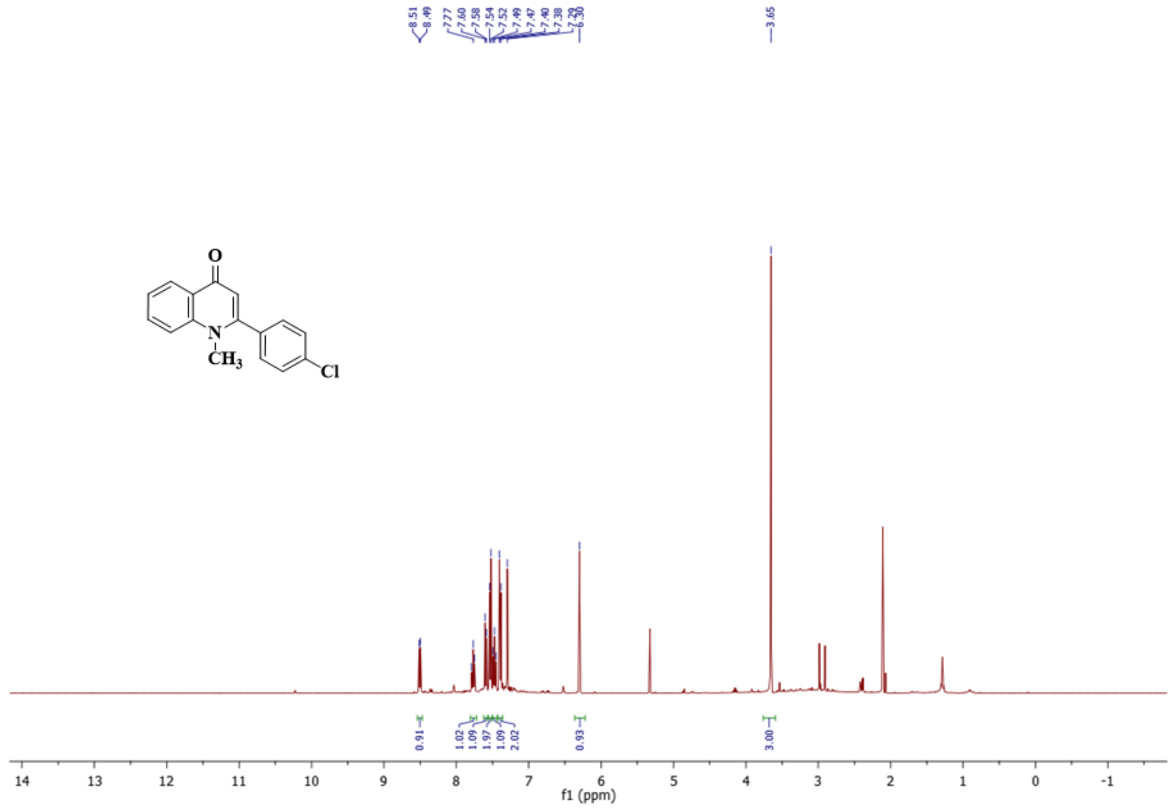
8.51
8.49
7.77
7.60
7.58
7.54
7.52
7.49
7.47
7.40
7.38
6.30
3.65
O
N
CH3
Cl
0.91
1.02
1.09
1.97
1.09
2.02
0.93
3.00
f1 (ppm)

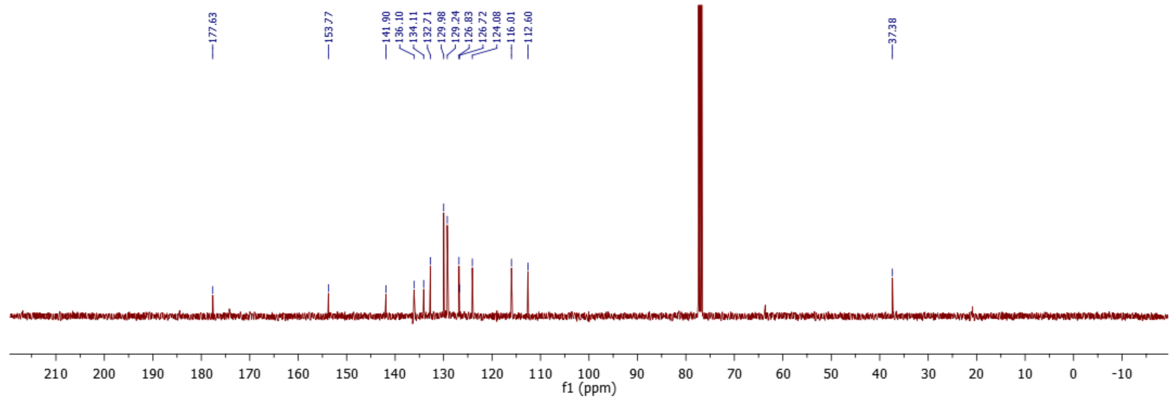
177.63
153.77
141.90
136.10
134.11
132.71
129.98
129.24
126.83
126.72
124.08
116.01
112.60
37.38
f1 (ppm)

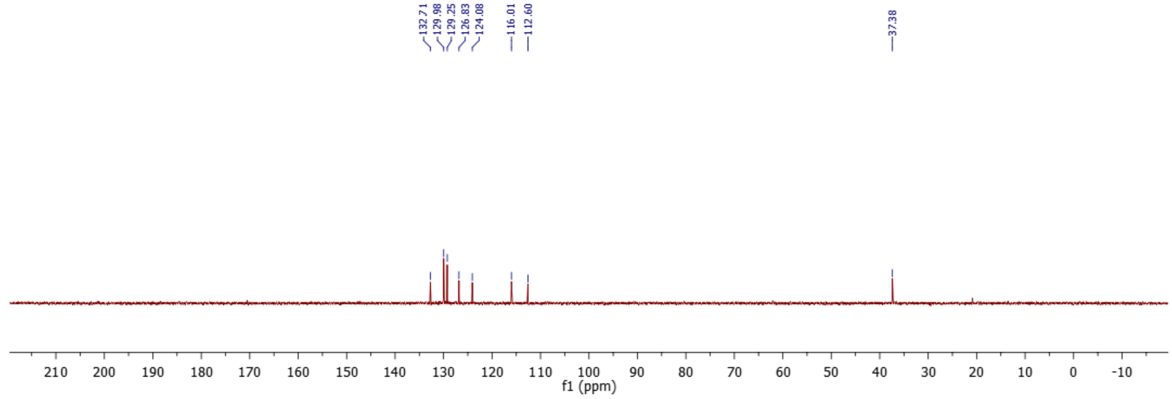
132.71
129.98
129.25
126.83
124.08
116.01
112.60
37.38
f1 (ppm)

23c

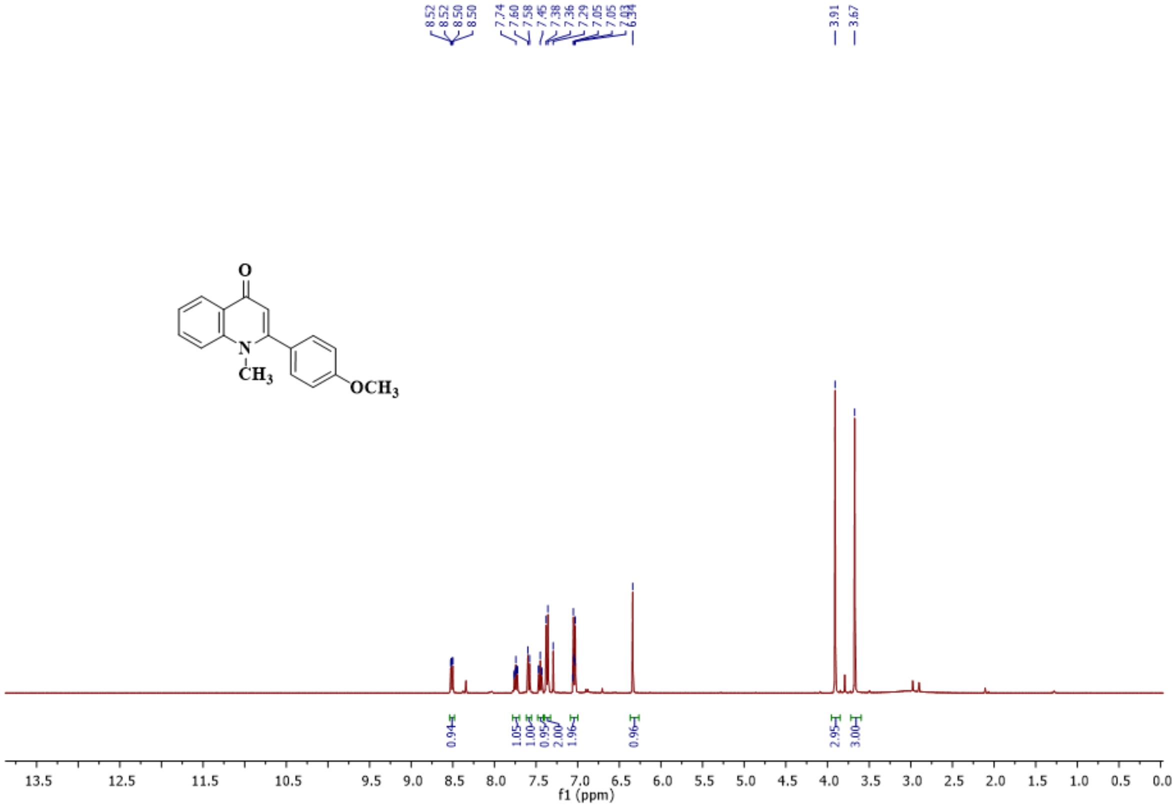
O
N
CH3
OCH3
8.52
8.52
8.50
8.50
7.74
7.60
7.58
7.45
7.38
7.36
7.29
7.05
7.05
3.91
3.67
0.94
1.05
1.00
0.95
2.00
1.96
0.96
2.95
3.00
f1 (ppm)

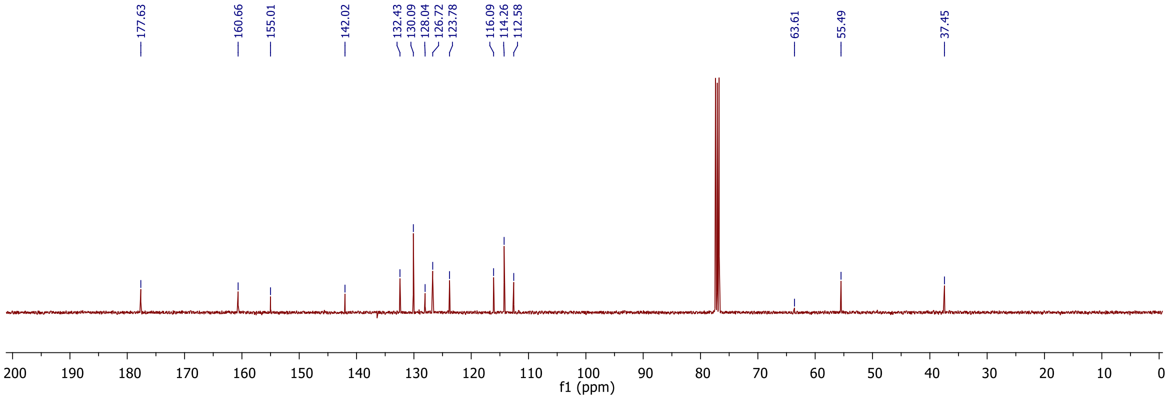
177.63
160.66
155.01
142.02
132.43
130.09
128.04
126.72
123.78
116.09
114.26
112.58
63.61
55.49
37.45
f1 (ppm)

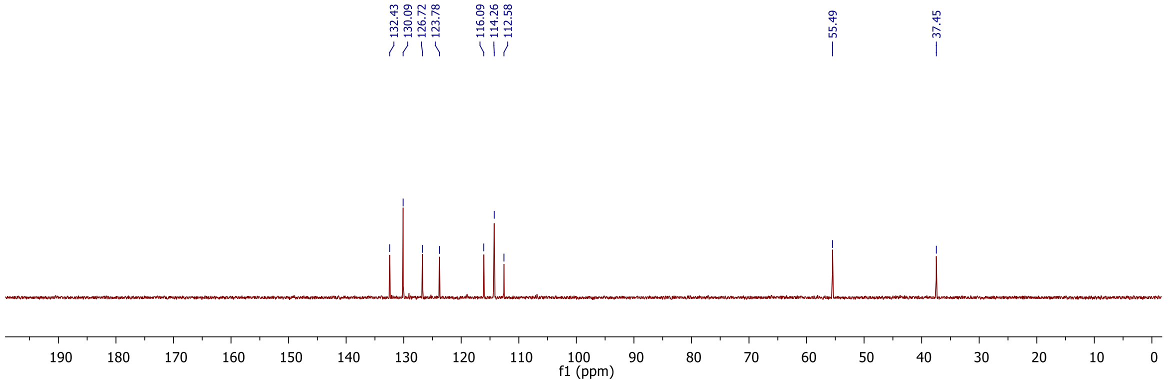
132.43
130.09
126.72
123.78
116.09
114.26
112.58
55.49
37.45
f1 (ppm)

23d

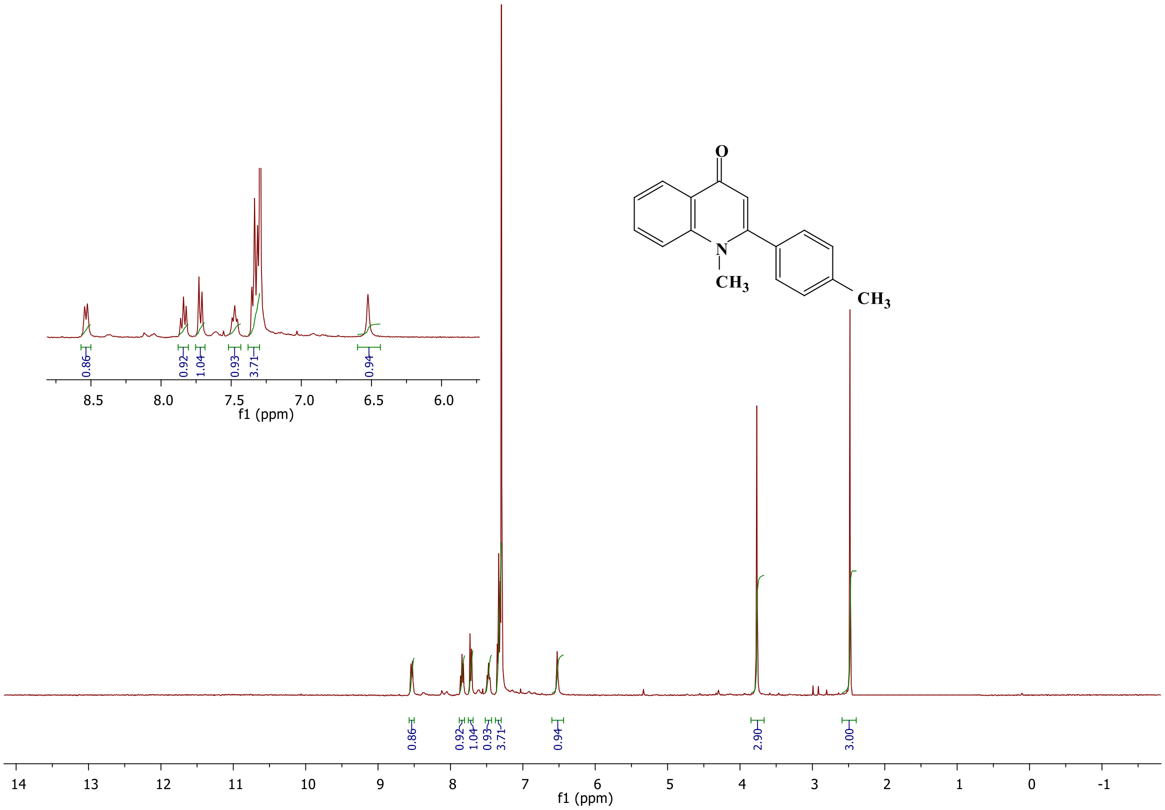
O
N
CH3
CH3
0.86
0.92
1.04
0.93
3.71
0.94
2.90
3.00
f1 (ppm)

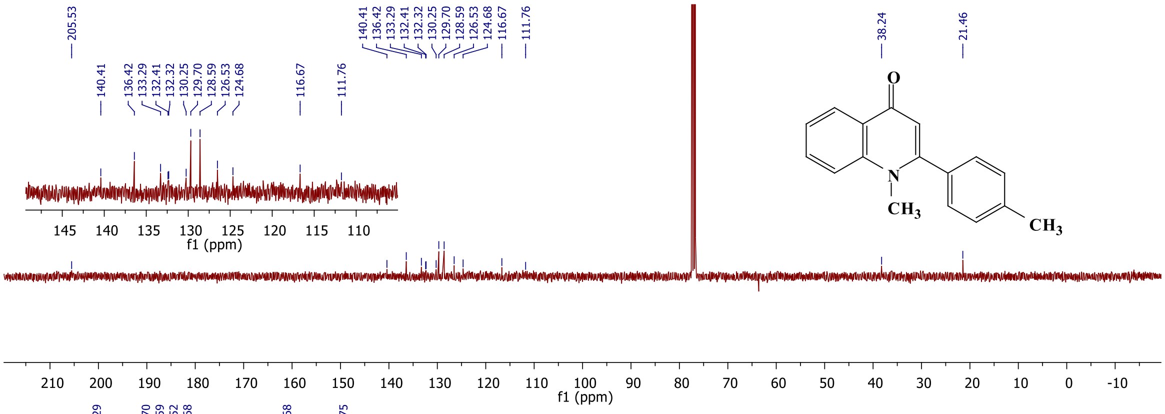
205.53
140.41
136.42
133.29
132.41
132.32
130.25
129.70
128.59
126.53
124.68
116.67
111.76
38.24
21.46
O
N
CH3
CH3
f1 (ppm)

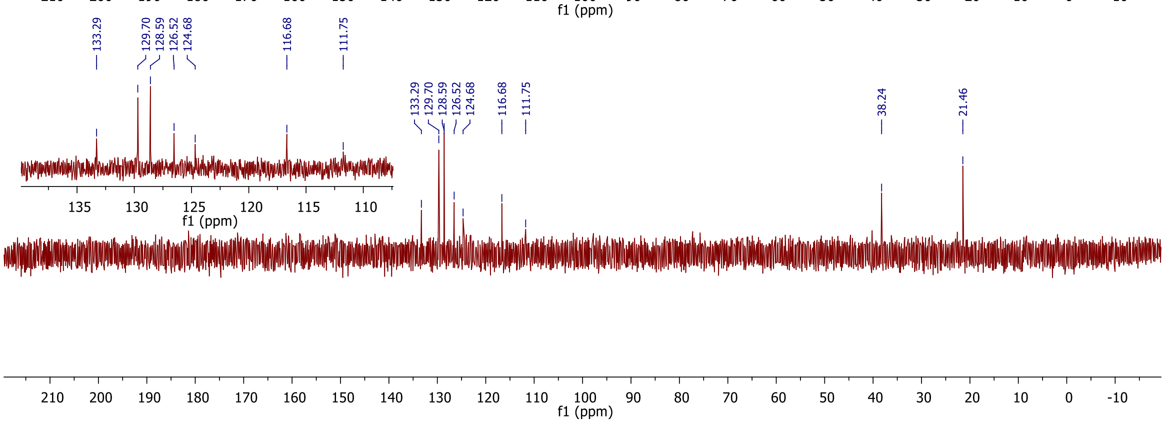
133.29
129.70
128.59
126.52
124.68
116.68
111.75
38.24
21.46
f1 (ppm)

23e

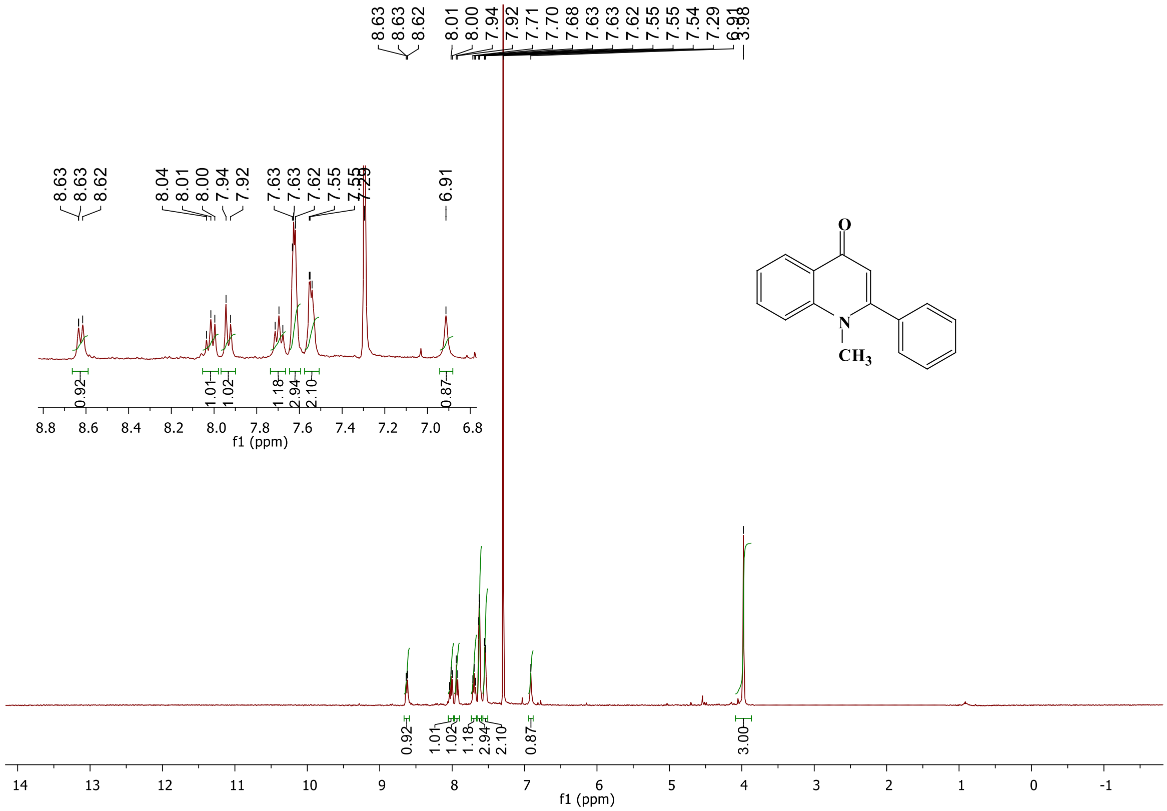
8.63
8.63
8.62
8.01
8.00
7.94
7.92
7.71
7.70
7.68
7.63
7.63
7.62
7.55
7.55
7.54
7.29
6.91
3.98
8.04
0.92
1.01
1.02
1.18
2.94
2.10
0.87
3.00
f1 (ppm)

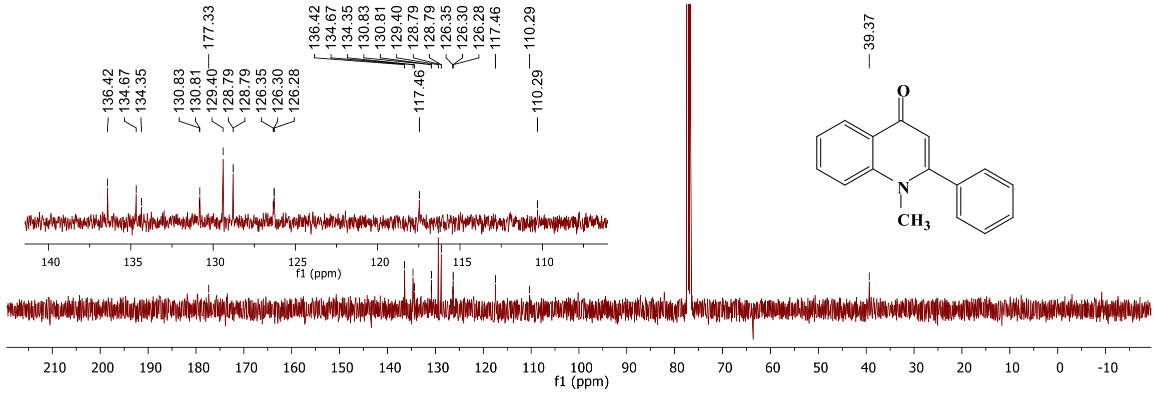
136.42
134.67
134.35
130.83
130.81
129.40
128.79
128.79
126.35
126.30
126.28
117.46
110.29
177.33
39.37
f1 (ppm)

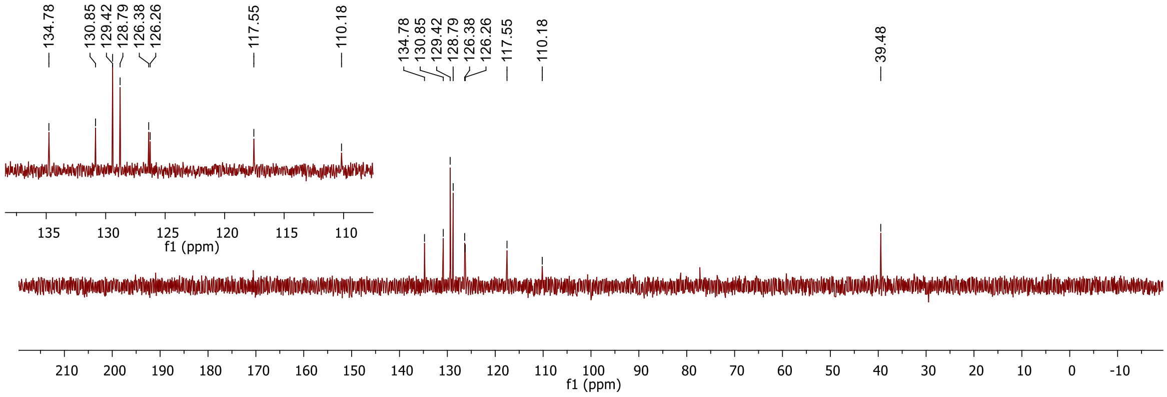
134.78
130.85
129.42
128.79
126.38
126.26
117.55
110.18
39.48
f1 (ppm)

23f

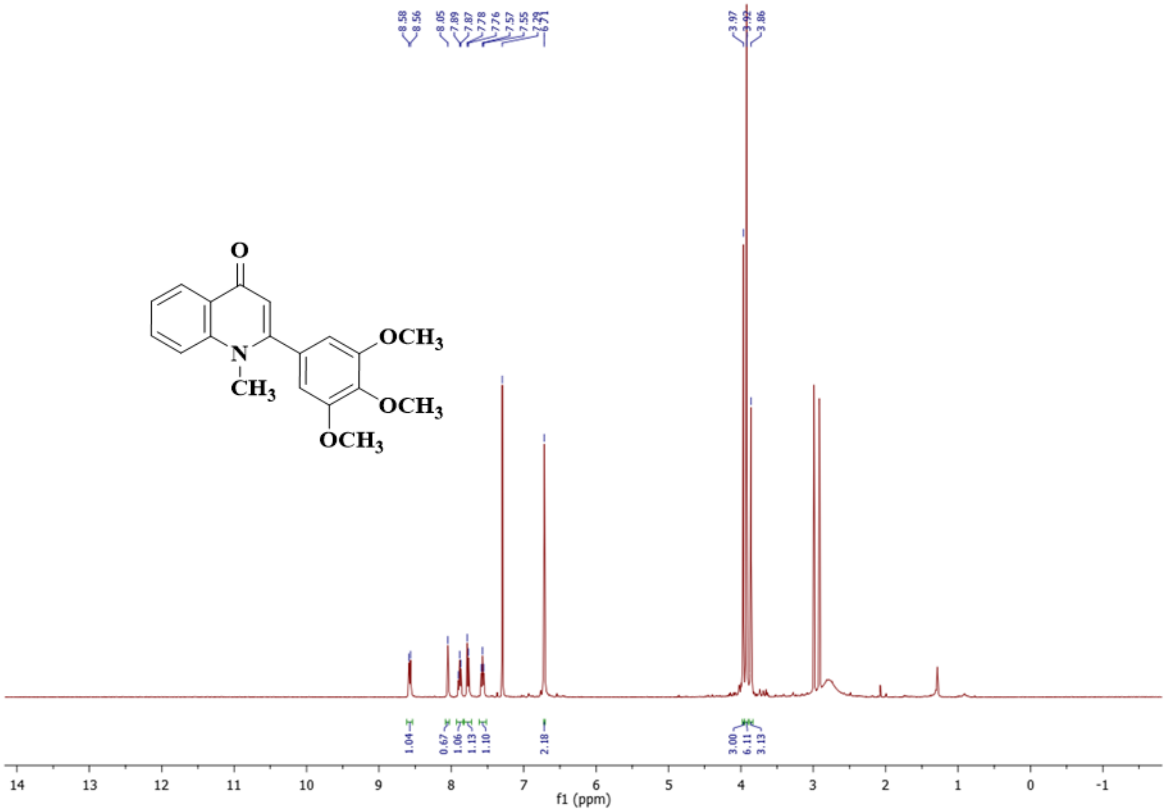

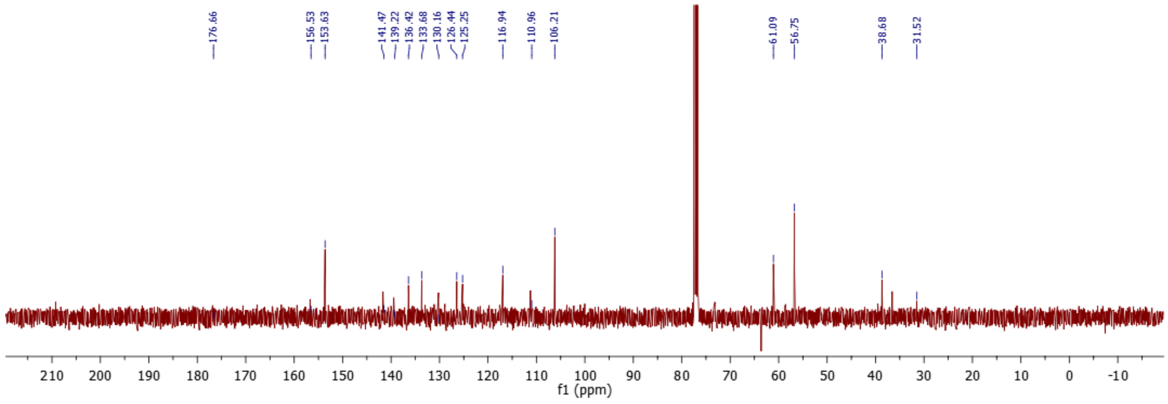

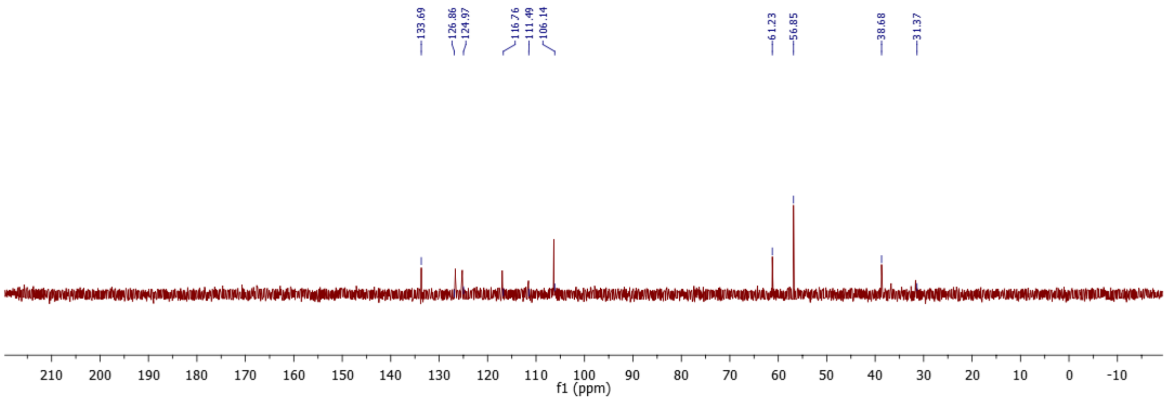

23g

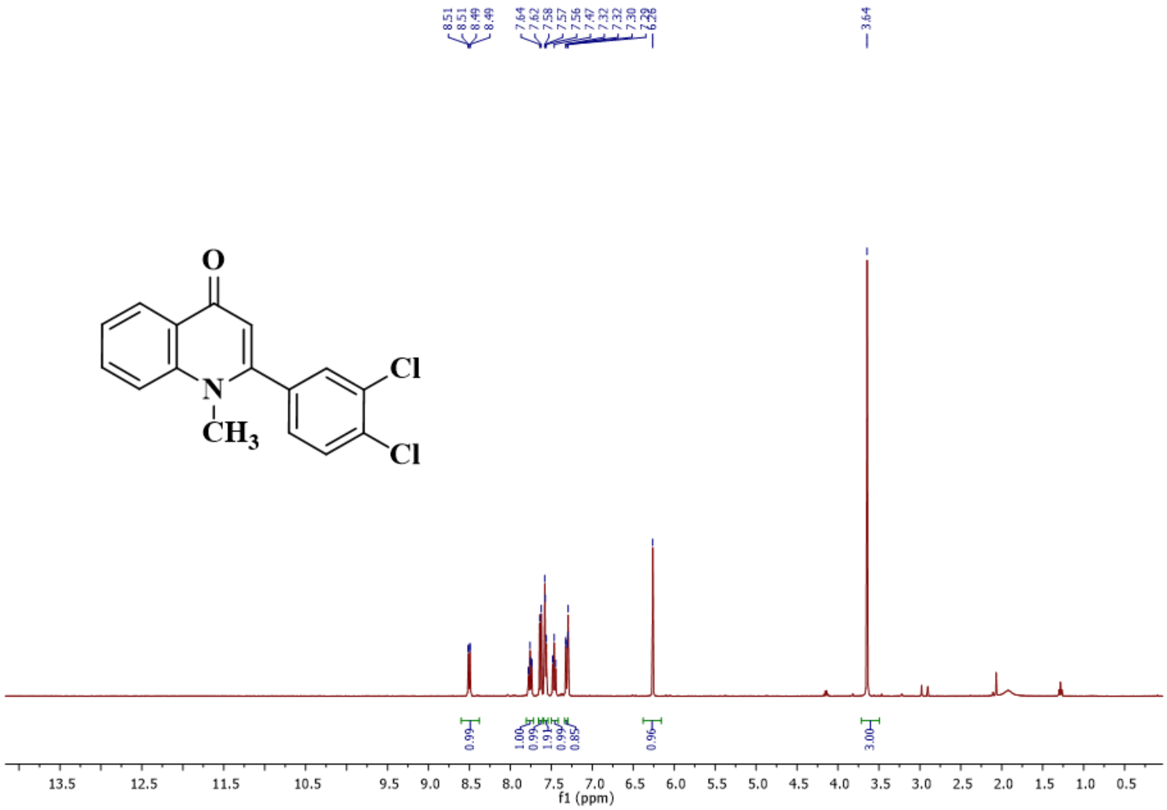

O
N
CH3
Cl
Cl
8.51
8.51
8.49
8.49
7.64
7.62
7.58
7.57
7.56
7.47
7.32
7.32
7.30
6.28
3.64
0.99
1.00
0.99
1.91
0.99
0.85
0.96
3.00
f1 (ppm)

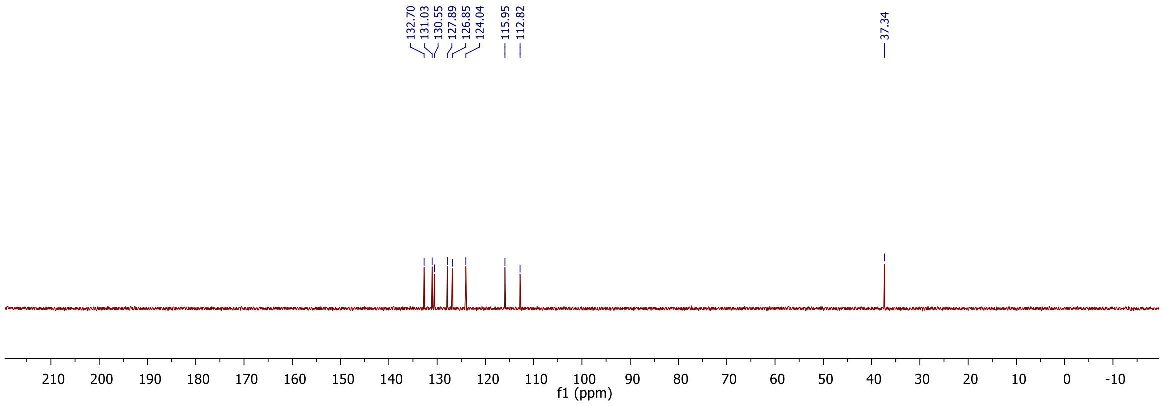

132.70
131.03
130.55
127.89
126.85
124.04
115.95
112.82
37.34
f1 (ppm)

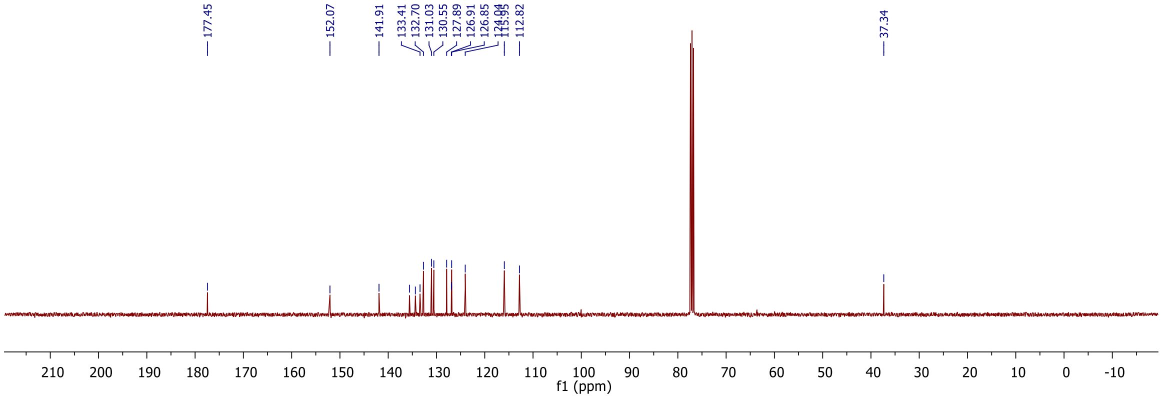

177.45
152.07
141.91
133.41
132.70
131.03
130.55
127.89
126.91
126.85
112.82
37.34
f1 (ppm)

23h

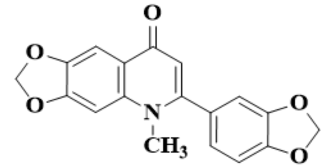

O
O
O
N
CH3
O
O

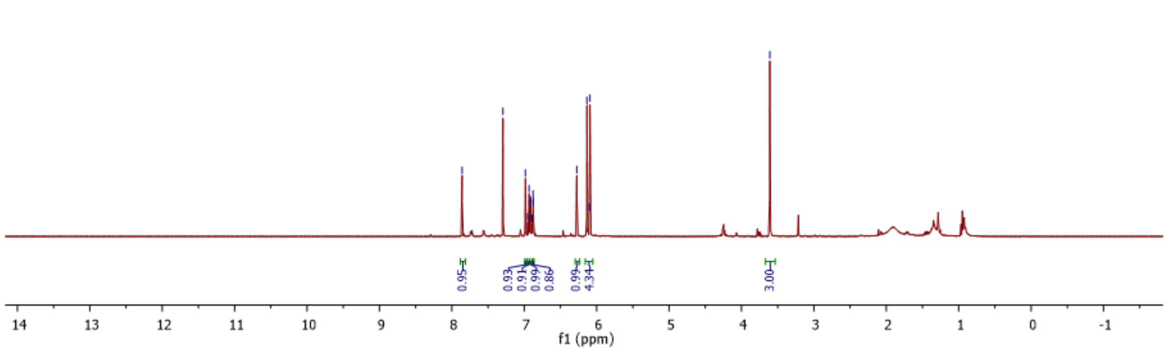

7.86
7.29
6.98
6.93
6.91
6.88
6.87
6.23
6.13
6.10
6.09
3.61
0.95
0.93
0.91
0.99
0.86
0.99
4.34
3.00
f1 (ppm)

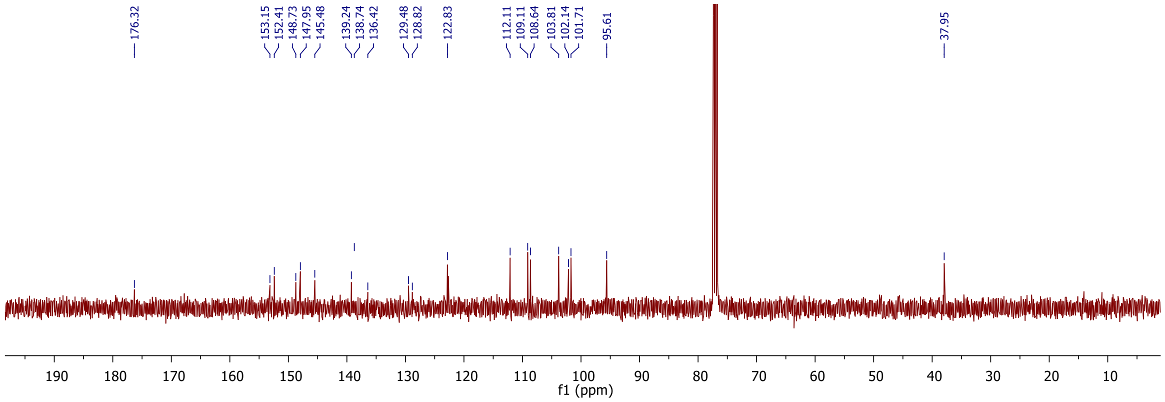

176.32
153.15
152.41
148.73
147.95
145.48
139.24
138.74
136.42
129.48
128.82
122.83
112.11
109.11
108.64
103.81
102.14
101.71
95.61
37.95
f1 (ppm)

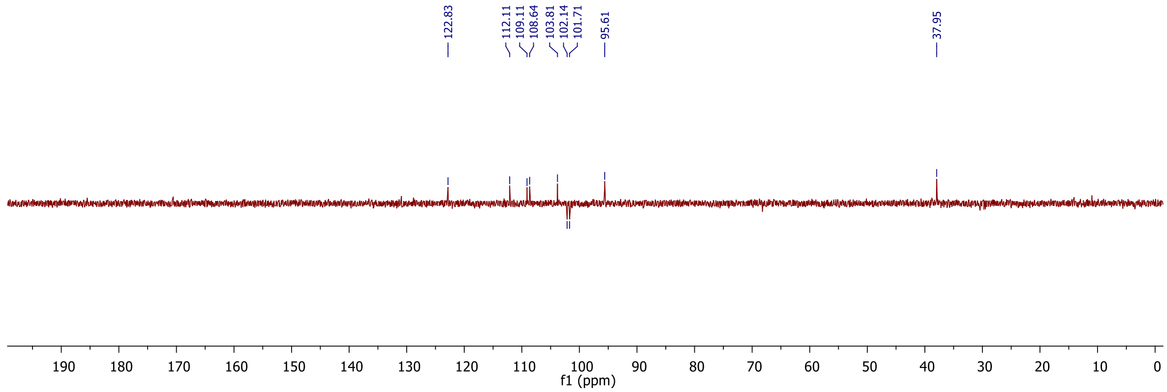

122.83
112.11
109.11
108.64
103.81
102.14
101.71
95.61
37.95
f1 (ppm)

Graveoline 1

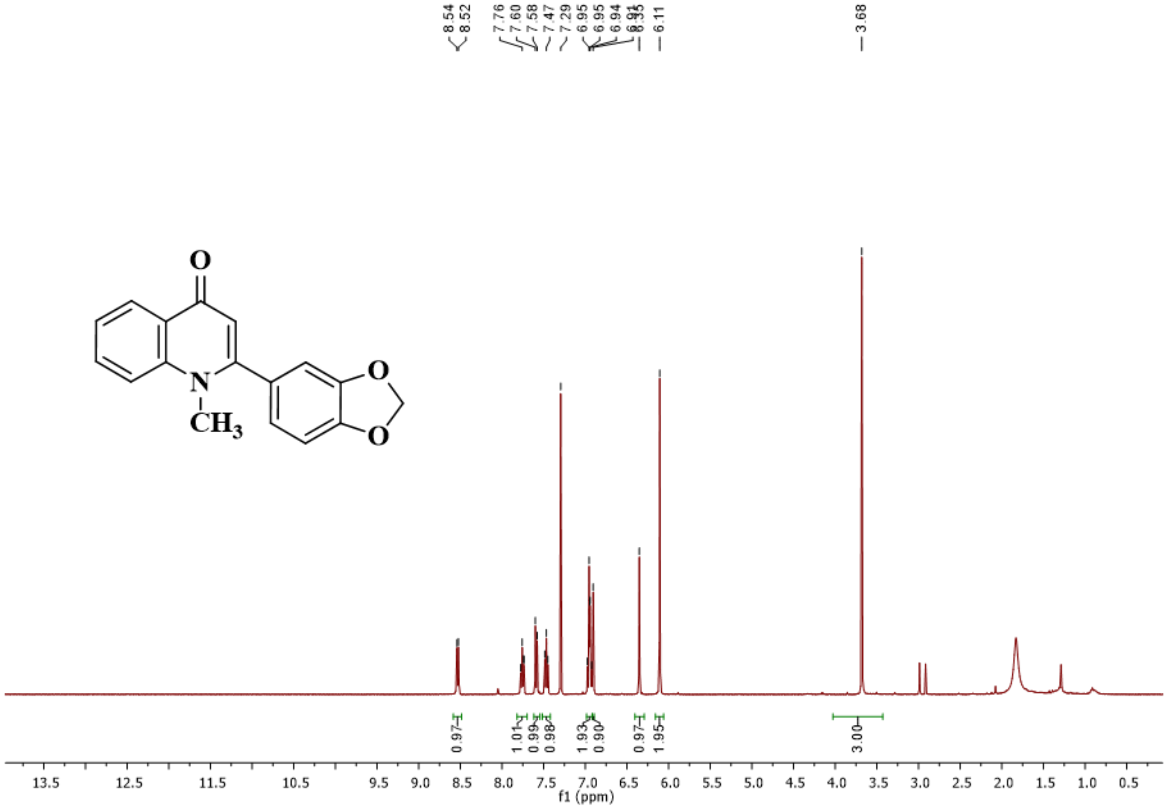

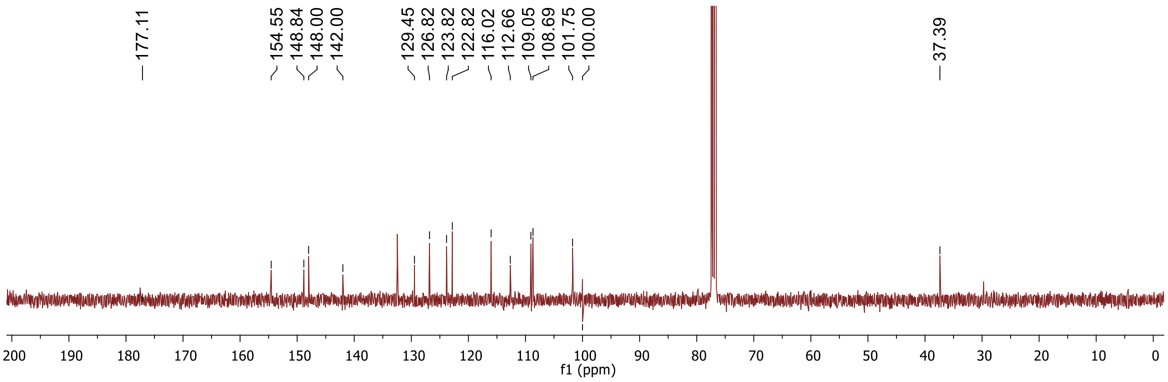

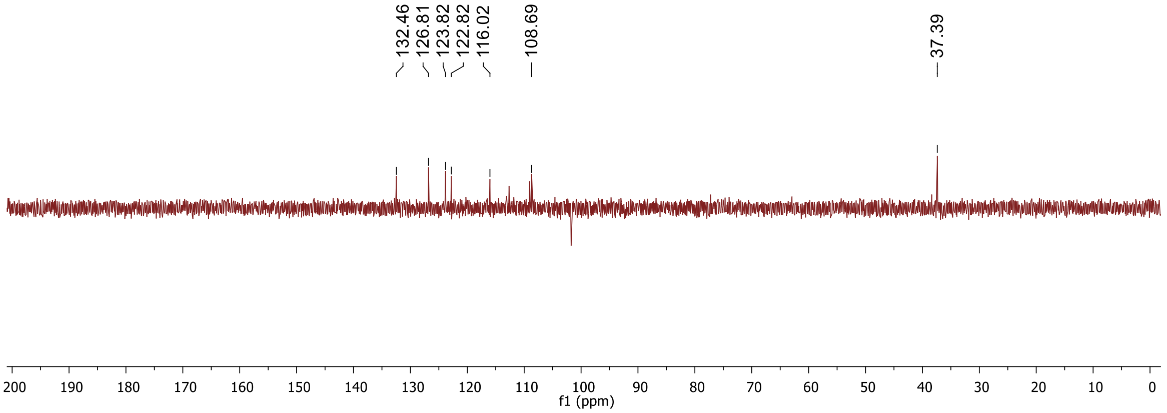

24a

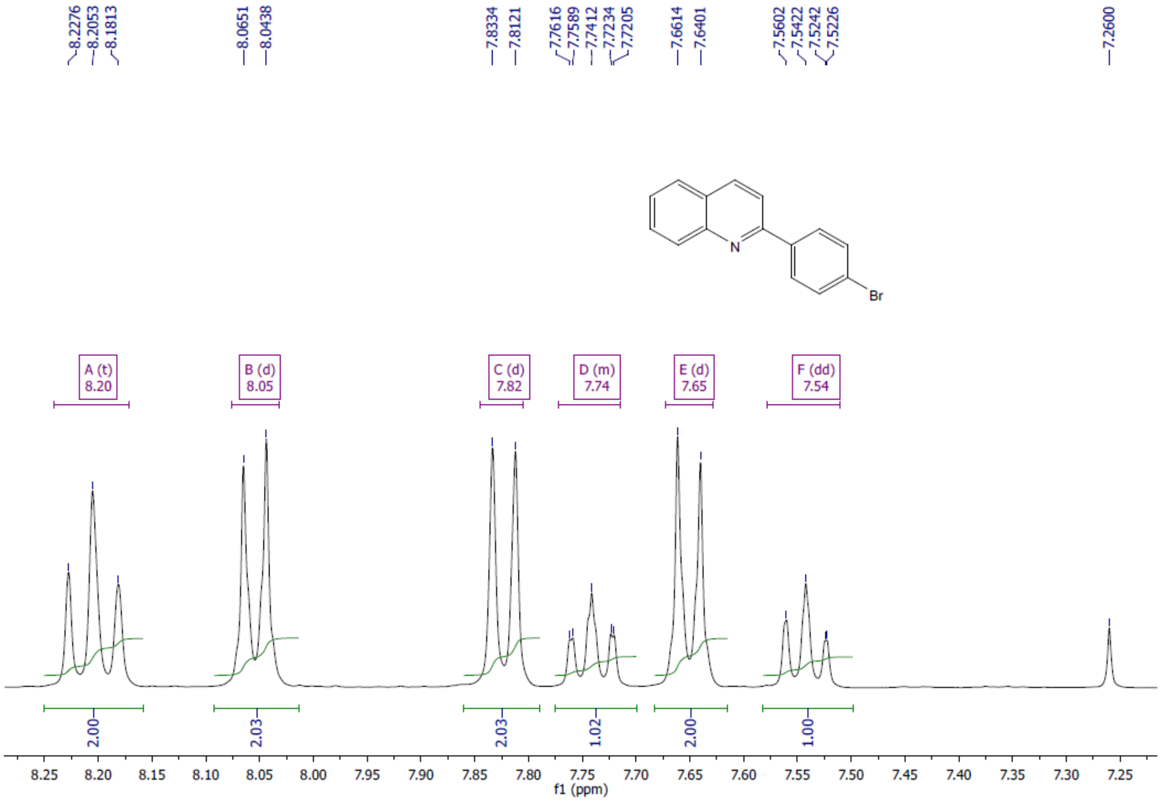
8.2276
8.2053
8.1813
8.0651
8.0438
7.8334
7.8121
7.7616
7.7589
7.7412
7.7234
7.7205
7.6614
7.6401
7.5602
7.5422
7.5242
7.5226
7.2600
N
Br
A (t) 8.20
B (d) 8.05
C (d) 7.82
D (m) 7.74
E (d) 7.65
F (dd) 7.54
2.00
2.03
2.03
1.02
2.00
1.00
f1 (ppm)

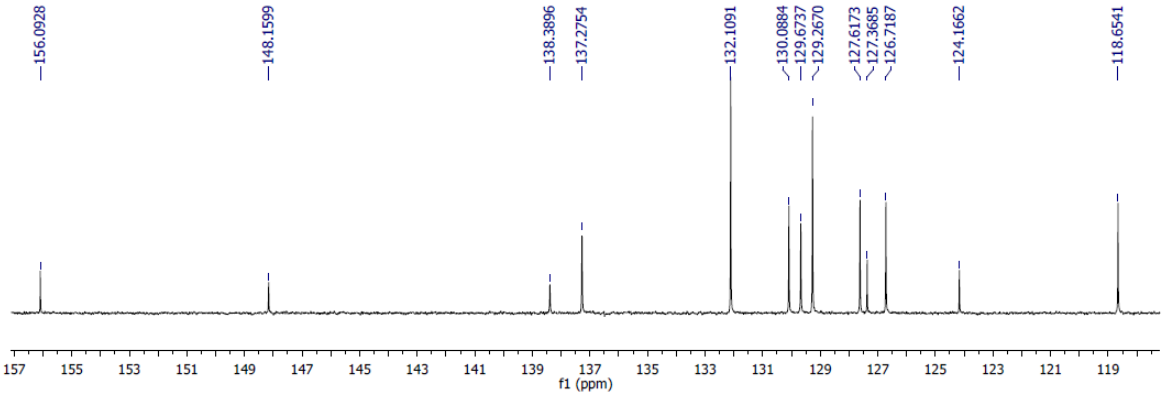
156.0928
148.1599
138.3896
137.2754
132.1091
130.0884
129.6737
129.2670
127.6173
127.3685
126.7187
124.1662
118.6541
f1 (ppm)

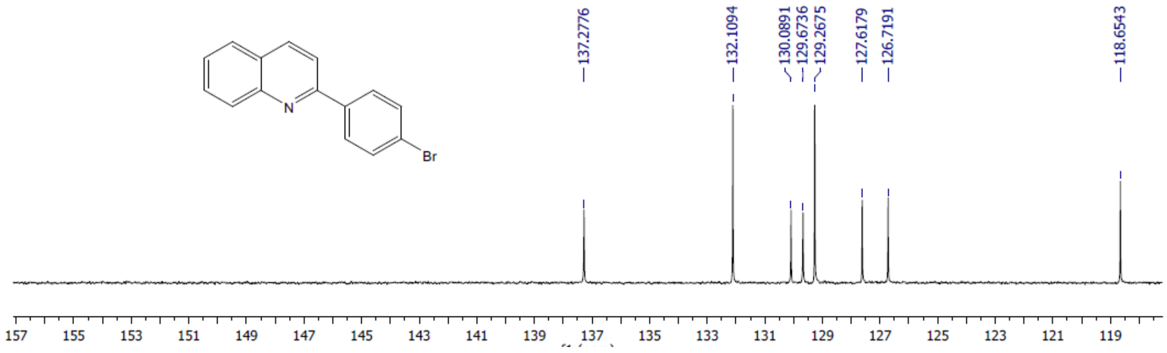
137.2776
132.1094
130.0891
129.6736
129.2675
127.6179
126.7191
118.6543
N
Br

24b

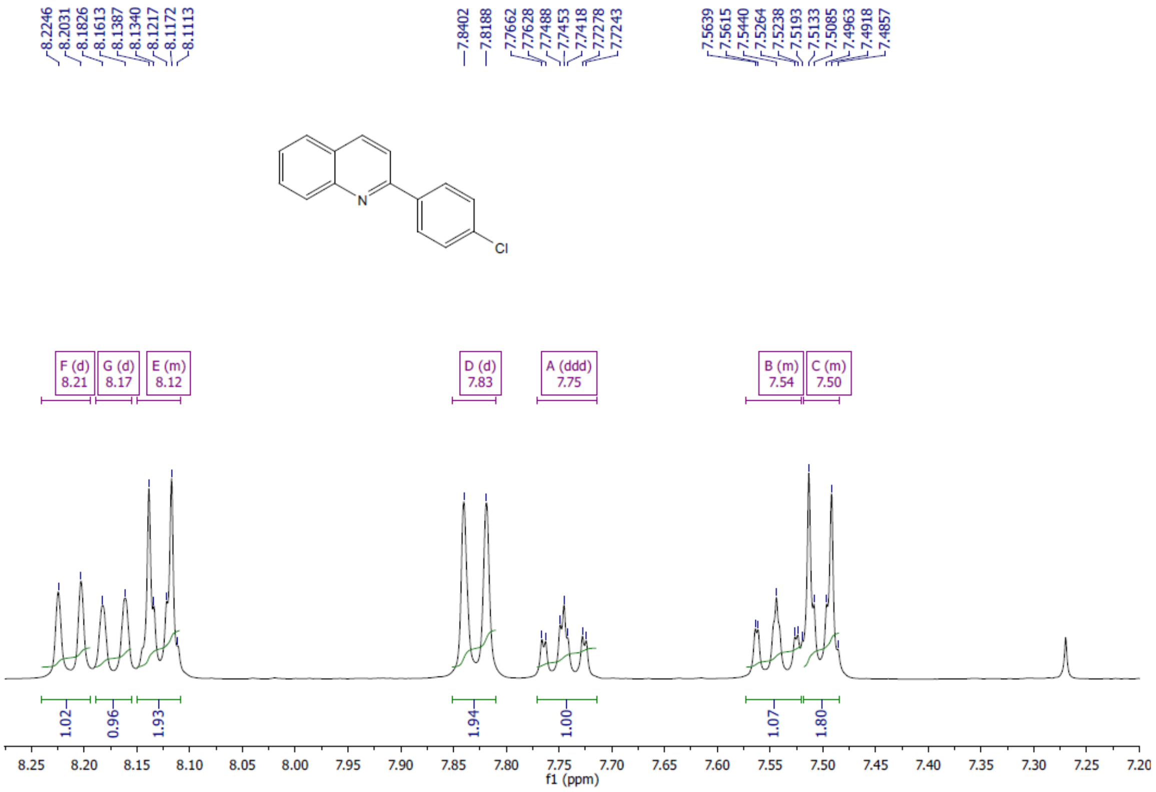

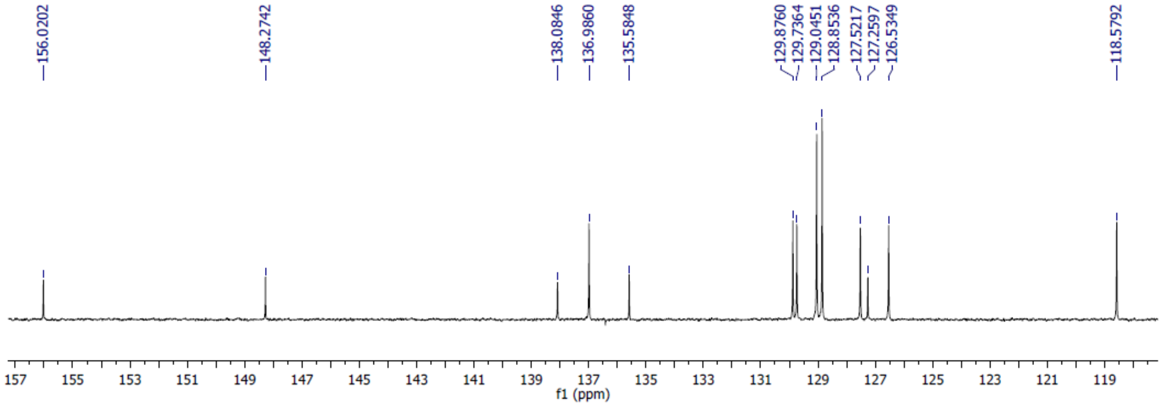

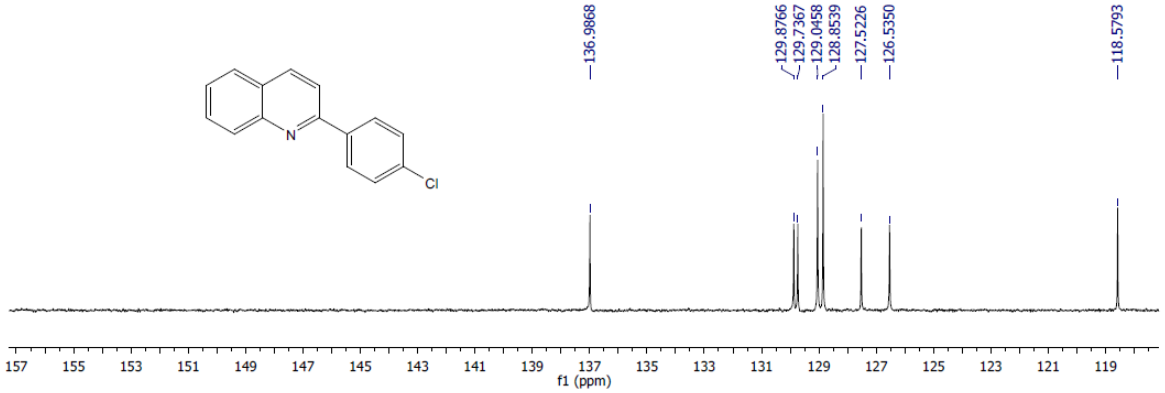

24c

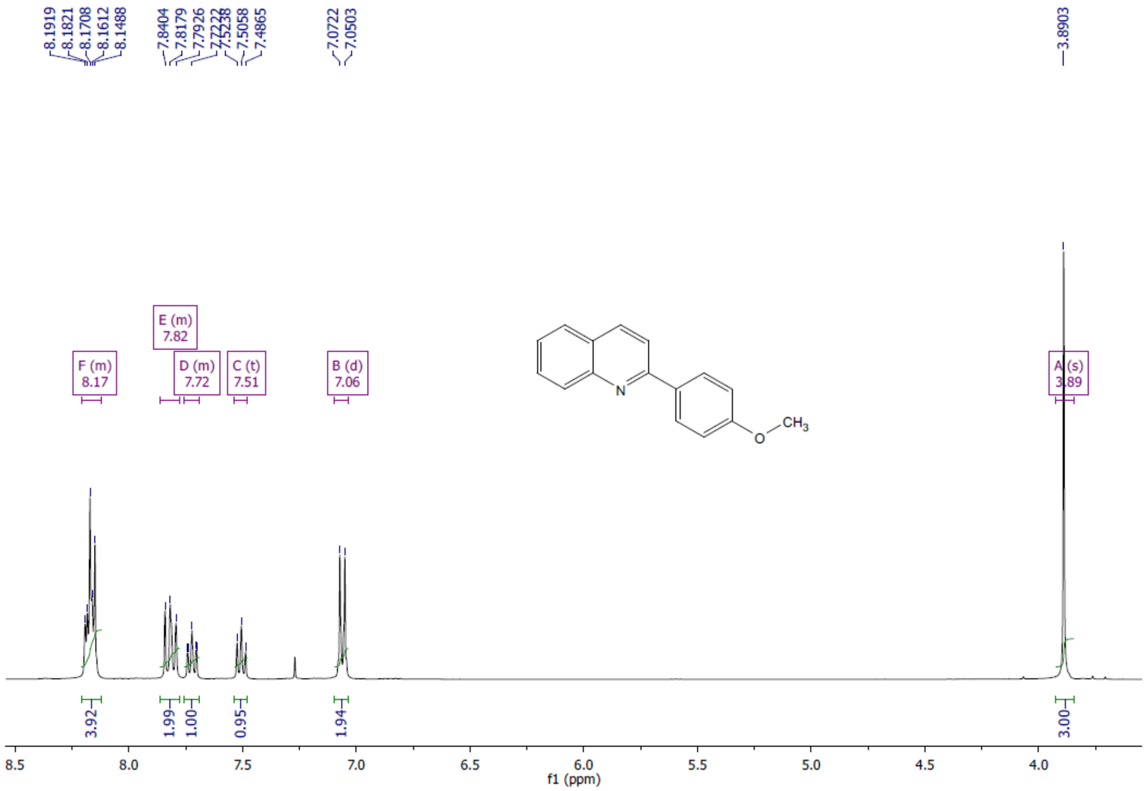
8.1919
8.1821
8.1708
8.1612
8.1488
7.8404
7.8179
7.7926
7.5058
7.4865
7.0722
7.0503
3.8903
F (m)
8.17
E (m)
7.82
D (m)
7.72
C (t)
7.51
B (d)
7.06
A (s)
3.89
N
O
CH3
3.92
1.99
1.00
0.95
1.94
3.00
f1 (ppm)

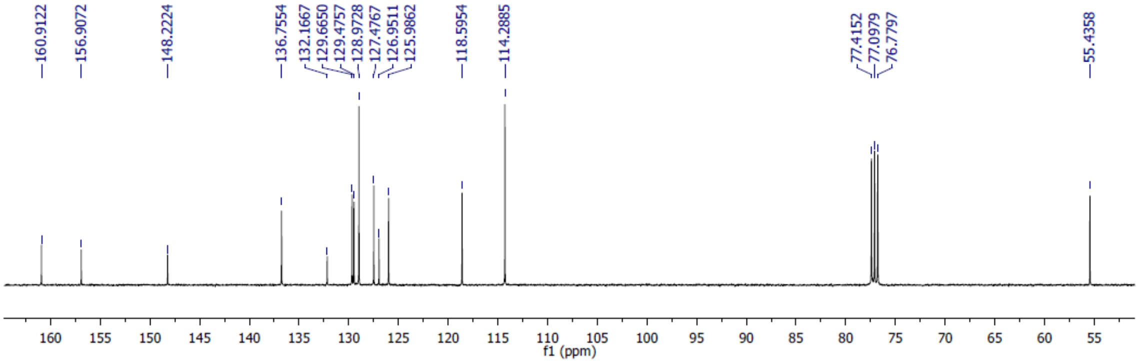
160.9122
156.9072
148.2224
136.7554
132.1667
129.6650
129.4757
128.9728
127.4767
126.9511
125.9862
118.5954
114.2885
77.4152
77.0979
76.7797
55.4358
f1 (ppm)

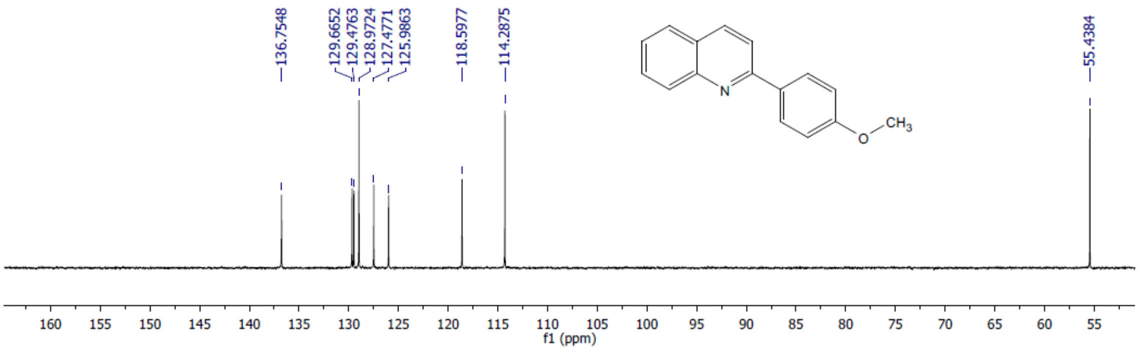
136.7548
129.6652
129.4763
128.9724
127.4771
125.9863
118.5977
114.2875
55.4384
N
O
CH3
f1 (ppm)

24d

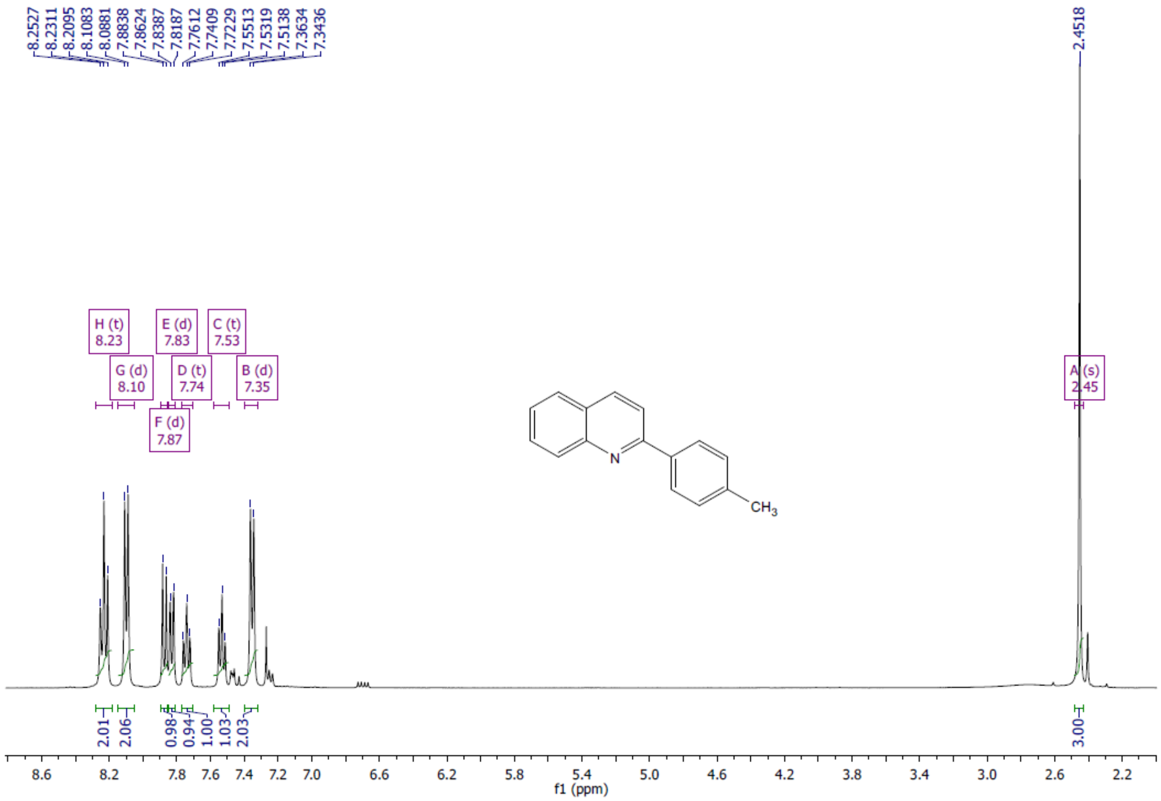
8.2527
8.2311
8.2095
8.1083
8.0881
7.8838
7.8624
7.8387
7.8187
7.7612
7.7409
7.7229
7.5513
7.5319
7.5138
7.3634
7.3436
2.4518
H (t) 8.23
G (d) 8.10
F (d) 7.87
E (d) 7.83
D (t) 7.74
C (t) 7.53
B (d) 7.35
A (s) 2.45
N
CH3
2.01
2.06
0.98
0.94
1.00
1.03
2.03
3.00
f1 (ppm)

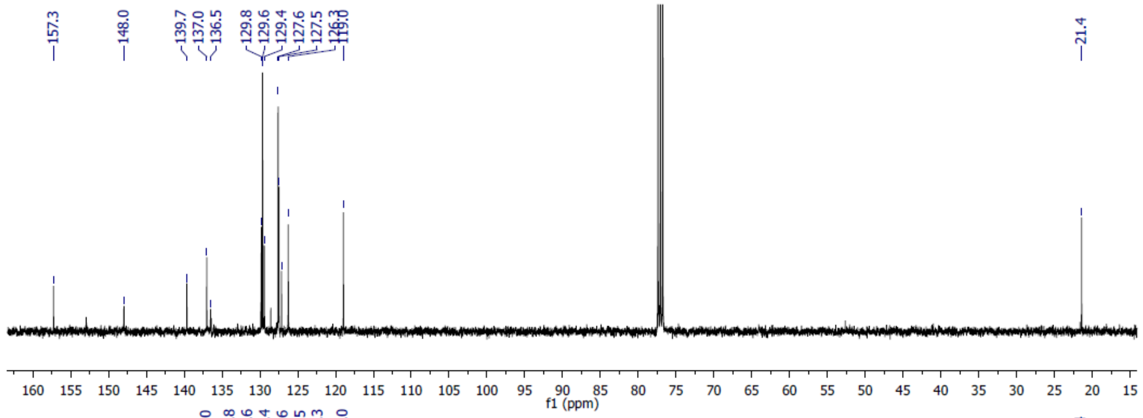
157.3
148.0
139.7
137.0
136.5
129.8
129.6
129.4
127.6
127.5
126.3
119.0
21.4
f1 (ppm)

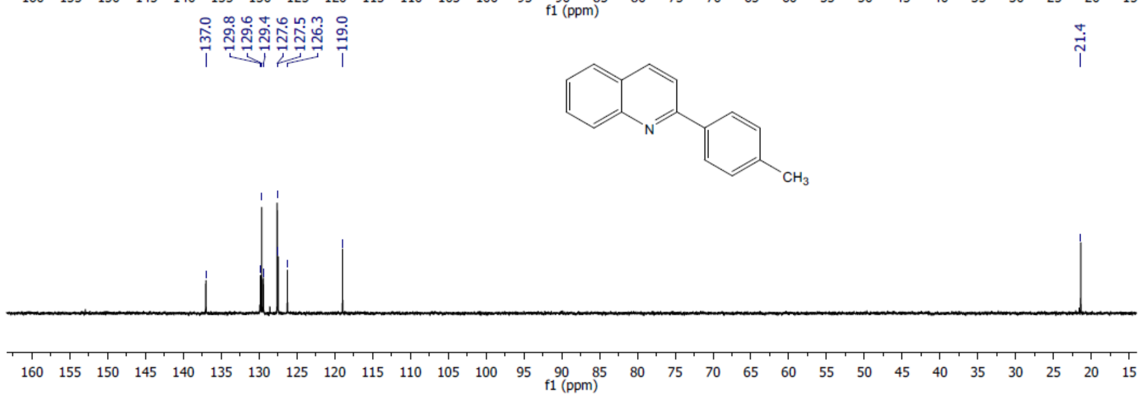
137.0
129.8
129.6
129.4
127.6
127.5
126.3
119.0
21.4
N
CH3
f1 (ppm)

24e

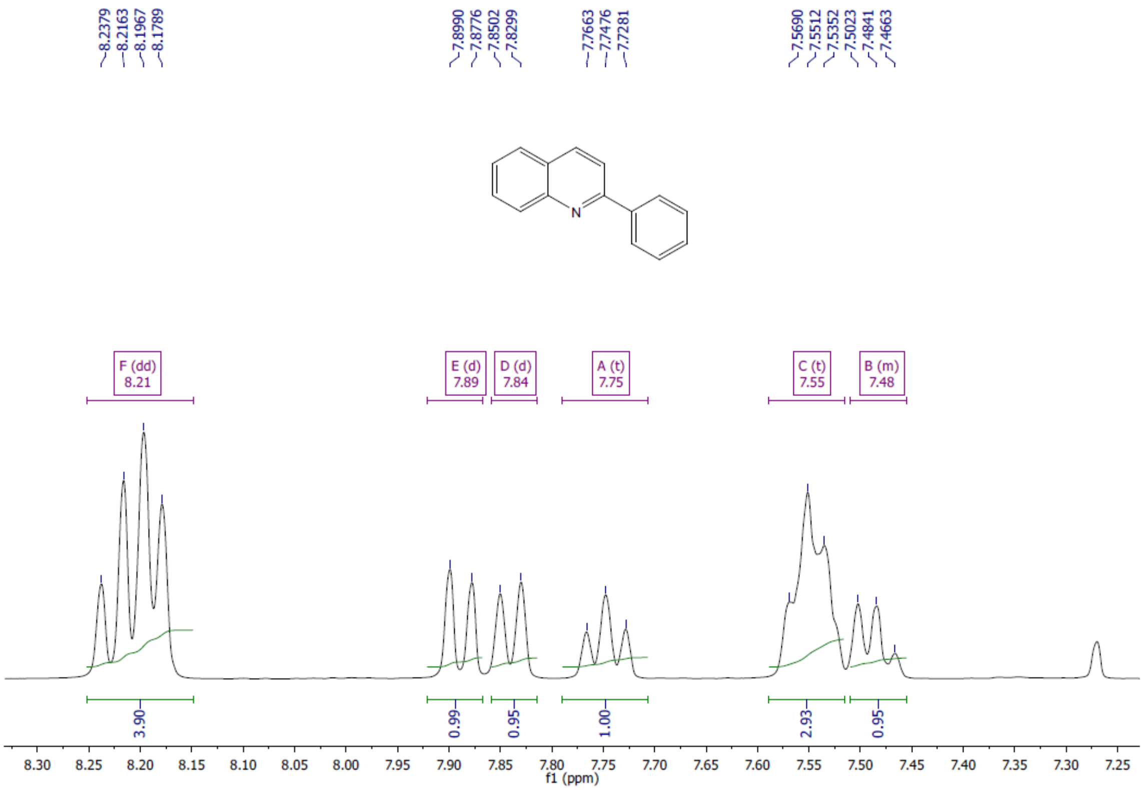
8.2379
8.2163
8.1967
8.1789
7.8990
7.8776
7.8502
7.8299
7.7663
7.7476
7.7281
7.5690
7.5512
7.5352
7.5023
7.4841
7.4663
N
F (dd) 8.21
E (d) 7.89
D (d) 7.84
A (t) 7.75
C (t) 7.55
B (m) 7.48
3.90
0.99
0.95
1.00
2.93
0.95
8.30 8.25 8.20 8.15 8.10 8.05 8.00 7.95 7.90 7.85 7.80 7.75 7.70 7.65 7.60 7.55 7.50 7.45 7.40 7.35 7.30 7.25
f1 (ppm)

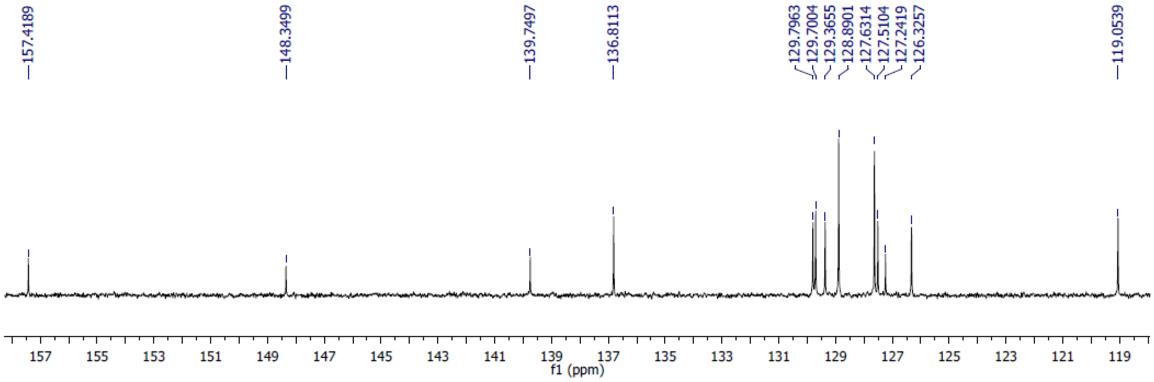
157.4189
148.3499
139.7497
136.8113
129.7963
129.7004
129.3655
128.8901
127.6314
127.5104
127.2419
126.3257
119.0539
157 155 153 151 149 147 145 143 141 139 137 135 133 131 129 127 125 123 121 119
f1 (ppm)

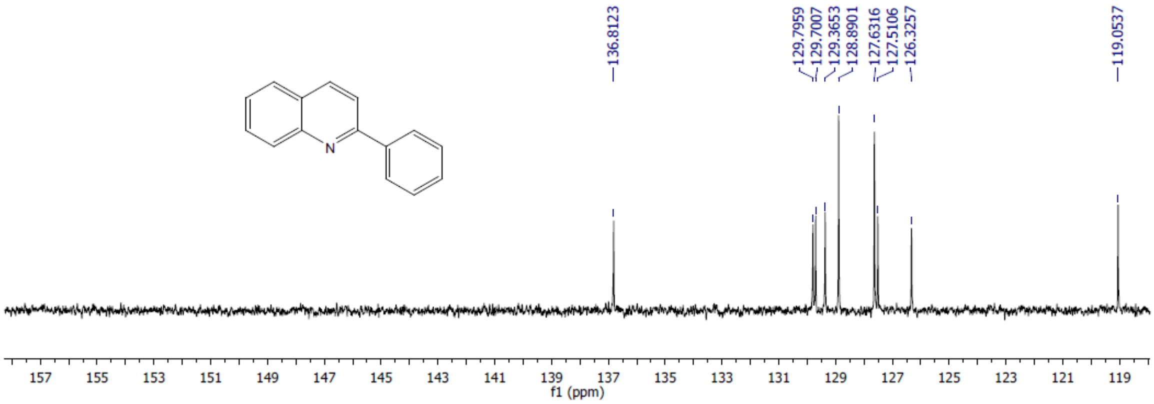
136.8123
129.7959
129.7007
129.3653
128.8901
127.6316
127.5106
126.3257
119.0537
N
157 155 153 151 149 147 145 143 141 139 137 135 133 131 129 127 125 123 121 119
f1 (ppm)

24f

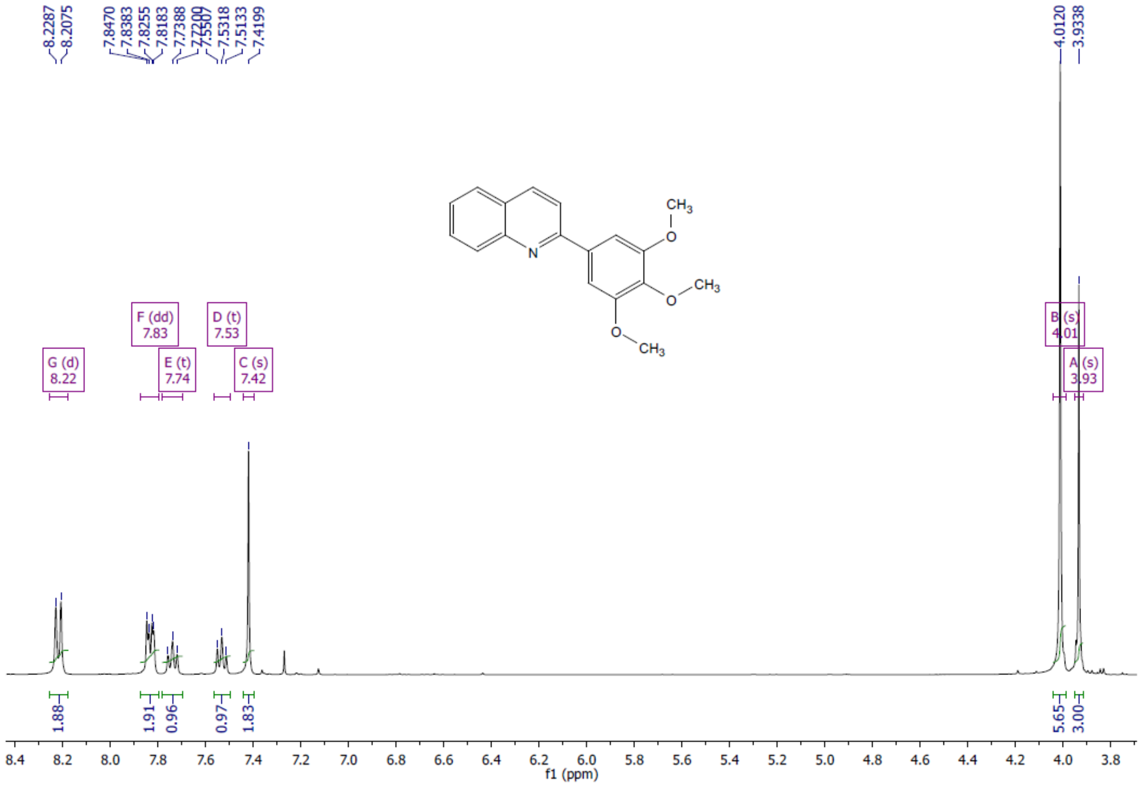
8.2287
8.2075
7.8470
7.8383
7.8255
7.8183
7.7388
7.5318
7.5133
7.4199
4.0120
3.9338
N
CH_3
O
G (d) 8.22
F (dd) 7.83
E (t) 7.74
D (t) 7.53
C (s) 7.42
B (s) 4.01
A (s) 3.93
1.88
1.91
0.96
0.97
1.83
5.65
3.00
f1 (ppm)

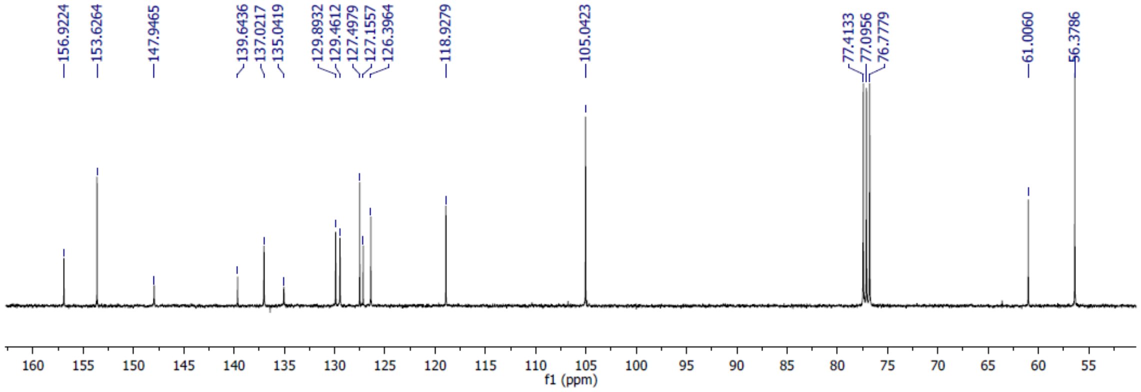
156.9224
153.6264
147.9465
139.6436
137.0217
135.0419
129.8932
129.4612
127.4979
127.1557
126.3964
118.9279
105.0423
77.4133
77.0956
76.7779
61.0060
56.3786
f1 (ppm)

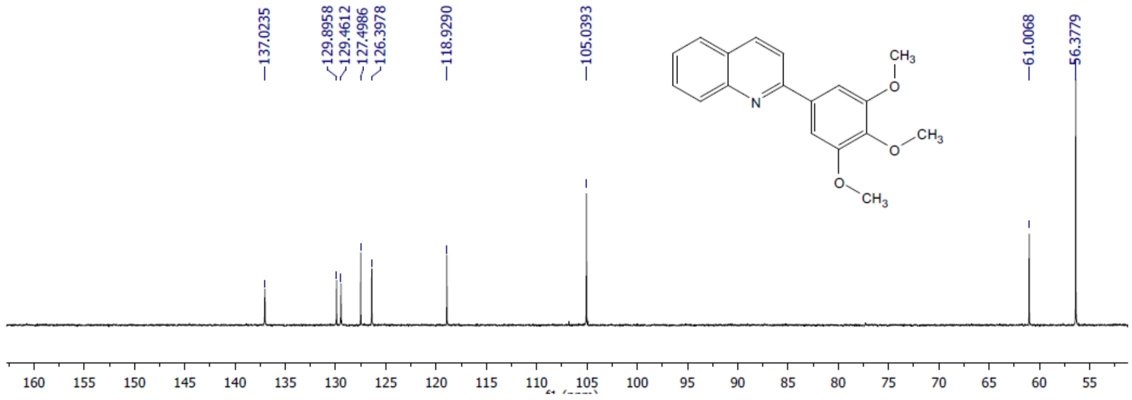
137.0235
129.8958
129.4612
127.4986
126.3978
118.9290
105.0393
61.0068
56.3779
N
CH_3
O

24g

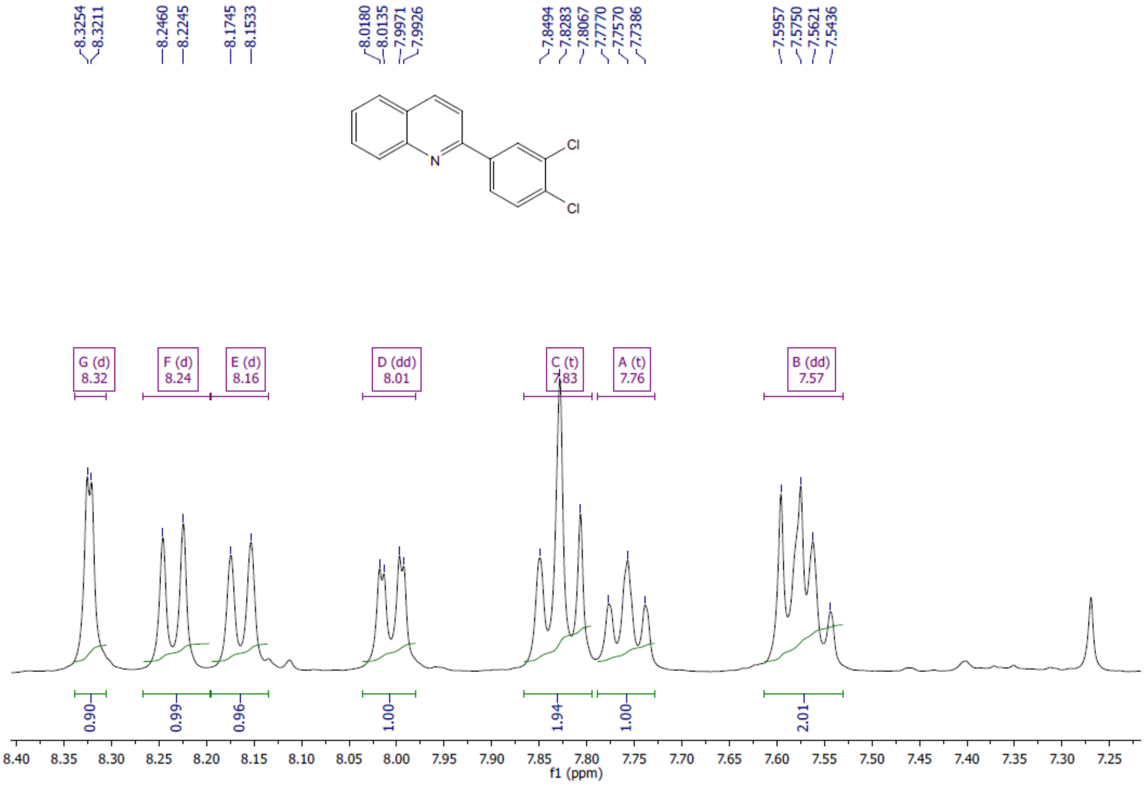
8.3254
8.3211
8.2460
8.2245
8.1745
8.1533
8.0180
8.0135
7.9971
7.9926
7.8494
7.8283
7.8067
7.7770
7.7570
7.7386
7.5957
7.5750
7.5621
7.5436
N
Cl
Cl
G (d) 8.32
F (d) 8.24
E (d) 8.16
D (dd) 8.01
C (t) 7.83
A (t) 7.76
B (dd) 7.57
0.90
0.99
0.96
1.00
1.94
1.00
2.01
f1 (ppm)

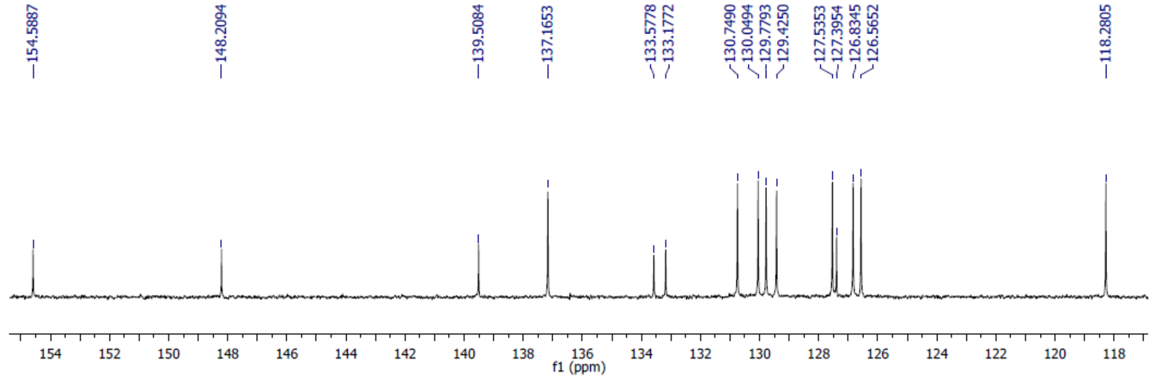
154.5887
148.2094
139.5084
137.1653
133.5778
133.1772
130.7490
130.0494
129.7793
129.4250
127.5353
127.3954
126.8345
126.5652
118.2805
f1 (ppm)

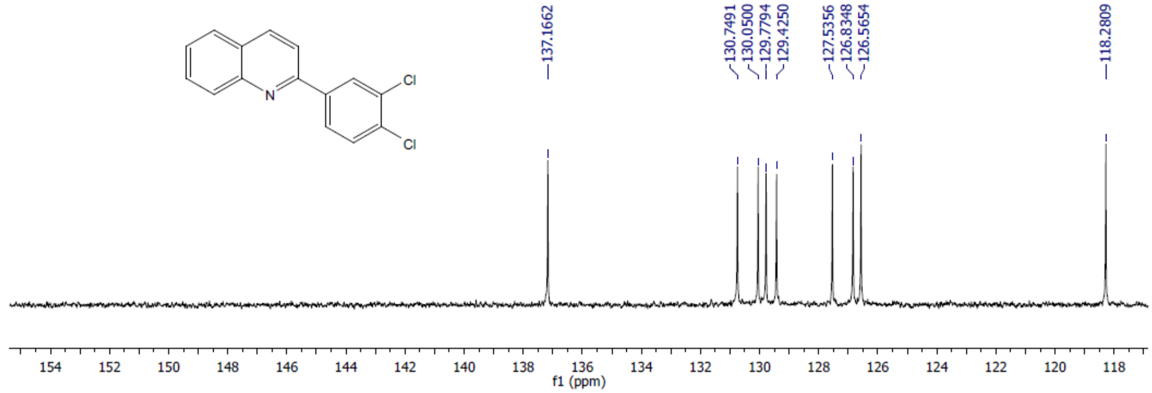
137.1662
130.7491
130.0500
129.7794
129.4250
127.5356
126.8348
126.5654
118.2809
N
Cl
Cl
f1 (ppm)

24h

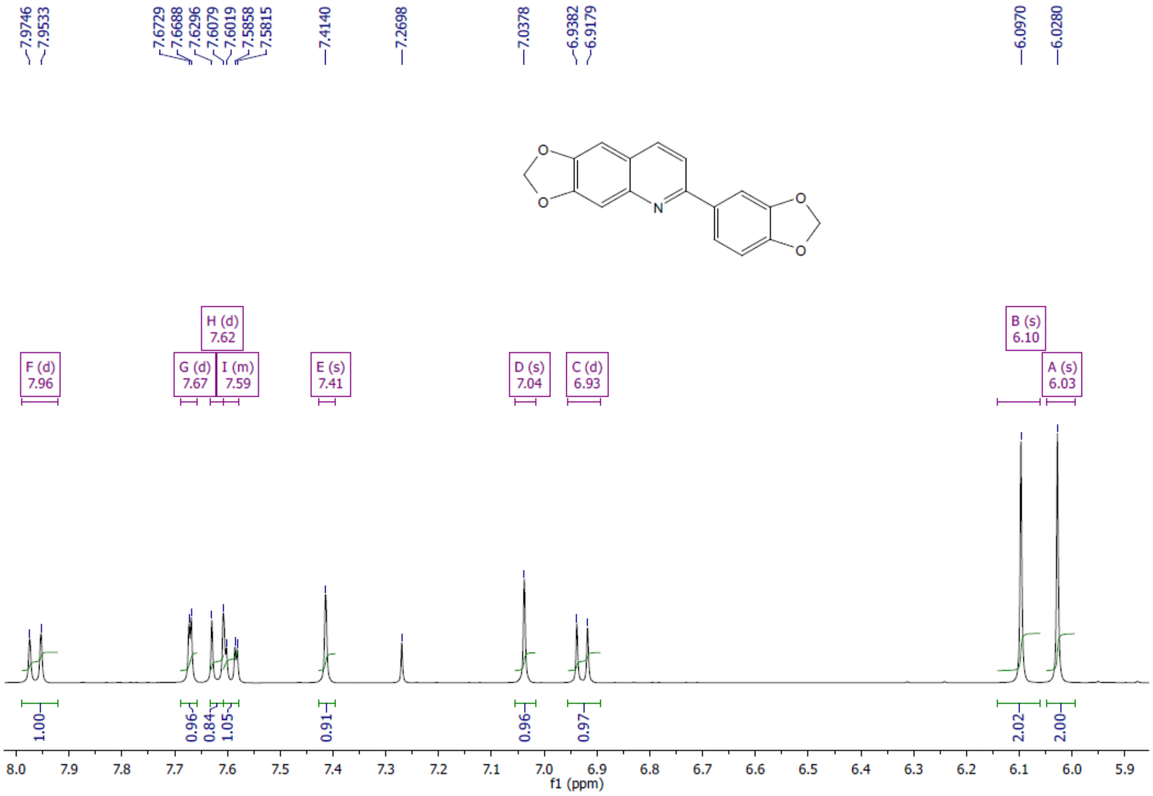
7.9746
7.9533
7.6729
7.6688
7.6296
7.6079
7.6019
7.5858
7.5815
7.4140
7.2698
7.0378
6.9382
6.9179
6.0970
6.0280
N
O
O
O
O
F (d) 7.96
G (d) 7.67
H (d) 7.62
I (m) 7.59
E (s) 7.41
D (s) 7.04
C (d) 6.93
B (s) 6.10
A (s) 6.03
1.00
0.96
0.84
1.05
0.91
0.96
0.97
2.02
2.00
f1 (ppm)

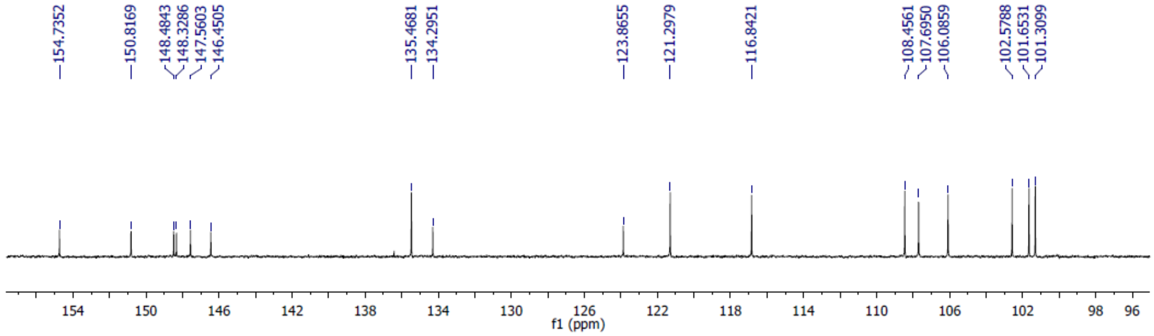
154.7352
150.8169
148.4843
148.3286
147.5603
146.4505
135.4681
134.2951
123.8655
121.2979
116.8421
108.4561
107.6950
106.0859
102.5788
101.6531
101.3099
f1 (ppm)

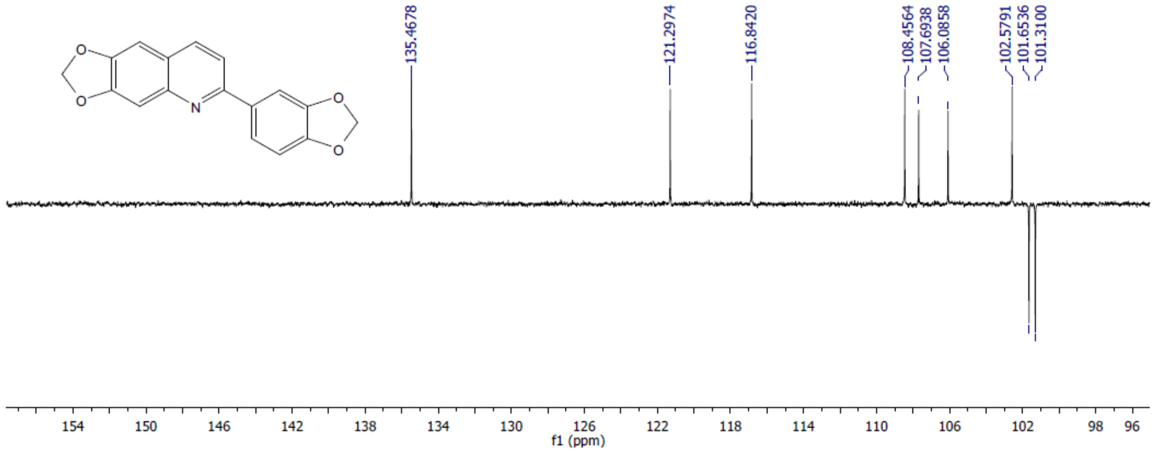
135.4678
121.2974
116.8420
108.4564
107.6938
106.0858
102.5791
101.6536
101.3100
N
O
O
O
O
f1 (ppm)

Dubamine 2

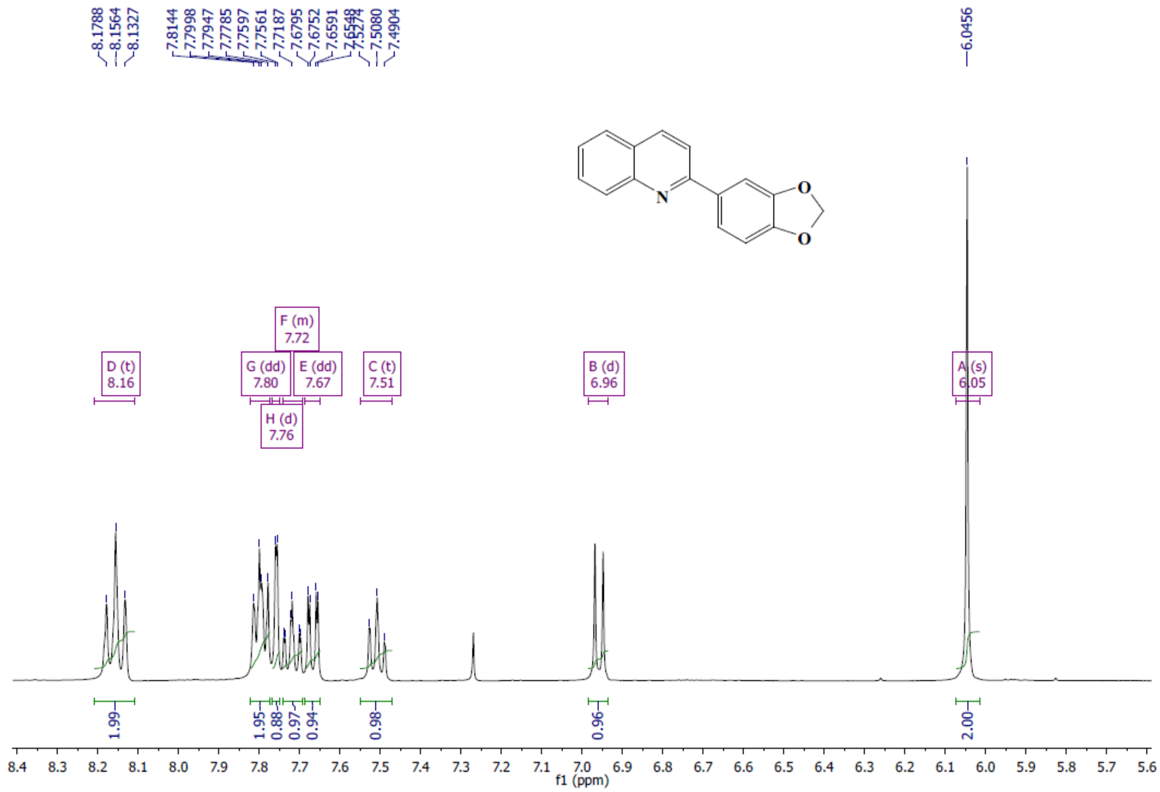
8.1788
8.1564
8.1327
7.8144
7.7998
7.7947
7.7785
7.7597
7.7561
7.7187
7.6795
7.6752
7.6591
7.6548
7.5274
7.5080
7.4904
6.0456
N
O
O
F (m) 7.72
D (t) 8.16
G (dd) 7.80
E (dd) 7.67
C (t) 7.51
B (d) 6.96
A (s) 6.05
H (d) 7.76
1.99
1.95
0.88
0.97
0.94
0.98
0.96
2.00
f1 (ppm)

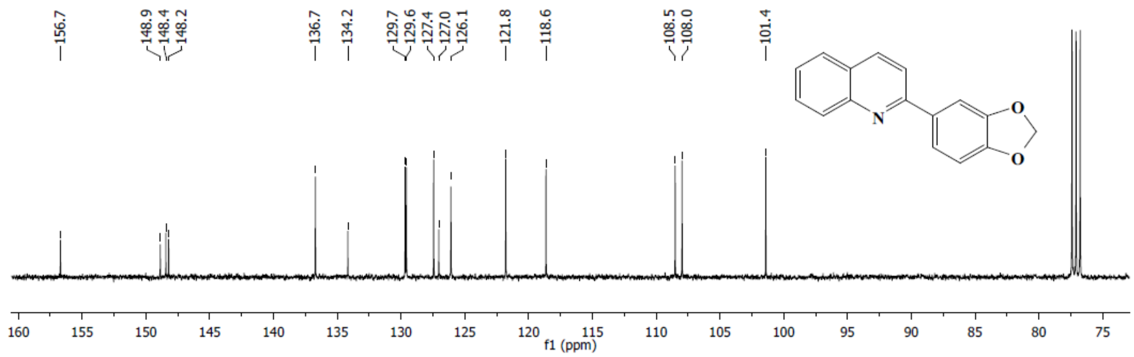
156.7
148.9
148.4
148.2
136.7
134.2
129.7
129.6
127.4
127.0
126.1
121.8
118.6
108.5
108.0
101.4
N
O
O
f1 (ppm)

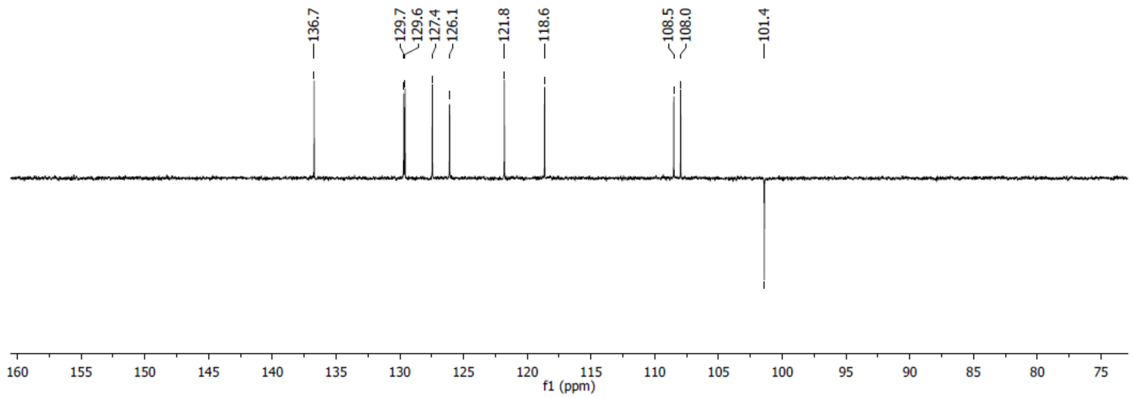
136.7
129.7
129.6
127.4
126.1
121.8
118.6
108.5
108.0
101.4
f1 (ppm)

26a

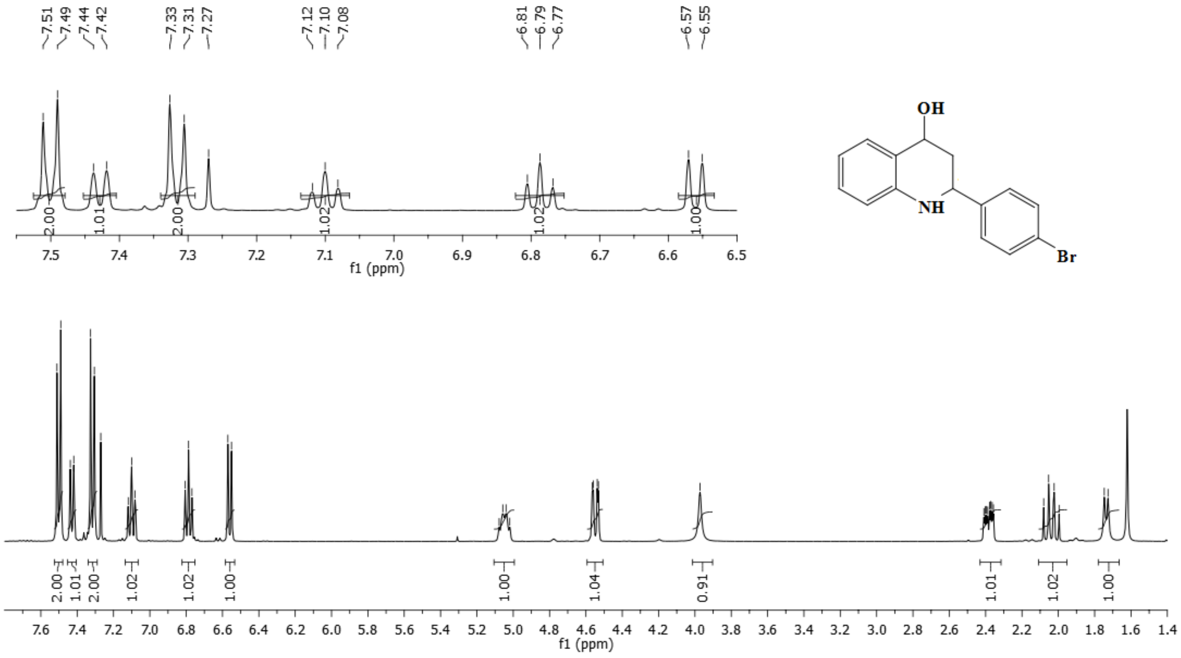

Supplement: Supplementary file 1 [file molecules-29-01959-s001.zip › molecules-2900633-supplementary.pdf]
